# Cross-sectional study of female sex workers in Soweto, South Africa: Factors associated with HIV infection

**DOI:** 10.1371/journal.pone.0184775

**Published:** 2017-10-05

**Authors:** Jenny Coetzee, Rachel Jewkes, Glenda E. Gray

**Affiliations:** 1 Perinatal HIV Research Unit, University of the Witwatersrand, Chris Hani Baragwanath Academic Hospital, Johannesburg, South Africa; 2 Department of Public Health, University of the Witwatersrand, Johannesburg, South Africa; 3 Gender & Health Research Unit, South African Medical Research Council, Pretoria, South Africa; 4 Office of the President, South African Medical Research Council, Cape Town, South Africa; University of Ottawa, CANADA

## Abstract

**Introduction:**

In South Africa, the rate of HIV in the sex worker (SW) population is exceedingly high, but critical gaps exist in our understanding of SWs and the factors that make them vulnerable to HIV. This study aimed to estimate HIV prevalence among female sex workers (FSWs) in Soweto, South Africa, and to describe their sexual behavior and other factors associated with HIV infection.

**Methods:**

A cross-sectional, respondent-driven sampling (RDS) recruitment methodology was used to enroll 508 FSWs based in Soweto. Data were collected using a survey instrument, followed by two HIV rapid tests. Raw and RDS adjusted data were analyzed using a chi-squared test of association and multivariate logistic regression to show factors associated with HIV infection.

**Findings:**

HIV prevalence among FSWs was 53.6% (95% CI 47.5–59.9). FSWs were almost exclusively based in taverns (85.6%) and hostels (52.0%). Less than a quarter (24.4%) were under 25 years of age. Non-partner violence was reported by 55.5%, 59.6% of whom were HIV-infected. Advancing age, incomplete secondary schooling, migrancy and multiple clients increased the likelihood of HIV acquisition: >30 years of age was associated with a 4.9 times (95% CI 2.6–9.3) increased likelihood of HIV; incomplete secondary schooling almost tripled the likelihood (AOR 2.8, 95% CI 1.6–5.0); being born outside of the Gauteng province increased the likelihood of HIV 2.3 times (95% CI 1.3–4.0); and having more than five clients per day almost doubled the likelihood (AOR 1.9, 95% CI 1.1–3.2).

**Conclusion:**

Our findings highlight the extreme vulnerability of FSWs to HIV. Advancing age, limited education and multiple clients were risk factors associated with HIV, strongly driven by a combination of structural, biological and behavioral determinants. Evidence suggests that interventions need to be carefully tailored to the varying profiles of SW populations across South Africa. Soweto could be considered a microcosm of South Africa in terms of the epidemic of violence and HIV experienced by the SW population, which is influenced by factors often beyond an individual level of control. While describing a hitherto largely undocumented population of FSWs, our findings confirm the urgent need to scale up innovative HIV prevention and treatment programs for this population.

## Introduction

Globally, sex workers (SWs) bear a disproportionately high HIV burden [1]. Despite South Africa entering its fourth decade of the HIV epidemic, much remains unclear about the key structural drivers of the HIV epidemic among female sex workers (FSWs) [1, 2]. Defined as adult individuals who consent to sexual exchange with the primary purpose of monetary benefit [3], sex work remains criminalized in South Africa [4]. Recent studies suggest HIV prevalence of between 40% and 88.4% among this population in South Africa [5–7]. A 2013 South African Health Monitoring Survey (SAHMS) found HIV prevalence among FSWs in Johannesburg to be 71.8% [5]. In contrast, a 2012 meta-analysis revealed HIV prevalence of 11.8% (95% CI 11.6–12.0) among FSWs from 50 countries, with a pooled prevalence of 36.9% (95% CI 36.2–37.5) across sub-Saharan Africa [2]. This underscores the burden of HIV in this region, with South African FSWs demonstrating extreme vulnerability to HIV. The rate of infection among FSWs in South Africa is also significantly higher than in the general population, where prevalence was 12.2% in 2012, peaking at 25.2% in those aged 25–49 years [8].

Sex work in South Africa has been described as functioning within formal settings such as brothels and hotels, or more informally on street-based hotspots [9–11]. Limited information is available on sex work within township settings, a prominent feature of South Africa’s urban and peri-urban landscape. Evidence suggests that within South African townships, transactional sex provides income for many women of low socio-economic status, but such transactions frequently take place within an existing relationship [12]. A 2002 study described transactional sex-for-money exchanges taking place in Soweto, confirming that such exchanges were informal and ambiguous in comparison to commercial sex work. The study highlighted that these exchanges served largely as a means of survival for some women [13]. A 2004 study conducted among antenatal women demonstrated the relationship between transactional sex and HIV acquisition, with 21% of female patients in an antenatal clinic in Soweto reported to be engaging in transactional sex, increasing the risk of HIV 1.54 times (95% CI 1.07–2.21) when compared to women with no reported transactional sex relationships [14]. Further research is required to comprehensively describe the informal SW population in Soweto where sexual services are sold to both regular and once-off clients.

Low levels of education, unemployment, gender inequality, violence, migration and poverty are all structural drivers of South Africa’s heterosexual HIV epidemic [1, 8, 15, 16]. Together, these factors may also be important structural drivers of entry into the sex work profession [17–20]. A combination of the criminalization of the profession [1] and exposure to high levels of violence exacerbates FSWs’ vulnerability to HIV [21]. The prevalence of violence among FSWs is high, with 50.9% reporting physical assault and 21.9% reporting sexual assault or rape over a 12-month period, in Johannesburg, South Africa. The same study found that that 31.5% of Johannesburg-based FSWs were foreign migrants, with an HIV prevalence of 86.6% as compared with 66.0% among South African FSWs [5].

Within the general population, behavioral factors include inconsistent condom use [22, 23], early sexual debut and age disparity between partners. Sexually transmitted infections (STIs) also increase vulnerability [8, 24–27]. In the general population, age is a significant risk factor, with young women and girls bearing the greatest burden of HIV [28]. Age may also play an important role in SW vulnerability, with evidence that a third of FSWs in Johannesburg were aged 16–24 years (36.5%). Of these, 59.1% were HIV-positive [5]. While early sexual debut, and substance abuse are thought to be associated with entry into sex work [17–20], risky sexual behaviors have been cited frequently as one of the primary drivers of HIV prevalence among FSWs [4, 15, 29, 30]. These include multiple partners (both clients and intimate partners (IP)) and result in increasing exposure to wider sexual networks [8] and substance abuse [5, 31]. Evidence has shown that as many as 81.5% of FSWs in Johannesburg, South Africa engage in binge drinking [5]. Alcohol has been associated with both HIV and unsafe sexual practices [21]. Various sexual practices, including limited or inconsistent condom use [32, 33] and unprotected anal sex [34], increase vulnerability. Unprotected anal sex increases HIV infection by between 1.4 [35] and 5.1 [36] times, with as many as 40% of FSWs along a South African trucking route reporting engaging in anal sex [34]. Dry sex or douching practices, which include inserting various drying or astringent agents into the vagina, can lead to lesions, ulceration and altered pH-levels. These factors increase vulnerability to STIs and possibly HIV[30, 37–39].

An understanding of such micro- through macro-level factors is crucial to the effective development of HIV prevention and treatment programs for this key population. It is, furthermore, vital to understand specific contextual dynamics and the profile of the local sub-population for effective program tailoring. However, limited data is available on areas where sex work was not traditionally believed to exist, such as in township settings.

South Africa is currently in the process of scaling up the *South African National SW HIV Plan 2016–2020*, which has secured commitments for significant funding [3]. Between 1998 and 2016, programs were geared towards reducing SW vulnerability to HIV by focusing heavily on basic peer education, condom negotiation and HIV testing. To ensure that SW HIV programs are designed effectively to minimize risk and maximize impact, it is vital that more knowledge is generated on HIV prevalence and associated risk factors for HIV vulnerability. This is especially important in settings where sex work has been uncovered. Our study aims to estimate HIV prevalence among FSWs within Soweto, and to further describe sexual risk factors and their association with HIV vulnerability in this population.

## Method

This cross-sectional study formed part of a larger project aimed at understanding the complex interplay of factors associated with HIV infection for FSWs in Soweto. The study drew on an ecological model [40] of HIV risk whereby SWs may have been made vulnerable to HIV owing to individual factors (e.g. age or level of education), factors pertaining to their relationship with their IP (e.g. condom use), factors relating to their work (e.g. number of clients) and community/societal factors (e.g. policing or laws related to sex work). For this analysis, we focused on factors measured at the individual level, but which reflect on all levels.

The study was conducted in Soweto, a township on the outskirts of Johannesburg, South Africa. Soweto is predominantly urban and peri-urban, low-income with limited educational and employment opportunities. It has the highest population density in South Africa, comprises more than 40 suburbs within 61 km^2^, and is estimated to house over two million inhabitants. Soweto has approximately 3 000 drinking establishments (legal and illegal), with R50 million (USD 3.8 million) spent annually on beer. Eleven apartheid-era, formerly single-sex, ethnically segregated hostels house an estimated 40 000 residents. Hostels have a highly politicized and violent history, with hostel dwellers never becoming fully integrated into suburban, township life [41]. Despite encouraging family units to move into the hostels post-apartheid, hostels have remained a source of unrest and crime owing to serious overcrowding, poverty and South Africa’s complex socio-political history. These hostels comprise a highly patriarchal environment, valuing an African culture of hegemonic masculinity aimed at the subordination of women [42] and silence surrounding sexuality [43], giving rise to high levels of violence [43, 44]. It is from within these hostel and tavern environments in Soweto that sex work takes place.

A sex work program launched in October 2013 by a Soweto-based research organization was built on a peer education model and embraced community participation. It provides outreach services, human rights knowledge dissemination, psychosocial counselling, condom distribution, HIV testing, twice-monthly risk reduction workshops, and connections to care networks. In June 2016, the program launched a primary healthcare service which included the provision of pre-exposure prophylaxis (PrEP) for HIV-negative SWs. Their fixed clinic was used as a base for the research, with interviews conducted in private counselling rooms [45].

FSWs and local SW activist organizations were actively engaged throughout the study design and development phases, through various formal and informal discussion platforms. Sex work peer educators (n = 10) were identified through the local sex work program and trained in basic lay counselling, trauma containment and debriefing, good clinical practice, research data collection, and study-specific training, totaling 5 000 hours between 2014 and 2015. These counsellors were used within both the pilot [45] and main study to conduct screening, enrollment, survey interviews, HIV counselling and testing, and to oversee study recruitment.

Formative work included cognitive interviews with 12 conveniently sampled FSWs, to ensure that the survey instrument was understandable and appropriate. This was followed by a pilot study (n = 40) of study procedures (October–November 2015) [45]. Participants within both formative components met the same inclusion criteria as the primary study, except that FSWs who did not work in Soweto were specifically targeted to participate in interviews (n = 12) and the pilot study (n = 28). Two participants who took part in the formative work were eligible for enrollment in the primary study (n = 2).

During screening, SW status was identified using three mechanisms: 1) screening by a well-trained sex work counsellor; 2) primary screening which asked about engagement in sex work in Soweto; and 3) secondary screening for persons suspected of not being a SW, using local, SW-specific discourses to probe more deeply into the individual’s involvement in sex work. Inclusion criteria for the study were: biologically female, over 18 years old, currently sold sex in Soweto, knew their recruiter, and gave voluntary informed consent to participate in both the survey and HIV testing. While engaging in sex work was a requirement, self-identification as a SW was not required. To avoid duplicate enrollment, fingerprints (left, index finger) were scanned and an algorithm recorded and compared for every enrollment using the FingerTec OFIS system (http://www.fingertec.com/images/brochure/Ofis-E.pdf).

A respondent-driven sampling (RDS) recruitment strategy was used in line with the guidelines provided by the World Health Organization [46]. This method is a popular way to recruit marginalized populations and enable population-level estimates [47, 48]. The method is geared towards overcoming sampling bias and allowing for the generalizability of findings through data weighting based on participant network size. RDS allows for estimating the proportion of the population by adapting sampling according to the realized network of the given population. This ensures a non-zero probability of inclusion in the sample and allows for the provision of asymptomatically unbiased estimates of the factors associated with HIV infection among FSWs. Much like a random sample, RDS allows for generalizability of findings. RDS assumptions were monitored using specialist software (RDS-Analyst) during data collection, to ensure that all assumptions were met and findings would be generalizable. Reciprocal relationships between recruiter and recruit, or reciprocity, were assumed based upon potential participants’ confirmation of an existing relationship between themselves and their recruiter. Homophily, or purposefully recruiting individuals with similar characteristics, was assessed using the ratio of recruits to recruiters who had a corresponding characteristic (e.g. HIV status). This served to ensure that the number of homophilous pairs was close to the equivalent in the sample population of what would be expected to occur by chance. Finally, convergence or equilibrium across the sample was assessed. This indicates that the final sample was not biased by the purposive selection of the initial recruits and, thus, potential bias of all subsequent recruits [49].

**Fig 1:**
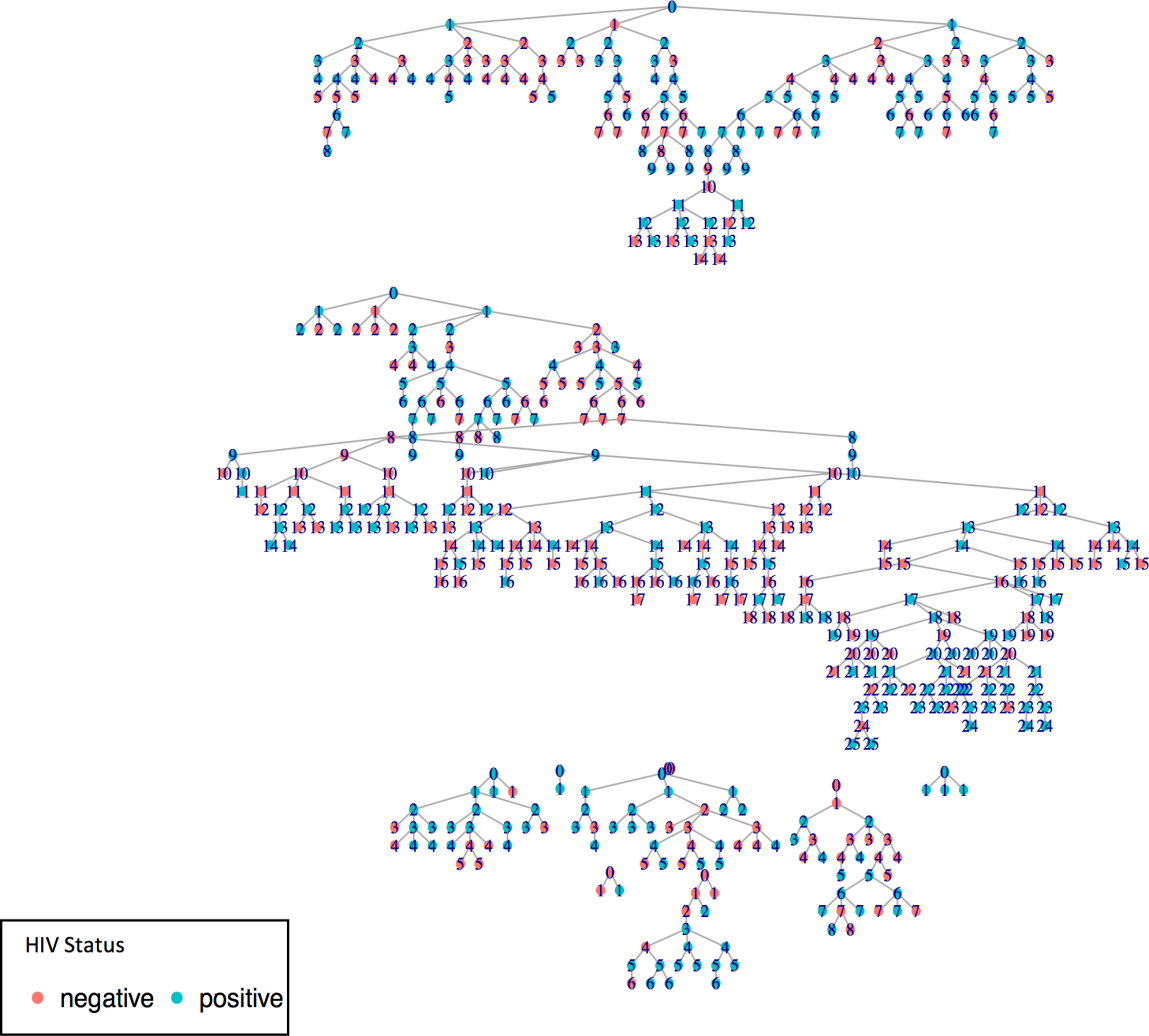
Recruitment tree indicating initial seeds recruited (0) and all their subsequent recruits, known as recruitment chains. Waves of recruitment are shown numerically radiating out from each seed (1–25). An HIV-positive status is highlighted in blue.

In determining sample size, a two-sided calculation was used to detect a difference between HIV prevalence among FSWs presumed to be exposed to violence versus those unexposed to violence. A sample size of 500 was estimated to ensure sufficient power of analysis. Recruitment was undertaken between February and September 2016 (n = 508). Initial participants (seeds) were identified by fellow FSWs as being well networked within the local FSW community, during workshop sessions held monthly in the local sex work program. All seeds were required to have a minimum network of 15 other FSWs that they knew and had seen within the preceding two weeks. They were diverse in age (21–38 years), and worked across different suburbs within Soweto. Four seeds were recruited at the start of the study. Due to several consecutive public holidays in South Africa, recruitment slowed substantially in weeks 7–11, and a further seven seeds were located using existing participants and sex work counsellors/peer educators from geographic areas not yet reached by the sex work program. They were introduced into the study between weeks 13 and 15. This had the advantage of increased diversity among the seed sample and allowed for seeds to be selected who had not been part of the local sex work program. Similar to a chain referral method [50], all participants (including seeds) were given three coupons with which to recruit potential participants. Seeds were asked to recruit FSWs they knew, of varying ages and from multiple sites across Soweto. Potential participants then presented at the site, and once screened and if enrolled, formed the first wave of the study. Once data collection was completed, they were given three coupons with which to recruit future potential participants. Thus, each set of coupons led to subsequent waves of recruitment. All non-seed recruiters were asked to give the coupons only to randomly selected women they knew and who knew them, who, like themselves, sold sex in Soweto, and who were older than 18. Recruitment chains were mapped between each seed and all subsequent recruitments [47, 48] (Fig 1). To stop enrollment, no further coupons were distributed after the 498th enrollment.

**Fig 2:**
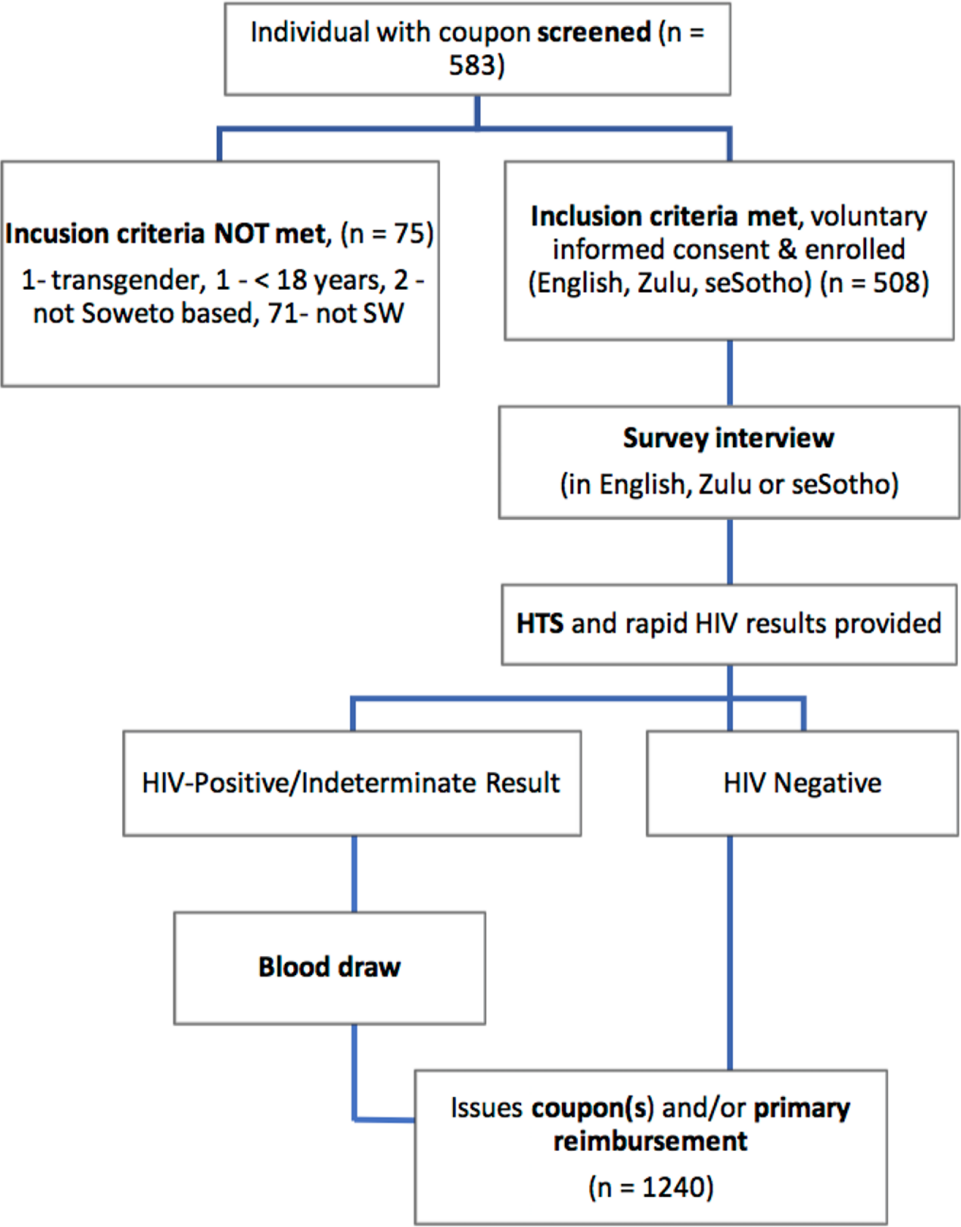
Study flow diagram showing successful enrollment as well as the number of coupons distributed during the study.

After screening, participants gave their consent to participate in the study, and then completed a 45-minute, interviewer-administered questionnaire in English, Zulu or seSotho. This was followed by HIV testing and counselling with two HIV rapid tests (Fig 2). After data collection, participants were given a maximum of three uniquely coded coupons and reimbursed R100.00 ($7.69). A further R20.00 ($1.56) secondary incentive for every one of their coupons that led to a successful enrollment was paid 7–10 days after the recruiters’ enrollment date. Primary reimbursement and secondary incentives were increased five months into the study to cover additional costs to participate, by a maximum of R20.00 each ($1.56), owing to more widespread geographic recruitment and increased cost of transportation.

Data were captured directly onto Lenovo tablets using the REDCap electronic data management system [51], hosted by the University of the Witwatersrand, South Africa. The database housed built-in skip patterns and algorithms, highlighting missing values before an interviewer could move to the next survey section. Data could be viewed live on the REDCap database to ensure that queries were addressed swiftly before participants left the study site. The system also enabled interviewers to insert detailed comments or explanations on responses they wished to discuss. A 48-hour response-time for checking questionnaires and closing queries was applied during the study. This helped to ensure high quality data were captured consistently. Duplicate data were collected on a convenience sample of 12% of the final sample size with an error rate of 0.6%. Ethical approval was provided by the Human Research Ethics Committee (Medical) of the University of the Witwatersrand, South Africa.

## Measures

Coupon numbers were recorded and linked between seeds, recruiters and subsequent recruits (Fig 1). Participants were asked a three-part question to obtain their network size: “How many FSWs do you know, who also know you, in Soweto? Of those FSWs, how many are over the age of 18 years? Of these FSWs, how many have you seen over the past month?” The final response signified participants’ network sizes. RDS weighting used for the adjustment was based on participants’ relative network size and recruitment chains. Population-level estimates were developed using crude sample data that was adjusted to reflect the target population based upon the RDS weights.

Socio-demographic characteristics were scored as single items. Questions included: date of birth, home language (one of South Africa’s 11 official languages or ‘other’), and place of birth (South African provinces or countries with a shared national border). Place of birth was used to indicate whether an individual was from Gauteng or a migrant (national/non-national). They were also questioned on their highest level of education achieved (0 –no schooling; 1 –primary school incomplete; 2 –primary school completed; 3 –high school incomplete; 4 –high school completed; 5 –post school qualification/s, dichotomised into incomplete versus completed post-school qualifications). Participants were also asked about the number of children that they had, categorized as no children, one child, two children, or ≥3 children.

Sexual risk behavior items were scored as single items, and questions included: “How old were you when you first had sex?” (categorized to show legal age of consent), and “For how many years have you been a sex worker?” (categorized as a minimal (1–2 years), moderate (3–7 years) or long (≥8 years) period selling sex). “Does your partner know that you sell sex?” was also asked with a yes/no response set. Questions aimed at estimating the earning potential per sex act in the past day were: “How many of your [regular/one-time clients did you have sex with in the past working day?”, and “How much did you earn the last day that you sold sex?” The amount of money earned was divided by the number of clients seen, and dichotomized at R50.00 earned per sex act.

Condom use was assessed by asking whether they had experienced any of the following during the most recent working day: “condom breaking/bursting”, “condom slipping off”, “condom put on only halfway”, “condom removed during sex” and “no problems, condom worked perfectly". Additional condom-related questions included: “Has there been any occasion in the past month when either a regular or a one-time client went without a condom for any reason when you were having sex?” The response set was ‘yes’, ‘no’ or ‘refuse to answer’. Refuse to answer (n = 2) was assumed to indicate that some inconsistent condom use had existed and the variable was dichotomized into a yes/no. Other questions included: “Over the past year, how often have you experienced the condom breaking or slipping off or only put on halfway, or have you taken it off and continued have sex?” (1 –every time; 2 –often; 3 –sometimes; 4 –never; 5 –condom never used); and “The last time you had sex with a [onetime / regular client, did you use a condom?” (0 –without condom; 1 –male condom; 2 –female condom; 3 –male & female condoms). All condom variables were used to create one variable which reflected any inconsistencies in either condom use or in response sets across the multiple condom variables. This was not a scale, but rather an indication of correctness and consistency of use and responses.

Participants were also asked about sex work-related practices: “In the past month, have you used anything to dry/clean/tighten your vagina before having sex?”; “In the past month, have you used anything to lubricate your vagina during sex?”; and “In the past month have you used anything to hide that you were bleeding/menstruating while having sex?” Response options ranged from 1 (every time) to 4 (never). Participants were given a checklist of items to indicate which they had used for dry sex or to hide menstrual bleeding. Pick-up location was determined by asking: “Where do you normally pick up your clients?” and a checklist of 19 possible pick-up locations was provided, including venues such as brothels, hotels, streets, truck stops, hostels and taverns. Questions were drawn, in part, from an existing sex work surveillance questionnaire [5], the WHO sexual and reproductive health questionnaire [52], but were also based on programmatic evidence indicating the use of specific practices by the community.

AUDIT-C [53] was used to assess binge drinking. This validated scale (∞ 0.891) has been used in studies on FSWs [5]. An additional question to assess the average volume per drink was added: “How big is a typical drink (one drink is a beer/glass of wine, etc.)?” The research team felt that this was important given that the original audit scale did not consider the volume of each drink, and that such large quantities of alcohol are consumed in Sowetan taverns and hostels. In South Africa, for example, a 750 ml beer is considered one drink and is typically sold within taverns and hostels in Soweto. This is three times the volume of a standard beer (250 ml). Responses were, thus, based upon standard drink volumes sold in local drinking establishments as single drinks (0 –no drink; 1–250 ml (small beer or glass of wine); 2–440 ml; 3–500 ml; 4–750 ml (bumpie); 5–1-liter wine, 6–2-liter wine, 7 –a 5-liter (big box wine)). The volume variable was converted into milliliters.

A new drinking variable was created using the AUDIT-C scale, with the additional volume variable included. The original audit scale scores were calculated using the three-item scale with the standard female cut-off at 3. A chi-square test of association was conducted between the audit categories and the volume of alcohol consumed per drink, showing a significant association. A new four-item audit score was then developed which included the volume variable (milliliters consumed). To test this item, we divided our dataset in half and ran an exploratory factor analysis on the first half of the dataset, yielding loadings on one factor. A confirmatory factor analysis on the second half of the data confirmed these loadings, accounting for covariates. A final confirmatory factor analysis was run on the entire dataset. The Cronbach alpha score for our dataset was 0.89 on the new scale as compared with 0.78 using the original scale. This suggested the new scale’s acceptability for use in this study. A cut-off score was determined by considering contextual factors relating to sex work in Soweto and allowing for a realistic estimate of the volume of alcohol that could be consumed infrequently. A score of 6 enabled a person to drink 500 ml of alcohol infrequently. The final scores were dichotomized, with any score ≥6 indicating a high level of binge drinking.

In assessing STI symptoms, participants were asked: “In the past 6 months, have you experienced any of the following symptoms: painful or burning urination; sores/boils around vagina; itching vagina; abnormal vaginal discharge; pain in bottom of stomach unrelated to menstruating/loop; or none of the above?” This was dichotomized as symptoms present versus symptoms absent.

Violence was assessed using the WHO violence against women questionnaire [54], which was adapted to ask about violence specific to various perpetrator types, including IPs. In assessing physical violence, questions included: “Within the past year, did any [partner/client/police hit you with a fist or with something else (such as a beer bottle, stick or belt) which could hurt you?”, and “Within the past year, did any [partner/client/police kick, drag, beat, choke or burn you?” Questions around sexual violence included: “Within the past year, did any [partner/client/police physically force you to have sex when you did not want to?”, and “Within the past year did you have sex with any [partner/client/police when you did not want to because you were afraid of what he might do?” All forms of violence were assessed by perpetrator type before moving onto the next perpetrator type. Questions were scored as 1 –never; 2 –once; 3 –a few times; and 4 –many times.

The following question was drawn from the Childhood Trauma Questionnaire (CTQ): “I had sex with someone who was not my boyfriend because I was threatened or frightened or forced”, with responses ranging from 1 (never) to 4 (very often). Questions also included stigma-related rape: “Within the past year have you experienced sexual abuse because you are a sex worker?” (1 –never to 4 –very often), and forced first sex: “Which of the following statements most closely describes your experiences the first time you had sexual intercourse?” Response choices were: 1 –I was willing|; 2 –I was persuaded; 3 –I was tricked; 4 –I was forced; and 5 –I was raped. A new variable was created with options 3–5 being categorized as sexual assault versus no sexual assault (1–2). All violence questions were dichotomized into no violence versus some violence. For the analysis, two violence variables were generated: IP physical/sexual assault, and a non-IP sexual assault. The original WHO violence against women questions relating to IPs were then used to generate a variable indicating some or no physical/sexual violence by an IP. All other violence questions were used to generate the second violence variable relating to any other sexual violence reported during the study, which included client or police sexual assault, childhood sexual assault, stigma-related rape or forced first intercourse.

In line with WHO Guidelines [55], two rapid assays were conducted to ascertain HIV status (Abon™ and First Response™). An enzyme-linked immunosorbent assay (ELISA) was used on individuals who tested indeterminate (n = 2).

## Statistical analysis

In analyzing RDS data, specialized statistical software (Respondent-Driven Sampling Analysis Tool (RDSAT)[56] was used to assess homophily, convergence, and to produce population-level estimates. The program is freely available for download, along with an operational manual (http://www.respondentdrivensampling.org). As RDSAT does not perform statistical testing, interpretation was based upon adjusted 95% confidence intervals (95% CI). Significance was assumed if there was no overlap between 95% CI. Univariate and bivariate descriptive statistics and frequencies were determined for categorical variables presented by HIV infection (Tables 1 and 2). Female SW behaviors are presented in Table 3 (univariate only). A chi-square test of association was used for bivariate analysis on both adjusted (RDSAT) and unadjusted data (STATA13).

**Table 1:** HIV prevalence and demographic characteristics by HIV status for female soweto-based sex workers, including raw percentages and adjusted population level prevalence with 95% confidence intervals for both univariate and bivariate analysis showing row percentage.

Variable (n = 508)	OVERALL		HIV +		HIV -	
	Raw n (%)	RDS Adjusted % (95% CI)	Raw n (%)	RDS Adjusted % (95% CI)	Raw n (%)	RDS Adjusted % (95% CI)
HIV prevalence						
HIV-infected	280 (55.1)	53.6 (47.5–59.9)	-	-	-	-
HIV-negative	228 (44.9)	46.4 (40.4–52.4)	-	-	-	-
Age						
18–24	124 (24.4)	23.5 (18.5–29.1)	41 (33.1)	**28.8 (19.0–29.0)**	83 (66.9)	**71.2 (61.0–81.1)**
25–29	114 (22.4)	20.9 (16.2–25.7)	57 (50.00)	48.7 (35.0–60.2)	57 (50.0)	51.3 (39.6–64.1)
≥30	270 (53.2)	55.6 (49.2–61.9)	182 (67.4)	**65.6 (57.7–73.7)**	88 (32.5)	**34.4 (26.6–42.1)**
Home language						
Zulu	274 (53.9)	52.3 (46–59.5)	156 (56.9)	54.7 (47.0–63.3)	118 (43.1)	45.3 (36.8–53.7)
Sotho	131 (25.8)	26.7 (20.5–32.5)	70 (53.4)	51.1 (40.2–63.2)	61 (46.6)	48.9 (36.4–60.3)
Other	103 (25.8)	21 (15.9–26.2)	54 (52.4)	54.5 (40.5–67.1)	49 (47.6)	45.5 (33.0–58.8)
Place of birth						
Gauteng	346 (68.1)	71.6 (64.6–77.2)	172 (49.7)	46.9 (40.2–54.1)	174 (50.3)	53.1 (45.5–60.0)
Other (includes cross-border migrants)	162 (31.9)	28.5 (22.7–35.4)	108 (66.7)	**70.6 (60.9–78.6)**	54 (33.3)	**29.4 (21.2–39.0)**
Education						
Incomplete schooling	384 (75.6)	74.2 (69.2–79.9)	234 (60.9)	**60.1 (53.8–66.6)**	150 (39.1)	**39.9 (33.2–46.4)**
Secondary complete/some tertiary	124 (24.4)	25.8 (20.4–30.9)	46 (37.1)	**34.7 (23.2–47.0)**	78 (62.9)	**65.3 (53.1–76.7)**
Number children						
No children	82 (16.1)	13.2 (9.8–17.1)	36 (43.9)	38.4 (25.3–54.3)	46 (56.1)	61.1 (45.8–75.4)
One child	164 (32.3)	32.2 (27.0–37.9)	79 (48.2)	45.6 (36.4–56.8)	85 (51.8)	54.4 (43.1–63.9)
Two children	153 (30.1)	33.5 (28.1–39.0)	93 (60.8)	59.8 (48.2–69.9)	60 (39.2)	40.2 (30.0–51.0)
Three+ children	109 (21.5)	21.1 (16.8–25.6)	72 (66.1)	**64.3 (53.4–75.0)**	37 (33.9)	**35.7 (24.7–46.8)**

**Table 2:** Sexual risk factors characteristics by HIV status for female Soweto based sex workers, including raw percentages and adjusted population level prevalence with 95% confidence intervals for both univariate and bivariate analysis showing row percentage.

Variable (n = 508)	Overall		HIV +		HIV –	
	Raw n (%)	RDS Adjusted % (95% CI)	Raw n (%)	RDS Adjusted % (95% CI)	Raw n (%)	RDS Adjusted % (95% CI)
Age first sex						
4–15 years	157 (30.9)	30.9 (25.9–35.9)	94 (33.6)	58.4 (48.0–68.2)	63 (27.6)	41.6 (32.6–52.0)
16–25 years	351 (69.1)	69.1 (63.9–74.1)	186 (66.4)	51.5 (44.5–58.9)	165 (72.4)	48.5 (41.1–55.5)
Length of time selling sex						
1–2 years	171 (33.7)	38.5 (32.5–45.1)	73 (42.7)	44.1 (34.6–53.3)	98 (57.3)	55.9 (46.7–65.5)
3–7 years	233 (45.9)	41.4 (35.3–46.8)	136 (58.4)	57.8 (48.7–65.3)	97 (41.6)	42.2 (35.1–51.4)
8–33 years	104 (20.5)	20.1 (15.4–25.6)	71 (68.3)	**63.5 (50.1–76.9)**	33 (31.7)	**36.5 (23.1–49.5)**
Partner knowing she sells sex						
Partner does not know	364 (72.4)	80.1 (74.3–84.3)	183 (50.3)	48.8 (42.5–55.8)	181 (49.7)	51.2 (44.4–57.7)
Partner knows	139 (27.6)	19.9 (15.7–25.2)	94 (67.6)	**74.2 (65.4–83.3)**	45 (32.4)	**25.8 (16.8–34.8)**
Number clients in the past day						
0–4	219 (43.1)	43.4 (37.3–48.9)	107 (48.9)	46.1 (37.1–55.9)	112 (51.1)	53.9 (43.7–62.6)
5–19	289 (56.9)	56.6 (50.7–62.5)	173 (59.9)	**59.7 (52.1–66.8)**	116 (40.1)	**40.3 (32.8–48.2)**
Daily earning potential/client						
≤R50.00	350 (68.9)	70.4 (64.6–75.3)	199 (56.9)	55.8 (49.3–63.1)	151 (43.1)	44.2 (37.0–50.8)
>R50.00	158 (31.1)	29.6 (24.8–35.5)	81 (51.3)	48.6 (38.4–59.0)	77 (48.7)	51.4 (40.9–61.6)
Inconsistent & problematic condom use						
Consistent condom usage	122 (24.0)	22.3 (117.9–27.5)	65 (53.3)	53.9 (43.2–65.1)	57 (46.7)	46.1 (34.8–56.7)
Inconsistent condom usage	386 (76.0)	77.7 (72.3–82.2)	215 (55.7)	53.6 (46.4–60.4)	171 (44.3)	46.4 (39.3–53.6)
Dry sex in the past month						
Dry sex reported	129 (25.4)	24.9 (20.4–29.4)	198 (52.2)	**67.7 (56.1–76.5)**	181 (47.8)	**32.3 (23.0–44.5)**
No dry sex reported	379 (74.6)	75.1 (70.7–79.8)	82 (63.6)	48.9 (42.3–56.3)	47 (36.4)	51.1 (43.5–57.6)
STI symptoms in the past 6 months						
STI symptoms	259 (51.0)	49.8 (43.9–55.7)	157 (60.6)	**61.2 (53.1–68.9)**	102 (39.4)	38.8 (30.5–46.7)
No STI symptoms	249 (49.0)	50.2 (44.3–56.1)	123 (49.4)	46.1 (37.7–54.6)	126 (50.6)	53.9 (46.1–62.2)
Most popular pick-up location						
Hostel used as a pick-up location	306 (60.2)	52.2 (45.8–59.6)	178 (58.2)	55.7 (47.2–63.6)	128 (41.8)	44.3 (36.6–53.0)
Other locations used	202 (39.8)	48.0 (40.4–54.5)	102 (50.5)	51.8 (43.2–61.2)	100 (49.5)	48.2 (38.8–56.5)
Binge drinking (including volume per drink)						
Low-level drinking	127 (25%)	23.8 (19.1–28.6)	64 (22.9)	21.8 (15.4–28.5)	63 (27.6)	26.7 (19.6–35.5)
High-level drinking	381 (75%)	76.2 (71.2–81.2)	216 (77.1)	78.2 (71.8–84.7)	165 (72.4)	73.3 (64.1–80.6)
IP physical/sexual violence ever						
None	220 (43.3)	46.2 (40.4–51.9)	123 (55.9)	56.7 (48.3–64.9)	97 (44.1)	43.3 (34.7–51.9)
Some	288 (56.7)	53.8 (48.1–59.6)	157 (54.5)	50.8 (42.8–58.5)	131 (45.5)	49.2 (40.9–57.5)
Sexual violence by non–IP						
None	231 (45.5)	44.5(38.6–49.8)	112(48.5)	46.2(37.1–54.2)	119(51.5)	53.8(45.4–63.0)
Some	277 (54.5)	55.5(50.2–61.4)	168(60.7)	**59.6(52.4–67.5)**	109(39.4)	**40.4(33.1–48.0)**

Significant items are in bold

**Table 3:** Univariate description of female sex work practices and behaviors not included in bivariate or multivariate analyses, including raw percentages and adjusted population level prevalence with 95% confidence intervals.

Variable (n = 508)	Raw n(%)	RDS Adjusted % (95% CI)
Circumstances behind entry into SW		
No income food/shelter/kicked out	144 (28.4)	28.6 (22.4–33.4)
Nowhere to stay/orphan/widow/deserted	49 (9.7)	9.1 (5.9–12.5)
Family income	211 (41.54)	39.4 (35.0–47.3)
Teenage pregnancy	14 (2.8)	3.6 (1.5–6.0)
Fast money/personal expenses	62 (12.2)	13.0 (9.1–16.4)
Other	28 (5.5)	6.3 (3.4–9.4)
Venues within which FSWs sell sex in Soweto *		
Taverns	416 (81.9)	85.6 (81.2–89.3)
Hostels	306 (60.2)	52.0 (45.5–59.4)
Brothels	77 (15.2)	14.2 (10.4–18.1)
Hotels	35 (6.9)	6.6 (4.2–9.8)
Street	49 (9.7)	8.4 (5.5–11.5)
Most popular service		
Vaginal sex	453 (89.2)	89.5 (85.9–93.0)
Anal sex	35 (6.9)	6.6 (4.0–9.5)
Oral sex	14 (2.8)	2.9 (1.2–4.9)
Other (dinner, massage, phone, hand-job, stripping, other)	6 (1.2)	0.3 (0.0–0.6)
Lubricant used during sex*	247 (48.6)	47.3 (41.1–53.5)
Lubricant	225 (44.3)	43.2 (37.7–49.4)
Saliva	15 (3.0)	2.9 (1.5–4.6)
Vaseline	20 (3.9)	3.8 (1.7–6.2)
Baby oil	30 (5.9)	4.1 (2.4–6.3)
Hiding menstrual blood while selling sex *	182 (35.8)	32.1 (27.2–37.8)
Tampon	17 (3.4)	3.2 (1.4–5.6)
Kitchen sponge	59 (11.6)	10.4 (7.1–13.9)
Cotton wool	67 (13.2)	11.6 (8.0–15.3)
Red colored condom	81 (15.9)	14.3 (10.7–18.3)

* multiple responses permitted

For inclusion within the multivariate model, variables needed to be significant at a bivariate level on interpretation of the 95% CI, or suggested by the literature to be relevant to SW HIV vulnerability. RDSAT is currently limited in its ability to perform multivariate analysis. A second package, Respondent-Driven Sampling Analyst (RDS-Analyst) [57] was used to export equivalent RDS weights using the RDS-II function for the multivariate analysis which was performed in STATA13. Extensive sensitivity analyses were conducted at a multivariate level using mixed effects logistic regression models to control for potential clustering, which may have resulted from RDS recruitment. This was done using the svyset command. Originating seed, wave of recruitment and residential suburb were specified as random effects, and no severe clustering effects were found. Therefore, the logit command was used to perform the multivariate analysis (weighted and unweighted). Multiple logistic models were developed for both adjusted and unadjusted factors associated with HIV seropositive status. Both forward and backward stepwise elimination were used manually to develop the final models (Table 4), in which variables were retained at p = ≥ 0.5. In addition to the final variables presented in the models, variables considered for inclusion within the models included consistent and correct condom use, dry sex, duration of involvement in sex work, IP condom use, pick-up venue, binge drinking, IP violence and any sexual violence by a non-IP. Minor differences existed between models. Five observations were dropped from the multivariate analyses due to missing responses relating to a partner knowing that sex was being sold. This was the only missing data.

**Table 4:** Logistic regression analysis of factors affecting HIV seropositive status of female sex workers in a South African township, with both weighted and unweighted multivariate analysis displayed.

Variable (n = 502)	Unweighted AOR (95% CI)	P-value	Weighted AOR (95% CI)	P-value
Age (categorized along IQR)				
18–25	**(ref)**	**-**	**(ref)**	**-**
26–30	**2.2 (1.3–3.9)**	**0.005**	**2.9 (1.3–6.3)**	**0.008**
31+	**4.2 (2.6–6.8)**	**0.0001**	**4.9 (2.6–9.3)**	**0.0001**
Place of birth				
Gauteng	**(ref)**	**-**	**(ref)**	**-**
Other (includes cross-border migrants)	**1.7 (1.1–2.6)**	**0.018**	**2.3 (1.3–4.0)**	**0.003**
Education				
Incomplete secondary schooling	**2.7 (1.7–4.3)**	**0.0001**	**2.8 (1.6–5.0)**	**0.0001**
Secondary completed /some tertiary	**(ref)**	**-**	**(ref)**	**-**
Partner knows she sells sex				
Partner does not know	**(ref)**	**-**	**(ref)**	**-**
Partner knows	**1.7 (1.0–2.7)**	**0.031**	**2.2 (1.3–3.8)**	**0.005**
No. of clients in the past day				
0–4	**(ref)**	**-**	**(ref)**	**-**
5–19	**1.7 (1.2–2.6)**	**0.005**	**1.9 (1.1–3.2)**	**0.02**

## Results

In total, 508 FSWs were enrolled in the study over seven months during 2016 (Fig 2). All but two participants were black African, with 16 cross-border immigrants. Recruitment chains progressed up to a maximum of 25, with two seeds highly productive (10–25 waves), four seeds minimally productive (4–8 waves), and five non-productive seeds (Fig 1). Equilibrium, homophily and convergence were achieved on all key variables examined, including HIV status, age, education and drinking.

Participants ranged in age from 18–59 years, with 24.4% 18–24 years of age, 20.9% 25–29 years, and 55.6% 30 years or older. Slightly more than half were Zulu speaking (52.3%) and 71.6% originated from the Gauteng province, where Soweto is located. Only 25.8% had completed their secondary schooling or done some tertiary level schooling. A small proportion of FSWs did not have any children (13.2%), while 32.2% had one child, 33.5% had two children and 21.1% had ≥3 children. HIV infection was prevalent in 28.8% of those aged 18–24 years, as compared with 48.7% among 25–29 year olds, and 65.6% among those 30 years and older. There was no difference in HIV prevalence between different home languages. Of the FSWs that came from outside Gauteng, 70.6% were HIV-positive, as compared with 46.9% from Gauteng. More than half of those with an incomplete education were HIV-infected (60.1%). Of FSWs who had three children or more, 64.3% were HIV-positive (Table 1).

Most reported their first sexual experience between the ages of 16 and 25 (69.1%). Thirty-eight percent reported selling sex for ≤2 years, 41.4% for 3–7 years and 20.1% for ≥8 years. Only one in five FSWs had told their primary partner that they sold sex (19.9%). The number of clients in the previous working day ranged from 0–19, with 56.6% reporting sex sold to 5–19 clients. Almost three quarters (70.4%) earned ≤ R50.00 (≤ $4.00) per client on their last working day. Correct and consistent condom use was reported 22.3% of the time. Dry sex practices were reported by one in four women (24.9%). Almost half the FSWs had reported STI symptoms in the past six-months (49.8%), and 52.2% used a hostel as a pick-up location. High levels of binge drinking were reported in 76.2% of the respondents. Intimate partner violence was reported at 53.8%, while sexual violence by a non-IP was reported at 55.5%. Age at first sex was not significantly associated with HIV status. Most FSWs who had told their partner that they sold sex were HIV-infected (74.2%). Almost 60% of sex workers who had more than five clients per day were HIV-positive. Neither earning potential per client nor condom use differed according to HIV status. However, 67.7% of those reporting dry sex and 61.2% of those reporting STI symptoms were HIV-positive. Selling sex in a hostel was not significantly associated with HIV, and HIV was not more common among women with high levels of binge drinking, nor among those exposed to IP violence. However, 59.6% of women who reported non-IP sexual assault were HIV-positive (Table 2).

Reasons for sex work included: supplementing family income (39.4%), no income for food/shelter, or having been kicked out of their homes (28.6%). Sex was usually sold within taverns and hostels (85.6% and 52.0%, respectively) with less than 20% reporting selling sex in a brothel, hotel or on the street. Vaginal sex was the most popular service procured by clients (89.5%). Overall, anal sex was sold by 22.0% of FSWs and 70.7% of those engaging in anal sex reported consistent condom use by clients (not shown in table). Lubricant use was reported by 47.3%, with actual lubricant the most popular agent (43.2%). Hiding menstrual bleeding was practiced by 32.1% of FSWs (Table 3).

As shown in Table 4, for the adjusted analysis, age was significantly associated with HIV infection, with those aged 26–30, and >31 having a 2.9 and 4.9 times greater likelihood of being HIV-infected, respectively (95% CI 1.3–6.3, p = 0.008, and 95% CI 2.6–9.3, p = 0.0001, respectively) compared to those aged 18–24 years. Respondents born outside of Gauteng were at greater likelihood of HIV infection (AOR 2.3, 95% CI 1.3–4.0, p = 0.003). Women who had not completed secondary school were more likely to be HIV-infected than those who had completed their schooling (AOR 2.8, 95% CI 1.6–5.0, p = 0.0001). A partner’s knowledge that a woman was selling sex was significantly associated with HIV infection (AOR 2.2, 95% CI 1.3–3.8, p = 0.005) as was having more than five clients (AOR 1.9, 95% CI 1.1–3.2, p = 0.02). Violence was non-significant in either model.

## Discussion

We found immense vulnerability among FSWs to HIV, with 53.6% infected overall. Our data also highlights the high burden of violence borne by this population. Structural factors, including the lack of completed education, and migrancy were strongly associated with HIV. Other factors strongly associated with HIV were having more than five clients a day and/or a partner knowing that sex was sold. Age was also a an associated factor. Contrary to expectations, inconsistent condom use and binge drinking were not significantly associated with HIV status.

Our findings add to a growing body of evidence highlighting that many FSWs in South Africa have a substantially higher burden of disease than the pooled prevalence of 36.9% documented across sub-Saharan Africa, or the 11.8% reported in 50 countries in 2012 [2]. The findings suggest that FSWs in Soweto have an HIV prevalence substantially higher than the South African national average, and of women aged 25 years and older [8]. Prevalence among FSWs in Soweto is also higher than previously recorded among women who have engaged in transactional sex in the township (44.8%) [23]. However, the prevalence in our study was lower than that documented among FSWs in the Johannesburg site of the SAHMS, where prevalence was reported at 73.1% [5]. The Johannesburg study included a large inner-city immigrant population (31.5%) [5]. In comparison, only 16 FSWs in our study reported having immigrated from another country. This may have been an aggravating factor in the higher prevalence rates reported for Johannesburg, given that 70.6% of migrant FSWs were HIV-infected. Despite the few cross-border migrants, our study confirms the need for interventions geared towards migrant SWs. It further provides evidence of the need for structural level changes to laws and policies which perpetuate HIV vulnerability among FSWs [1].

The importance of education was shown as a key driving factor in the vulnerability of FSWs to HIV. South Africa has a checkered socio-political history, which has led to poor educational outcomes for many women [44, 58]. We found that 75% of FSWs reported incomplete education, compared with 58% in the general population [59]. Poor educational outcomes [44, 58] have been found to fuel an informal sex-for-money economy [1, 44], which increases the likelihood of HIV. South Africa has an unemployment rate of 27.1% [60], and people with secondary school qualifications have a distinct advantage over their uneducated counterparts in obtaining employment within the formal economy. In our view, the overall low levels of education found in our study contribute in multiple ways to increased vulnerability to HIV and economic marginalization.

Our findings highlight the vulnerability of women over 25 years, and the substantial burden of disease among women over 30 years, two thirds of whom were HIV-infected. Research has shown HIV prevalence peaking in FSWs in Cape Town and Durban for women of a similar age [5]. By comparison, HIV prevalence in Johannesburg was previously found to peak in sex workers aged 16–24 years [5]. One study, which included a small convenience sample of FSWs, found that the risk of HIV decreased with age [7]. Our study, however, suggests that interventions geared toward HIV prevention among SWs <30 years is vital in addressing FSW vulnerability to HIV acquisition. It further highlights the need for strong HIV treatment programs for women >30 years in Soweto.

We have found very high levels of violence among FSWs in South Africa. These findings are consistent with those of the SAHMS, in which half the FSWs reported physical violence, compared with 53.8% in the present study. However, more than twice as many women reported sexual assault in our study as the number of assaults reported in the SAHMS (55.5% versus 21.9%, respectively) [5]. Our prevalence of IPV is almost double that reported by women in the general population in Gauteng (25.3%), but three times that of women reporting IPV (18%) [61]. This study adds to a growing body of literature emphasizing the high levels of violence in South Africa and, in particular, those to which SWs are exposed [1, 5, 9, 14, 21–23, 29, 43]. Despite violence having reached epidemic proportions, South Africa has no national strategy to prevent violence against women. This situation is compounded by the continued criminalization of sex work, which hampers SWs’ ability to report rape and assault, and perpetuates discrimination, which often incites violence against SWs.

Correct and consistent condom use in the past month was not associated with HIV-infection in this study. While consistent condom use is effective in preventing HIV infection [9, 62], it is unlikely that these FSWs became infected within this timeframe. This null finding may indicate that FSWs are less likely to protect themselves once they discover an HIV-positive status. However, future research is required to elaborate more substantially on this possible explanation for poor condom use. Our findings suggest that condom use at last sex may be both an inflated and inadequate measure of consistent condom use. Shai et al[63] highlight the importance of multiple measures of condom use to develop an adequate understanding of consistency over time. Using a similar measure, we observed a decline in consistency between condom use at last sex (85%) and our measure of correct and consistent condom use (22.3%). While recent trends have indicated that SWs consistently use condoms with their clients [5, 30], our findings suggest that a new measure of condom consistency is required. Furthermore, the extremely low correct and consistent condom use reported in the Soweto environment is alarming, particularly given the mass promotion of condoms as a safe sex mechanism. In this environment, usage may be influenced by other factors such as exposure to violence [23, 32], which has been reported to be high across the township [64]. Further research is required to understand mechanisms inhibiting the uptake of consistent condom use.

Approximately three quarters of respondents had not told their partners that they engaged in sex work. Three quarters of those who had disclosed their profession were HIV-infected, more than half of whom reported condom use with their IP, possibly to protect him. It is likely that an IP’s knowledge of a SW’s profession stems from the IP being a former client. Thus, knowledge of her vocation may be mediating the relationship between other partner related factors and HIV vulnerability. This requires further research to understand what partner factors relating to knowing that sex is sold, play a role in HIV vulnerability.

This study describes a previously undocumented subset of FSWs who are based within Soweto, an urban African township on the outskirts of Johannesburg, Gauteng. Contrary to former accounts in the literature [9–11], FSWs in Soweto work largely in taverns or informal drinking establishments, and hostels. While transactional-sex relationships in Soweto have been described previously [13, 14], our study highlights the impoverished nature of FSWs in Soweto, many of whom enter the sex work industry as a means of survival. Dry sex or douching was bivariably associated with HIV, and may indicate the increased vaginal cleaning practices associated with more common vaginal infections for HIV-positive women[38]. By contrast, FSWs in Kenya were twice as likely to acquire HIV if they douched with water and almost four-times the likelihood of HIV from douching with soap [39]. The use of lubricant and the practice of hiding menstrual blood by inserting an object into the vagina have not been well documented in the literature. Hiding menstrual blood has been anecdotally documented by local SW organizations. The practice involves the insertion of objects such as a kitchen sponge, cotton wool, a cloth, or tampon into the vaginal canal to prevent blood from showing during and after the sale of sexual services. In a sub-analysis, hiding menstrual blood was found to be associated both with reporting recent STI symptoms and client violence (analysis not shown). STI symptoms were associated with increased vulnerability to HIV. This suggests that it is vital that SW-specific practices need to be better documented and understood to ensure that interventions can be developed which are both appropriate to the FSW community and target their vulnerability to HIV.

Our study had several limitations including insufficient information on the temporal sequencing of HIV seroconversion and associated factors such as migrancy, entry into sex work, condom use and alcohol consumption. Implicit in the analysis is the assumption that HIV seroconversion followed these associated factors, but we are unable to confirm that this is the case. Our analysis assumed that recent sexual behavior is indicative of past sexual behaviors, but caution should be taken in interpreting these results. Some variation was observed between adjusted and unadjusted bivariate analysis in the RDS analysis; understanding of the use of RDS weights in multivariate analysis is limited [65, 66]. However, the difference between adjusted and unadjusted analyses suggests that when used in conjunction with sensitivity tests, RDS data provide an alternative model for use in weighted multivariate logistic analyses. The RDS strategy was found to be a useful recruiting mechanism to reach FSWs in Soweto. However, care must be taken in screening potential participants to ensure that they do sell sex. Importantly, it is vital that more complex analyses are conducted on SW data to unpack the complex interplay of causal mechanisms leading to their vulnerability to HIV, which are likely to be non-linear in nature.

HIV prevalence among FSWs in South Africa is excessively high and requires urgent action to arrest the unabated growth of the disease. Our findings highlight the vulnerability of FSWs to the disease, which is strongly driven by a combination of structural, biological and behavioral determinants. Soweto represents a microcosm of South Africa in terms of the epidemic of violence and HIV plaguing society, and which is often beyond the control of individuals. The South African government needs to urgently address structural factors impacting entry into and practices used in sex work. These factors often function in highly complex ways, and require innovative and alternative thinking beyond the scope of traditional HIV prevention and treatment. This would ensure that SWs are empowered to protect themselves effectively against HIV. This study also highlights the need for improved access to ART services as both a treatment and prevention strategy. At the same time, our findings suggest that there may be more immediate concerns facing FSWs which require more urgent attention. In line with Shannon et al [1, 67], these individual and structural level factors function to drive the epidemic and require inclusion within SW HIV programs. In the case of SWs, capacity development in the form of adult education, coping skills and mindfulness practices may prove valuable tools in the HIV armory. Furthermore, in comparison to the SAHMS findings [5], these findings highlight the critical value of profiling a given population to ensure that interventions are contextually tailored. Such action will ensure that access to treatment and prevention through rights-based services is effectively implemented in South Africa in line with the *South African National Sex Worker HIV Plan 2016–2020* [3].

## Supporting information

S1 Text RDS assumptions plots. (PDF)

S2 Text Questionnaire (English). (PDF)

S3 Text Questionnaire (All Languages). (PDF)
