# Supplementary material for: Cross-sectional study of female sex workers in Soweto, South Africa: Factors associated with HIV infection
Source: PLoS One. 2017 Oct 5;12(10):e0184775. doi: 10.1371/journal.pone.0184775 (PMC5628807; doi:10.1371/journal.pone.0184775)
Supplement: S1 Text — (PDF) [file pone.0184775.s001.pdf]

S1 Text. RDS Assumptions (Pdf)

Please see below RDSAT exported assumptions for homophily, convergence and bottleneck plots. These are supplied in evidence that all assumptions were met in relation to the RDS methodology.

SAMPLE & POPULATION LEVEL HOMOPHILY

SAMPLE HOMOPHILY

HIV STATUS
Homophily = **1.06**

INTIMATE PARTNER VIOLENCE
Homophily = **0.99**

NON INTIMATE PARTNER SEXUAL ASSAULT
Homophily = **0.98**

POPULATION LEVEL HOMOPHILY

HIV STATUS

negative	positive
**1.06**	**1.07**

INTIMATE PARTNER VIOLENCE

None	some
**0.99**	**1.01**

NON INTIMATE PARTNER SEXUAL ASSAULT

NONE	SOME
**0.97**	**0.97**

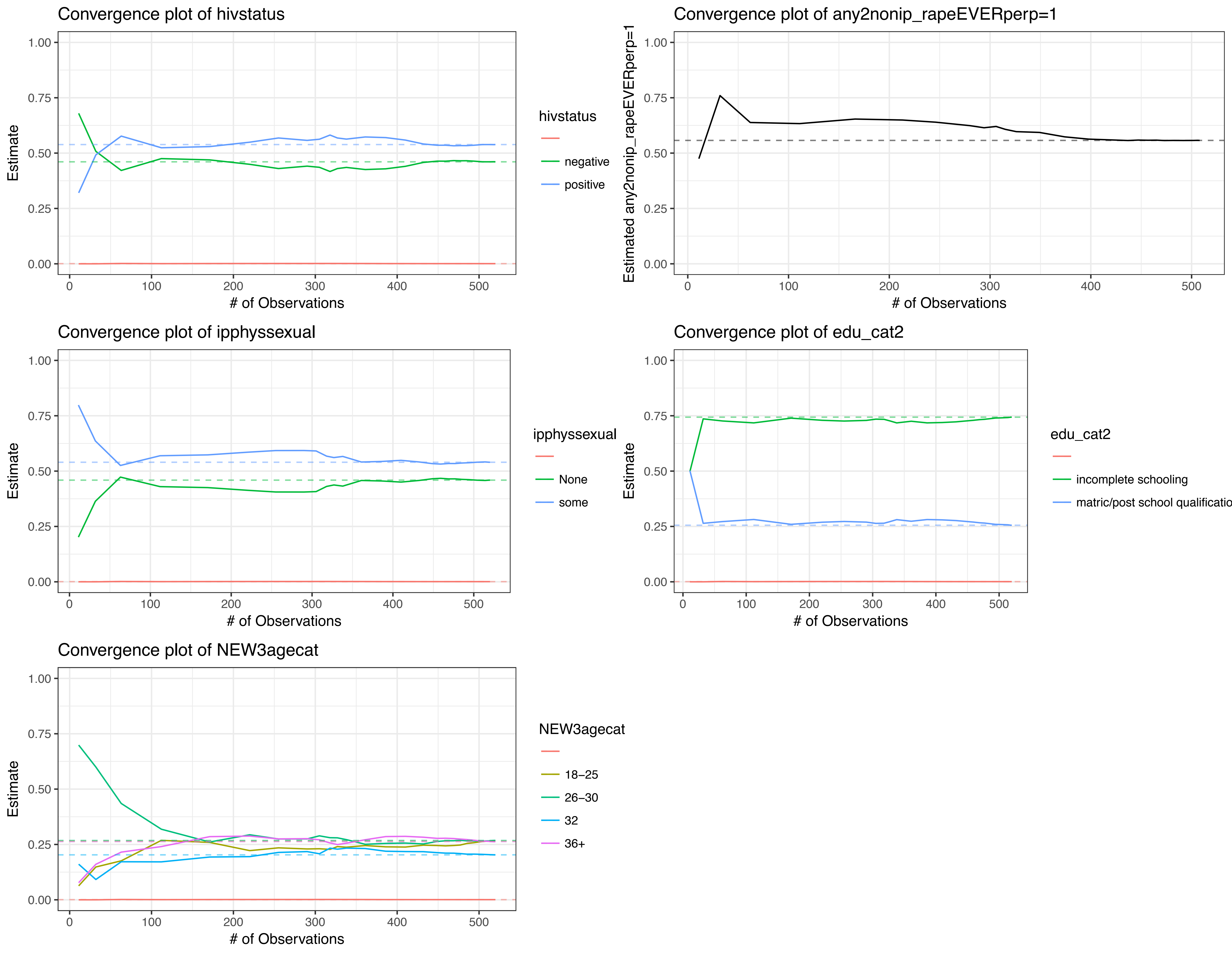
Convergence plot of hivstatus
Estimate
# of Observations
hivstatus
negative
positive
Convergence plot of any2nonip_rapeEVERperp=1
Estimated any2nonip_rapeEVERperp=1
# of Observations
Convergence plot of ipphyssexual
Estimate
# of Observations
ipphyssexual
None
some
Convergence plot of edu_cat2
Estimate
# of Observations
edu_cat2
incomplete schooling
matric/post school qualificatio
Convergence plot of NEW3agecat
Estimate
# of Observations
NEW3agecat
18–25
26–30
32
36+
0.00
0.25
0.50
0.75
1.00
0
100
200
300
400
500

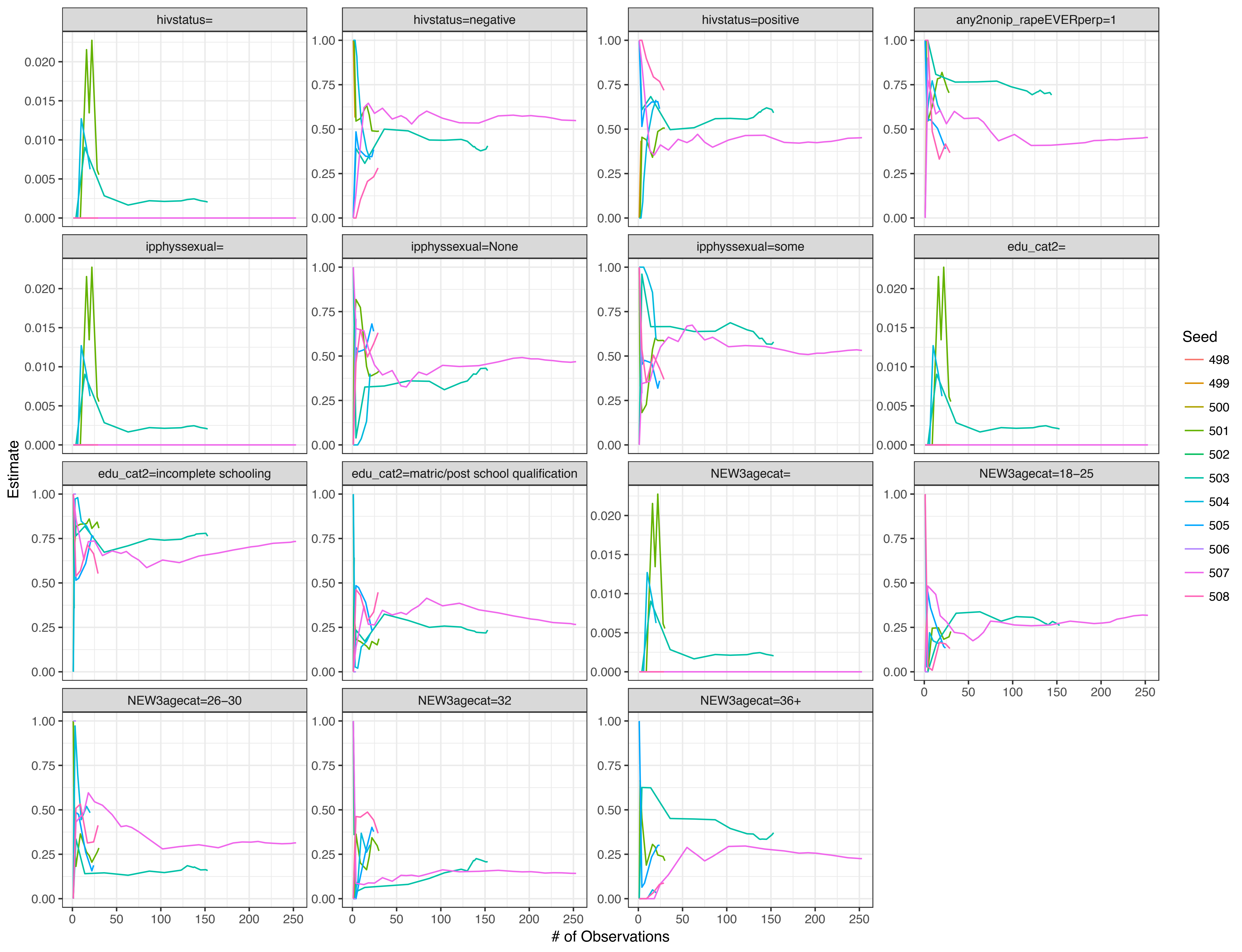
hivstatus=
hivstatus=negative
hivstatus=positive
any2nonip_rapeEVERperp=1
ipphyssexual=
ipphyssexual=None
ipphyssexual=some
edu_cat2=
edu_cat2=incomplete schooling
edu_cat2=matric/post school qualification
NEW3agecat=
NEW3agecat=18–25
NEW3agecat=26–30
NEW3agecat=32
NEW3agecat=36+
Estimate
# of Observations
Seed
498
499
500
501
502
503
504
505
506
507
508
