# Supplementary material for: Cross-sectional study of female sex workers in Soweto, South Africa: Factors associated with HIV infection
Source: PLoS One. 2017 Oct 5;12(10):e0184775. doi: 10.1371/journal.pone.0184775 (PMC5628807; doi:10.1371/journal.pone.0184775)
Supplement: S2 Text — (PDF) [file pone.0184775.s002.pdf]

S2 Text. Questionnaire (English) (Pdf)

SCREENING TOOL

Coupon Number

____

Date of Screening

____

Have you scanned their fingerprint into the system? If so, are they a new participant?
(PLEASE DO NOT CONTINUE UNTIL THIS HAS BEEN DONE TO AVOID DUPLICATION INTO THE STUDY)

◯ Yes ◯ No

SCREENING FOR INCLUSION & EXCLUSION CRITERIA

Biological Gender (what you were born)

◯ Female ◯ Male ◯ Other

Self-identifies as a SW or self-identifies as working within a SW hotspot and consensually trading in sex for money or goods as her a primary source of income

◯ Yes ◯ No

Working (as a sex worker) in Soweto

◯ Yes ◯ No

Date of Birth

____
(IF DATE OF BIRTH UNKNOWN, ENTER AGE INTO S12, BELOW)

Age (if DoB unknown)

____

HIV status known or unknown

◯ Known ◯ Unknown

Current victim of human trafficking or sexual exploitation
(ANY INDIVIDUAL WHO REPORTS BEING A CURRENT VICTIM OF HUMAN TRAFFICKING OR EXPLOITATION MUST BE REFERRED TO THE DEPARTMENT OF SOCIAL DEVELOPMENT IMMEDIATELY.)

◯ Yes ◯ No

Are you part of the study team or did you participated in the pilot study for this study?

◯ Yes ◯ No

Do you believe this person is a sex worker

◯ yes, no secondary screening required ◯ yes, only after secondary screening ◯ no, after secondary screening. Please change them to a non sex worker above and do not enroll

SUCCESSFULLY SCREENED! :)Please enroll this person

Participant is NOT ELIGIBLEPlease do not enroll this person

If you were to be enrolled into this study, what language would you prefer to do the survey in?

◯ English ◯ Zulu ◯ Sotho

How many female sex workers do you know in Soweto? By 'know' I mean, people you know by name and would greet if you saw them, and they would greet you back

____
(YOU CAN TAKE YOUR TIME, THINK OF ALL THE FEMALE SEX WORKERS WHO ARE OVER THE AGE OF 18 YEARS AND WHO WORK IN SOWETO. THEY DO NOT HAVE TO WORK HERE EVERY DAY OF THE WEEK, BUT THEY DO NEED TO DO SELL SEX IN SOWETO SOMETIMES.)

Of those female sex workers, about how many are over the age of 18 years?

____
(THE NUMBER CANNOT EXCEED THE TOTAL NUMBER OF FEMALE SEX WORKERS KNOWN)

Of those female sex workers that you know, how many have you seen over the past month?

____
(THE NUMBER CANNOT EXCEED THE TOTAL NUMBER OF FEMALE SEX WORKERS KNOWN)

If you think about the the whole of Soweto, how many female sex workers do you estimate work across the whole of Soweto?

____
(TAKE HER TIME, ASSIST HER TO THINK OF THE WHOLE OF SOWETO, ALL 40 SUBURBS)

What are the main areas that you do business (sell sex) in Soweto?

- [ ] Braamfischerville
- [ ] Tshiawelo
- [ ] Devland
- [ ] Diepkloof
- [ ] Dlamini
- [ ] Dobsonville
- [ ] Doornkop
- [ ] Dube
- [ ] Embuzini
- [ ] Emdeni
- [ ] Freedom Park
- [ ] Greenvillage
- [ ] Jabavu
- [ ] Jabulani
- [ ] Klipspruit
- [ ] Kliptown
- [ ] Lakeside
- [ ] Mapetla
- [ ] Meadowlands
- [ ] Mmesi Park
- [ ] Mofolo
- [ ] Molapo
- [ ] Moletsane
- [ ] Moroka
- [ ] Mzimhlope
- [ ] Naledi
- [ ] Noordgesig
- [ ] Nomzamo Park
- [ ] Nancefield
- [ ] Orlando
- [ ] Phefeni
- [ ] Phiri
- [ ] Pimville
- [ ] Power Park
- [ ] Protea Glen
- [ ] Protea North
- [ ] Protea South
- [ ] Senaorane
- [ ] Tladi
- [ ] Zola
- [ ] Zondi
- [ ] White City
- [ ] Other Soweto
- [ ] Other outside Soweto

Now think about the main area that you work in in Soweto, how many female sex workers do you think work in just that part of the township, in that suburb (so not just the tavern or hostel)? ______________________________

Preliminary

PRELIMINARY INFORMATION

Consented to both questionnaire and HIV tests

◯ Refused to Participate in Questionnaire and HIV Tests ◯ Agreed to Both Survey and HIV Tests
◯ Has Already Participated in The Study

Date and time of interview

____

Have you ever been to the SSWP or met with one of our peer educators prior to today?

☐ No I have not met a PE of been to The Wellness Center
☐ I have met a Peer Educator On Outreach
☐ I have been to The Wellness Center
☐ I have come to an SSWP creative space

What sex worker programmes in and around Soweto, or anywhere else in South Africa have you heard about? (PLEASE SELECT ALL THAT APPLY)

☐ No ☐ Sswp ☐ Sweat ☐ Sisonke ☐ Esselen Street/Wrhi ☐ Red Umbrella Project
☐ Another Sw Project

First name

____

Surname

____

Primary contact phone number

____

Demographic

QUESTIONNAIRE

Age

____

What suburb do you live in? By live in I mean, where do you normally sleep, for at least 3 nights/days of the week?

○ Braamfischerville ○ Tshiawelo ○ Devland ○ Diepkloof ○ Dlamini ○ Dobsonville
○ Doornkop ○ Dube ○ Embuzini ○ Emdeni ○ Freedom Park ○ Greenvillage
○ Jabavu ○ Jabulani ○ Klipspruit ○ Kliptown ○ Lakeside ○ Mapetla ○ Meadowlands
○ Mmesi Park ○ Mofolo ○ Molapo ○ Moletsane ○ Moroka ○ Mzimhlope ○ Naledi
○ Noordgesig ○ Nomzamo Park ○ Nancefield ○ Orlando ○ Phefeni ○ Phiri ○ Pimville
○ Power Park ○ Protea Glen ○ Protea North ○ Protea South ○ Senaorane ○ Tladi
○ Zola ○ Zondi ○ White City ○ Other Soweto ○ Other outside Soweto

Are you sexually attracted to women or men or both or don't know

○ Women ○ Men ○ Both men and women ○ Don't know

What racial group do you belong to?

○ Black African ○ Coloured ○ White ○ Indian/Asian ○ Other

Where were you born?

○ Gauteng ○ Kwazulu Natal ○ Western Cape ○ Eastern Cape ○ Norther Cape
○ Freestate ○ Mpumalanga ○ Limpopo ○ Zimbabwe ○ Mozambique ○ Malawi
○ Botswana ○ Uganda ○ Other

When did you arrive in South Africa?

____

When did you come to live in Soweto?

____
(IF BORN IN SOWETO, ENTER 01-01-[YEAR OF BIRTH])

What is your home language?

○ Zulu ○ Ndebele ○ Xhosa ○ Northern Sotho ○ Sesotho ○ Setswana ○ Siswati
○ Tshivenda ○ Xitsonga ○ English ○ Afrikaans ○ Other

Did you ever go to school? If so, what is the highest grade you have ever successfully achieved (passed)?

○ No Schooling ○ Primary School Incomplete ○ Primary School Completed ○ High School Incomplete
○ High School Completed ○ Post School Qualification ○

Are you currently studying?

○ No ○ Yes

Have either of your parents died?
(IF MOTHER, ANSWER A12IF NEITHER OR DO NOT KNOW SKIP TO A13)

○ Neither ○ Mother ○ Father ○ Mother and Father ○ I Do Not Know

How old were you when your mother died?

____

How many living children do you have?

____
(IF NO CHILDREN, PLEASE ENTER IN 0. IF STILL PREGNANT WITH FIRST CHILD, PLEASE ENTER IN 0IF NO CHILDREN, SKIP TO A16)

Are any of your children under the age of 12?

◯ None Of Them ◯ 1 Child Is ◯ 2 Children Are ◯ 3 Children Are ◯ 4 Children Are
◯ 5 Children Are ◯ All Of Them Are Under 7 Years Of Age

Do any of your children currently live with you?

◯ No ◯ Yes

How many people including yourself live in your house (the place where you stay)?

____

How many rooms are in the house that you live in?

____

Do the people in your home go without food often, sometimes, seldom or never?

◯ Often ◯ Sometimes ◯ Seldom ◯ Never

Do the people in your home eat meat often, sometimes, seldom or never?

◯ Often ◯ Sometimes ◯ Seldom ◯ Never

If a person in your home became very ill and R100 was needed for the treatment or medicine, would you say that to find the money would be very easy, easy, quite difficult or very difficult?

◯ Very Easy ◯ Easy ◯ Quite Difficult ◯ Very Difficult

In the past month, how many people have relied on your earnings/money for food, shelter or school fees?

____
(PROBE ABOUT CHILDREN, PARENTS, SIBLINGS, PARTNER, FLAT MATES OR FRIENDS OR OTHER FAMILY REQUIRING SUPPORT. HELP THEM COUNT BY SHOWING FINGERS!)

Do you have any other activities other than sex work to support your livelihood?

Selling sex is my primary source of income

◯ No ◯ yes

I am employed by a company or person

◯ No ◯ yes

I am unemployed

◯ No ◯ yes

I receive a government grant such as a pension or child support grant

◯ No ◯ yes

I sell/trade goods such as vegetables or clothes to earn money

◯ No ◯ yes

In the last week, where do you normally sleep?

◯ Flat ◯ Hostel ◯ Tavern Back Room ◯ At Home With My Family ◯ A Private House With Other Sex Workers ◯ A Private House With No Other Sex Workers Live With Me ◯ A Brothel ◯ Abandonned Building ◯ On The Streets/Nowhere in Particular, Constantly On The Move ◯ Hotel ◯ Other

What is the main reason that you became a sex worker? Select the one which was most important to you at the time of your decision to become a sex worker

☐ Chosen Work ☐ No Other Jobs Available ☐ to Pay For Drugs/Alcohol ☐ I Needed Money
☐ Forced Into Sex Work ☐ Friend/Relative Who Was A Sex Worker Showed Me ☐ To be independent
☐ Other reason ☐

How many years have you been a sex worker for?

____
(ROUND TO THE NEAREST YEAR)

How old were you when you first sold sex?

____

Under what circumstances did you first sell sex?

◯ No Income to Buy Food Or For Shelter/Kicked Out ◯ Nowhere to stay, have nothing From Being Deserted, Becoming An Orphan Or A Widow/Divorcee ◯ to Add Onto The Family Income ◯ to Pay For Medical Costs in The Family Or For Self ◯ to Earn Fast Money Or Meet Personal Expenses ◯ Tricked Into Going to A Brothel
◯ Forced By Family ◯ Forced By Boyfriend ◯ Had to Pay Back Someone Through Sexual Favours
◯ Forced By Stranger ◯ Forced By friend(s) ◯ Teenage Pregnancy ◯ Other

Where do you normally pick up your mcimbi?
(PLEASE SELECT ALL THAT APPLY)

☐ Tavern ☐ Taxi Rank ☐ Truck Stop ☐ Prison ☐ Referral ☐ Street ☐ Hostel
☐ Hostel Taverns ☐ Internet ☐ Newpaper/Magazine Advertisements ☐ Brothel ☐ Strip/Dance Club
☐ Police ☐ Security ☐ Salon(Hair) ☐ Pick-Up (Road/Street Corner) ☐ Mine ☐ Hotel
☐ Other

Hiv Knowledge

Have you previously tested for HIV?

○ No ○ Yes

Where was this test done?

○ I Can't Remember ○ SSWP ○ Zazi Clinic ○ Local Clinic ○ Hospital ○ Other

What was your test result?
(IF NEGATIVE, SKIP TO B6)

○ I Do Not Know ○ Negative ○ Positive ○ Indeterminate

Are you on treatment?
(IF NO, SKIP TO B6)

○ Never taken ○ On treatment always ○ stopped taking treatment

In the past week how many times have you taken your treatment?

○ All 7 Days ○ 5-6 Days ○ 3-4 Days ○ 1-2 Days ○ Not At All

What was the reason that you tested last time?

○ Peer Educator encouraged Me ○ I wasn't Feeling Well ○ Regular Checkup ○ Condom Bust ○ Rape/Sexual Assault ○ Concerned about my own Health ○ Partner is or was Ill ○ Partner is HIV Positive ○ Mcimbi was Ill ○ Mcimbi is HIV Positive ○ Family Member has HIV

Please tell me for each of the following, whether you think it is safe, probably safe, unsafe or you do not know.

Dry kissing

○ Safe ○ Probably Safe ○ Unsafe ○ I Don'T Know

Wet kissing/deep kissing

○ Safe ○ Probably Safe ○ Unsafe ○ I Don'T Know

Normal sex (vagina) with a condom

○ Safe ○ Probably Safe ○ Unsafe ○ I Don'T Know

Anal sex without a condom

○ Safe ○ Probably Safe ○ Unsafe ○ I Don'T Know

blow job (oral sex) without a condom

○ Safe ○ Probably Safe ○ Unsafe ○ I Don'T Know

Please tell me whether you strongly agree, agree, disagree or strongly disagree.

Sex workers are at high risk of contacting HIV

◯ Strongly Agree ◯ Agree ◯ Disagree ◯ Strongly Disagree

Non sex workers have a higher risk of getting HIV than sex workers

◯ Strongly Agree ◯ Agree ◯ Disagree ◯ Strongly Disagree

I do not need to use a condom with my main partner

◯ Strongly Agree ◯ Agree ◯ Disagree ◯ Strongly Disagree

If I contracted HIV, it could be cured/gotten rid of with medication/treatment from a doctor, traditional healer

◯ Strongly Agree ◯ Agree ◯ Disagree ◯ Strongly Disagree

Sex workers have less risk of experiencing violence than non sex workers

◯ Strongly Agree ◯ Agree ◯ Disagree ◯ Strongly Disagree

Experiencing violence increases the risk of getting HIV

◯ Strongly Agree ◯ Agree ◯ Disagree ◯ Strongly Disagree

Childhood

Please think back to your childhood and answer the following statements with never, sometimes, often or very often

I did not have enough to eat

◯ Never ◯ Sometimes ◯ Often ◯ Very Often

I lived in different households at different times

◯ Never ◯ Sometimes ◯ Often ◯ Very Often

I saw or heard by mother beaten by her husband or boyfriend

◯ Never ◯ Sometimes ◯ Often ◯ Very Often

I was told I was lazy or stupid or ugly by someone in my family

◯ Never ◯ Sometimes ◯ Often ◯ Very Often

Someone touched my thighs, buttocks, breasts or genitals when I did not want him to or made me touch his private parts when I did not want to

◯ Never ◯ Sometimes ◯ Often ◯ Very Often

I was insulted or humiliated by someone in my family in front of other people

◯ Never ◯ Sometimes ◯ Often ◯ Very Often

I was beaten at home with a belt or stick or whip or something else which was hard

◯ Never ◯ Sometimes ◯ Often ◯ Very Often

I had sex with a man who was more than 5 years older than me

◯ Never ◯ Sometimes ◯ Often ◯ Very Often

One or both of my parents were too drunk to take care of me

◯ Never ◯ Sometimes ◯ Often ◯ Very Often

I was beaten so hard at home that it left a mark or bruise

◯ Never ◯ Sometimes ◯ Often ◯ Very Often

I spent time outside the home and none of the adults at home knew where I was

◯ Never ◯ Sometimes ◯ Often ◯ Very Often

I had sex with someone who was not my boyfriend because I was threatenned or frightenned or forced

◯ Never ◯ Sometimes ◯ Often ◯ Very Often

Did you attend any kind of intiation school or go to the bush or mountain?
(IF NO, SKIP TO D1)

◯ No ◯ Yes

At this ceremony, did you experience any of the following?
(PLEASE SELECT ALL THAT APPLYIF SELECTED LABIAL STRETCHING, ASK QUESTIONS C15)

☐ I drank Beer/Spirits ☐ I was given drugs ☐ Taught about Pregnancy and Birthing ☐ Taught sex education ☐ How topPlease a husband ☐ How to keep secrets and not talk about problems ☐ Given a new name ☐ Malebe (Labia) or Clitoris was stretched or cut ☐ Touched in a sexual way by someone from initiation school ☐ Hurt sexualy by somebody at the school ☐ Forced to have sex

You have selected that your labia or clitoris was stretched or cut. Please tell me if you experienced any of the following while this was done or afterwards (related to this)
(PLEASE SELECT ALL THAT APPLY.)

☐ No Pain Or Discomfort ☐ It Was Very Painful ☐ Some Bleeding From My Vagina (Not Relating to A Monthly Period) ☐ Urinating Was Painful ☐ I Tried Not to Urinate So That It Did Not Hurt ☐ My Vagina/Lips Became Infected ☐ My Vagina/Lips Were Swollen ☐ A Girl (Other Than Myself) Became Very Ill On Camp ☐ A Girl Died On The Camp

How old were you when this happenned?

◯ 0-2 years ◯ 3-7 years ◯ 7-12 years ◯ 13-18 years ◯ >19 years

Gendernorms

Are you currently dating a man or woman?
(IF DATING MALE & FEMALE, ASK NEXT QUESTIONS ABOUT MALE PARTNER)

◯ Male ◯ Female ◯ Both ◯ No One

How old is your partner?

____
(IF NO PARTNER, ASK ABOUT MOST RECENT PARTNER)

Does your partner currently do anything to earn money?

◯ No ◯ Yes

Does your partner know that you sell sex?

◯ Yes ◯ No

Does your partner drink alcohol? How often?

◯ Never ◯ Every Day/Nearly Every Day ◯ ~~Only At Weekends~~ ◯ ~~A Few Times in A Month~~
◯ Less Than Once A Month ◯ I Don'T Know

Have you ever quarrelled or had any other conflict over his/her drinking?

◯ No ◯ Yes

Does your partner smoke dagga or take other drugs? How often?

◯ Never ◯ Every Day/Nearly Every Day ◯ Only At Weekends ◯ A Few Times in A Month
◯ Less Than Once A Month ◯ I Don'T Know

Pregnancy

How old were you when you first had sex?

____

Which of the following statements most closely describes your experiences the first time you had sexual intercourse?

◯ I Was Willing ◯ I Was Pursuaded ◯ I Was Tricked ◯ I Was Forced ◯ I Was Raped

Are you currently doing something or using any method to delay or avoid getting pregnant?
(IF NO, SKIP TO E4)

◯ No ◯ Yes

Which method are you using?

◯ The Injection ◯ Pill ◯ Condoms ◯ The Loop (IUD) ◯ Implant ◯ Herbal or Other Traditional Medicines ◯ Condoms and the Pill or Injection ◯ Withdrawal Before Sperming (Ejactulation)
◯ Other

Have you ever been pregnant?
(IF NO, SKIP TO E9 AND CONFIRM)

◯ No ◯ Yes

How old were you when you first became pregant

____

How many children have you given birth to ?

____

Have any of your children died after birth (this could be immediately afterwards or ever)?
(IF NO, SKIP TO E9 AND CONFIRM)

◯ No ◯ Yes

How many of your children have died?

____

Are you pregnant now?
(IF NO OR UNSURE, SKIP TO E11)

◯ No ◯ Yes ◯ Unsure

How many months pregnant are you?

____

Have you ever had a termination of pregnancy/abortion?
(IF UNSURE, PLEASE CLARIFY WHAT A TOP/ABOTION IS. THEN RE-ASK QUESTION.)

◯ No ◯ Yes ◯

In your lifetime, how many abortionss have you successfully had?

____

Violence

Please answer each statement with never, once, a few or many times. These questions are about your main partner, an ex or kwapheni.

Within the past year did any partner insult you or make you feel bad about yourself?
(IF NEVER, ASK 216 AS WELL)

○ Never ○ Once ○ Few ○ Many

Within the past year did any partner make a show off of you (belittle) or humiliate you in front of other people?
(IF NEVER, ASK 216 AS WELL)

○ Never ○ Once ○ Few ○ Many

Within the past year did any partner do things to scare or intimidate you on purpose for example by the way he looked at you, by yelling or smashing things?
(IF NEVER, ASK 216 AS WELL)

○ Never ○ Once ○ Few ○ Many

Within the past year did any partner threaten to hurt you?
(IF NEVER, ASK 216 AS WELL)

○ Never ○ Once ○ Few ○ Many

More than a year ago did any boyfriend do any of these things to you?

○ Yes ○ No

Within the past year did any partner slap you, push you or throw something at you which could hurt you?

○ Never ○ Once ○ Few ○ Many

Within the past year did any partner hit you with a fist or with something else (such as a beer bottle, stick or belt) which could hurt you?

○ Never ○ Once ○ Few ○ Many

Within the past year did any partner kick, drag, beat, choke or burn you?

○ Never ○ Once ○ Few ○ Many

Within the past year did any partner threaten to use or actually use a gun, knife or other weapon against you?

○ Never ○ Once ○ Few ○ Many

More than a year ago did any partner physically hurt you in any of these ways?

○ Yes ○ No

Within the past year did any partner physically force you to have sex when you did not want to?

○ Never ○ Once ○ Few ○ Many

Within the past year did you have sex with any partner when you did not want to because you were afraid of what he might do?

◯ Never ◯ Once ◯ Few ◯ Many

How many times has this happenned to you in the past 12 months

____

More than a year ago did any partner force you to do anything sexual when you did not want to?

◯ Yes ◯ No

When was the most recent time you were made to have sex with a partner when you did not want it?

____
(IF NEVER, ENTER 01-01-0001)

More than a year ago did any partner make a show off of you (belittle) or humiliate you in front of other people?

◯ Never ◯ Once ◯ Few ◯ Many

More than a year ago did any partner threaten to hurt you?

◯ Never ◯ Once ◯ Few ◯ Many

These questions are about mcimbi

Within the past year did any Mcimbi slap you, push you or throw something at you which could hurt you?

◯ Never ◯ Once ◯ Few ◯ Many

Within the past year did any Mcimbi hit you with a fist or with something else (such as a beer bottle, stick or belt) which could hurt you?

◯ Never ◯ Once ◯ Few ◯ Many

Within the past year did any Mcimbi kick, drag, beat, choke or burn you?

◯ Never ◯ Once ◯ Few ◯ Many

Within the past year did any Mcimbi threaten to use or actually use a gun, knife or other weapon against you?

◯ Never ◯ Once ◯ Few ◯ Many

More than a year ago did any Mcimbi physically hurt you in any of these ways?

◯ Yes ◯ No

Within the past year did any Mcimbi physically force you to have sex when you did not want to?

◯ Never ◯ Once ◯ Few ◯ Many

Within the past year did you have sex with any Mcimbi when you did not want to because you were afraid of what he might do?

◯ Never ◯ Once ◯ Few ◯ Many

How many times has this happenned to you in the past 12 months

____

More than a year ago did any Mcimbi force you to do anything sexual when you did not want to?

◯ Yes ◯ No

When was the most recent time you were made to have sex with an Mcimbi when you did not want it?

____
(IF NEVER, ENTER 01-01-0001)

These questions are about Police

Within the past year did any Police slap you, push you or throw something at you which could hurt you?

◯ Never ◯ Once ◯ Few ◯ Many

Within the past year did any Police hit you with a fist or with something else (such as a beer bottle, stick or belt) which could hurt you?

◯ Never ◯ Once ◯ Few ◯ Many

Within the past year did any Police kick, drag, beat, choke or burn you?

◯ Never ◯ Once ◯ Few ◯ Many

Within the past year did any Police threaten to use or actually use a gun, knife or other weapon against you?

◯ Never ◯ Once ◯ Few ◯ Many

More than a year ago did any Police physically hurt you in any of these ways?

◯ Yes ◯ No

Within the past year did any Police   physically force you to have sex when you did not want to?

◯ Never ◯ Once ◯ Few ◯ Many

Within the past year did you have sex with any Police   when you did not want to because you were afraid of what he might do?

◯ Never ◯ Once ◯ Few ◯ Many

How many times has this happenned to you in the past 12 months

____

More than a year ago did any Police force you to do anything sexual when you did not want to?

◯ Yes ◯ No

When was the most recent time you were made to have sex with a Policeman when you did not want it?

____
(IF NEVER, ENTER 01-01-0001)

Have you ever experienced gang rape?

◯ Yes ◯ No

How often has this happenned

____

Sex

When was the last time you had sex (normal (vagina)/anal/oral)?

____

The last time you had sex did you use a condom?
(IF NO, SKIP TO 278)

◯ Yes ◯ No

Did you use a condom for every round (a round is until he cums/ejaculates/sperms)?

◯ Yes ◯ No

Who brought the condom, was it you or your partner?

◯ Self ◯ Partner ◯ Both

Did you experience any of the following with the condom?

☐ Condom Breaking/Busting ☐ Condom Slipping Off ☐ Condom Only Put On Half Way
☐ Condom Was Removed During Sex ☐ No Problems,Condom Worked Perfectly

The last time you had sex, who was it with (a main partner, kwhapheni, mcimbi or nonpaying one night stand or ex-partner)?

◯ Regter/Main Partner/Vat 'N Sit ◯ Makhwapheni ◯ Mcimbi ◯ Ex Partner ◯ One Night Stand

Have you used condoms with your MAIN PARTNER in the past month? Would you say you used them always, often or sometimes?

◯ Not Used ◯ Always ◯ Often ◯ Sometimes

How many MAIN PARTNERS have you had sex with in the last year?

____

How many KHWAPHENI have had sex with during the past year?

____

How many one night stands have you had sex with during the past year?

____

Over the last year how often have you experienced the condom breaking or slipping off or only put it on half way through or have you taken it off and continued love making?

◯ Every Time Used ◯ Often ◯ Sometimes ◯ Never ◯ No Use

In the past 6 months have you experienced any of the folowing symptoms?
(IF NO SYMPTOMS EXPERIENCED, SKIP TO G14)

☐ Painful or Burning Vagina when Urinate ☐ Sores or Boils Around Vagina ☐ Itching Vagina
☐ Abnormal Vaginal Discharge ☐ Pain in The Bottom Of Your Stomach (Not Related To Your Menstrual Period Or Using The Loop) ☐ None Of These Symptoms

Did you receive treatment for the symptoms you just mentioned?

◯ Yes ◯ No

Have you ever been diagnosed with a sexually transmitted disease such as gonorrhea, syphilis, chlamydia, genital warts, or genital herpes

◯ Yes ◯ No ◯ Unsure ◯ Refused To Answer

Please select all of the different services that you offer

☐ Talking Or Companionship in Person ☐ Massage ☐ Dinner ☐ Breast Flashing ☐ Over The Phone Sex (Talking) ☐ Whatsapp/Sms Sex ☐ Internet/Email/Online Sex ☐ Normal Sex (vagina) ☐ Anal Sex ☐ blow job (oral sex) ☐ Hand Job ☐ Stripping Or Dancing ☐ Group Sex ☐ Other ☐

Please select the one services that is your most popular (that most mcimbi ask for)

◯ Talking Or Companionship in Person ◯ Massage ◯ Dinner ◯ Breast Flashing ◯ Over The Phone Sex (Talking) ◯ Whatsapp/Sms Sex ◯ Internet/Email/Online Sex ◯ Normal Sex (vagina) ◯ Anal Sex ◯ blow job (oral sex) ◯ Hand Job ◯ Stripping Or Dancing ◯ Group Sex ◯ Other ◯

In the past week, how many days did you have oral,anal or Normal sex (vagina) with mcimbi

____

In the past month, how many days did you work have oral, anal or Normal sex (vagina) with mcimbi

____
(MUST BE MORE THAN PREVIOUS)

How many one-time mcimbi did you have sex with in the last working DAY?

____

How many one-time mcimbi did you have sex with in the past WEEK?

____
(MUST BE MORE THAN PREVIOUS)

The last time you had sex with one-time mcimbi did you go with a condom?

◯ Without Condom ◯ Male Condom ◯ Female Condom ◯ Male and Female Condom

Do you always go with a condom with your one-time mcimbi?

◯ Every Time Used ◯ Often ◯ Sometimes ◯ Never ◯ No Use

How many of your regulars did you have sex with in the last working day?

____

How many of your regulars did you have sex with in the past week?

____
(MUST BE MORE THAN PREVIOUS)

The last time you had sex with regular mcimbi did you go with a condom?

◯ Without (Condom) ◯ Male Condom ◯ Female Condom ◯ Male and Female Condom

Has there been any occasion in the past month when either a regular or a one-time Mcimbi went without a condom for any reason when you were having sex?

◯ Yes ◯ No ◯ Refused Answer

Do you always work in the same place?

◯ Yes ◯ No

How many different places did you practise sex work in the past 6 months? (different taverns and different street corners, etc)

____

Have you ever worked as a sex worker in another province in South Africa

◯ Yes ◯ No

Have you ever worked as a sex worker in another country, not South Africa?

◯ Yes ◯ No

How much were you paid the last time you had Normal sex (vagina)?

____
(number)

How much did you earn the last day that you sold sex?

____
(number)

What is the maximum amount that you earned in 1 day this last month end (month end)

____
(number)

What is the minimum amount that you earned in 1 day this last month (mid month)

____
(number)

In the last month have you used anything to dry, clean or tighten your vagina before having sex? Have you done this every time, some times, just once or not at all?
(IF NEVER, SKIP TO G36)

◯ Everytime ◯ Sometimes ◯ Once ◯ Never

What have you used?
(PLEASE MARK ALL THAT APPLY)

☐ Soap ☐ Herbs/Snuff ☐ Ice/Water ☐ Zamlandela/Tiger Balm ☐ Alone Stone ☐ Brown Paper
☐ Ming Balls ☐ Other

In the last month have you used anything to lubricate your vagina before having sex? Have you done this every time, some times, just once or not at all?
(IF NEVER, SKIP TO G38)

◯ Everytime ◯ Sometimes ◯ Once ◯ Never

What have you used?
(PLEASE MARK ALL THAT APPLY)

☐ Lubricant ☐ Soap ☐ Spit ☐ Vaseline ☐ Baby Oil ☐ Other

In the last month have you used anything to hide that you were bleeding/mensruating, while having sex? Have you done this every time you bled, some times when you bled, just once or not at all?
(IF NEVER, SKIP TO G40)

◯ Everytime ◯ Sometimes ◯ Once ◯ Never

What have you used?
(PLEASE MARK ALL THAT APPLY)

☐ Tampon ☐ Kitchen Sponge ☐ Cloth ☐ Cotton Wool ☐ Toilet Paper ☐ Nacosa Condom
☐ Brown paper ☐ Other

Please answer each of the following statements with never, once, a few times or often.

Within the past year have you been verbally insulted, harassed or threatenned because you are a sex worker?

◯ Never ◯ Once ◯ A Few Times ◯ Often

Within the past year have you been beaten or threatenned because you are a sex worker?

◯ Never ◯ Once ◯ A Few Times ◯ Often

Within the past year have you experienced sexual abuse because you are a sex worker?

◯ Never ◯ Once ◯ A Few Times ◯ Often

Within the past year have any of your family (partner, children, siblings etc) experienced discrimination as a result of your being a sex worker?

◯ Never ◯ Once ◯ A Few Times ◯ Often

Within the past year how often have you been forced to change where you live because you are a sex worker?

◯ Never ◯ Once ◯ A Few Times ◯ Often

Within the past year have you been denied health services because you are a sex worker?

◯ Never ◯ Once ◯ A Few Times ◯ Often

Within the past year have you been denied police assistance/services because you are a sex worker?

◯ Never ◯ Once ◯ A Few Times ◯ Often

Please answer each of the following statements with strongly agree, agree, disagree or strongly disagree

Within the past year I have felt ashamed because I am a sex worker?

◯ Strongly Agree ◯ Agree ◯ Disagree ◯ Strongly Disagree

Within the past year I have felt guilty because I am a sex worker?

◯ Strongly Agree ◯ Agree ◯ Disagree ◯ Strongly Disagree

Within the past year I have blamed myself because I am a sex worker?

◯ Strongly Agree ◯ Agree ◯ Disagree ◯ Strongly Disagree

Within the past year I have blamed others because I am a sex worker?

◯ Strongly Agree ◯ Agree ◯ Disagree ◯ Strongly Disagree

Within the past year I have felt that I am not worth anything because I am a sex worker?

◯ Strongly Agree ◯ Agree ◯ Disagree ◯ Strongly Disagree

Within the past year I have felt punished because I am a sex worker?

◯ Strongly Agree ◯ Agree ◯ Disagree ◯ Strongly Disagree

Within the past year I have felt suicidal because I am a sex worker?

◯ Strongly Agree ◯ Agree ◯ Disagree ◯ Strongly Disagree

Within the past year I have chosen not to attend a party/bashes because of being a sex worker?

◯ Strongly Agree ◯ Agree ◯ Disagree ◯ Strongly Disagree

Within the past year I have isolated myself from friends and family because of being a sex worker?

◯ Strongly Agree ◯ Agree ◯ Disagree ◯ Strongly Disagree

Within the past year I have decided to not have (any more) children because of being a sex worker?

◯ Strongly Agree ◯ Agree ◯ Disagree ◯ Strongly Disagree

Within the past year I have avoided going to the clinic/hospital because of being a sex worker?

◯ Strongly Agree ◯ Agree ◯ Disagree ◯ Strongly Disagree

Within the past year I have avoided going to the police because of being a sex worker?

◯ Strongly Agree ◯ Agree ◯ Disagree ◯ Strongly Disagree

Mental Health

I would like to ask you some questions about how you have been feeling in the past week. I am going to read out some statements and ask you to say how many days you have had particular feelings or ideas or whether you have not had them at all.

During the past week I was worried by things that usually don't worry me

○ Rarely Or None Of The Time ○ Some Of A Little Of The Time (1-2Days) ○ A Moderate Amount Of Time (2-4 Days) ○ Most Of The Time (5-7Days)

During the past week I did not feel like eating, my appetite was poor

○ Rarely Or None Of The Time ○ Some Of A Little Of The Time (1-2Days) ○ A Moderate Amount Of Time (2-4 Days) ○ Most Of The Time (5-7Days)

During the past week I felt I could not make myself feel happy even with the help of family and friends

○ Rarely Or None Of The Time ○ Some Of A Little Of The Time (1-2Days) ○ A Moderate Amount Of Time (2-4 Days) ○ Most Of The Time (5-7Days)

During the past week I felt I was just as good as other people

○ Rarely Or None Of The Time ○ Some Of A Little Of The Time (1-2Days) ○ A Moderate Amount Of Time (2-4 Days) ○ Most Of The Time (5-7Days)

During the past week I had trouble keeping my mind on what I was doing

○ Rarely Or None Of The Time ○ Some Of A Little Of The Time (1-2Days) ○ A Moderate Amount Of Time (2-4 Days) ○ Most Of The Time (5-7Days)

During the past week I felt depressed (very sad all of the time)

○ Rarely Or None Of The Time ○ Some Of A Little Of The Time (1-2Days) ○ A Moderate Amount Of Time (2-4 Days) ○ Most Of The Time (5-7Days)

During the past week I felt that everything I did was an effort

○ Rarely Or None Of The Time ○ Some Of A Little Of The Time (1-2Days) ○ A Moderate Amount Of Time (2-4 Days) ○ Most Of The Time (5-7Days)

During the past week I felt hopeful about the future

○ Rarely Or None Of The Time ○ Some Of A Little Of The Time (1-2Days) ○ A Moderate Amount Of Time (2-4 Days) ○ Most Of The Time (5-7Days)

During the past week I thought my life had been a failure

○ Rarely Or None Of The Time ○ Some Of A Little Of The Time (1-2Days) ○ A Moderate Amount Of Time (2-4 Days) ○ Most Of The Time (5-7Days)

During the past week I felt fearful

○ Rarely Or None Of The Time ○ Some Of A Little Of The Time (1-2Days) ○ A Moderate Amount Of Time (2-4 Days) ○ Most Of The Time (5-7Days)

During the past week my sleep was restless (woke up in the night/did not sleep well/had trouble falling asleep)

◯ Rarely Or None Of The Time ◯ Some Of A Little Of The Time (1-2Days) ◯ A Moderate Amount Of Time (2-4 Days) ◯ Most Of The Time (5-7Days)

During the past week I was happy

◯ Rarely Or None Of The Time ◯ Some Of A Little Of The Time (1-2Days) ◯ A Moderate Amount Of Time (2-4 Days) ◯ Most Of The Time (5-7Days)

During the past week I talked less than usual

◯ Rarely Or None Of The Time ◯ Some Of A Little Of The Time (1-2Days) ◯ A Moderate Amount Of Time (2-4 Days) ◯ Most Of The Time (5-7Days)

During the past week I felt lonely

◯ Rarely Or None Of The Time ◯ Some Of A Little Of The Time (1-2Days) ◯ A Moderate Amount Of Time (2-4 Days) ◯ Most Of The Time (5-7Days)

During the past week people were unfriendly

◯ Rarely Or None Of The Time ◯ Some Of A Little Of The Time (1-2Days) ◯ A Moderate Amount Of Time (2-4 Days) ◯ Most Of The Time (5-7Days)

During the past week I enjoyed life

◯ Rarely Or None Of The Time ◯ Some Of A Little Of The Time (1-2Days) ◯ A Moderate Amount Of Time (2-4 Days) ◯ Most Of The Time (5-7Days)

During the past week I cried a lot

◯ Rarely Or None Of The Time ◯ Some Of A Little Of The Time (1-2Days) ◯ A Moderate Amount Of Time (2-4 Days) ◯ Most Of The Time (5-7Days)

During the past week I felt sick

◯ Rarely Or None Of The Time ◯ Some Of A Little Of The Time (1-2Days) ◯ A Moderate Amount Of Time (2-4 Days) ◯ Most Of The Time (5-7Days)

During the past week I felt that people dislike me

◯ Rarely Or None Of The Time ◯ Some Of A Little Of The Time (1-2Days) ◯ A Moderate Amount Of Time (2-4 Days) ◯ Most Of The Time (5-7Days)

During the past week I could not get 'going'

◯ Rarely Or None Of The Time ◯ Some Of A Little Of The Time (1-2Days) ◯ A Moderate Amount Of Time (2-4 Days) ◯ Most Of The Time (5-7Days)

During the past week I have been thinking too much

◯ Rarely Or None Of The Time ◯ Some Of A Little Of The Time (1-2Days) ◯ A Moderate Amount Of Time (2-4 Days) ◯ Most Of The Time (5-7Days)

During the past week my heart has been painful

◯ Rarely Or None Of The Time ◯ Some Of A Little Of The Time (1-2Days) ◯ A Moderate Amount Of Time (2-4 Days) ◯ Most Of The Time (5-7Days)

During the past week my spirit has been low

◯ Rarely Or None Of The Time ◯ Some Of A Little Of The Time (1-2Days) ◯ A Moderate Amount Of Time (2-4 Days) ◯ Most Of The Time (5-7Days)

Now I want to ask you a question about the past month (four weeks), Has the thought of ending your life been in your mind?

◯ No ◯ Yes

The following are symptoms that people sometimes experience after having a terrible event happen in their lives. I'll read each one, please state how much the symptom has bothered you in the past week

Recurrent thoughts or memories of the event

◯ Not At All ◯ Rarely ◯ Sometimes ◯ Most Of The Time

Feelings as though the event is happening again

◯ Not At All ◯ Rarely ◯ Sometimes ◯ Most Of The Time

Recurrent nightmares about the event

◯ Not At All ◯ Rarely ◯ Sometimes ◯ Most Of The Time

Sudden emotional or physical reactions when reminded of the event

◯ Not At All ◯ Rarely ◯ Sometimes ◯ Most Of The Time

Avoiding activities that remind you of the event

◯ Not At All ◯ Rarely ◯ Sometimes ◯ Most Of The Time

Avoiding thoughts or feelings associated with the event, so I try not to feel or think about anything that reminds me of it

◯ Not At All ◯ Rarely ◯ Sometimes ◯ Most Of The Time

Feeling jumpy, get a fright easily

◯ Not At All ◯ Rarely ◯ Sometimes ◯ Most Of The Time

Feeling on guard, ready to fight to protect yourself

◯ Not At All ◯ Rarely ◯ Sometimes ◯ Most Of The Time

In the past year, have you cut, burned or hurt yourself on purpose

◯ Yes ◯ No

In the past year, have you attempted suicide?

◯ Yes ◯ No

Audit

How often do you have a drink containing alcohol?

◯ Never ◯ Monthly Or Less ◯ 2-4Times Per Month ◯ 2-3 Times Per Week ◯ 4+ Times Per Week

How many drinks containing alcohol do you have on a typical day when you are drinking?

◯ 0 ◯ 1 Or 2 ◯ 3 Or 4 ◯ 5 Or 6 ◯ 7 Or 9 ◯ 10 Or More

How much is a typical drink (one drink, a beer or a glass of wine etc)

◯ no drink ◯ 250 mls or less (Small Beer or 1 glass of wine) ◯ 440mls ◯ 500mls
◯ 750 mls (Bumpie) ◯ 1 Litre ◯ 2 Litres ◯ 5 Litres (Big Box Wine)

How often do you have six or more drinks on one occasion?

◯ Never ◯ Less Than Monthly ◯ Monthly ◯ Weekly ◯ Daily Or Almost Daily

Within the past year how often have you taken dagga
(PLEASE SELECT ALL THAT APPLY)

◯ never ◯ once ◯ sometimes ◯ often

Within the past year how often have you taken Ndanda or mandrax

◯ never ◯ once ◯ sometimes ◯ often

Within the past year how often have you taken Nyaopi or whoonga

◯ never ◯ once ◯ sometimes ◯ often

Within the past year how often have you taken cough mixture when not sick

◯ never ◯ once ◯ sometimes ◯ often

Within the past year how often have you taken pain killers when not ill

◯ never ◯ once ◯ sometimes ◯ often

Within the past year how often have you taken Mg (Ecstacy)

◯ never ◯ once ◯ sometimes ◯ often

Within the past year how often have you taken Njumpi (Crystal Meth/Tik)|

◯ never ◯ once ◯ sometimes ◯ often

Within the past year how often have you taken Glass/Rock

◯ never ◯ once ◯ sometimes ◯ often

Self Esteem

For each statement please select either strongly agree, agree, disagree or strongly disagree.

Most of the time I am satisfied with myself

○ Strongly Agree ○ Agree ○ Disagree ○ Strongly Disagree

At times, I think I am no good at all

○ Strongly Agree ○ Agree ○ Disagree ○ Strongly Disagree

I feel that I have a number of good qualities

○ Strongly Agree ○ Agree ○ Disagree ○ Strongly Disagree

I am able to do things as well as most other people

○ Strongly Agree ○ Agree ○ Disagree ○ Strongly Disagree

I feel I do not have much to be proud of

○ Strongly Agree ○ Agree ○ Disagree ○ Strongly Disagree

I certainly feel useless at times

○ Strongly Agree ○ Agree ○ Disagree ○ Strongly Disagree

I feel that I'm a person of worth, at least equal or as good as other people

○ Strongly Agree ○ Agree ○ Disagree ○ Strongly Disagree

I wish I could have more respect for myself

○ Strongly Agree ○ Agree ○ Disagree ○ Strongly Disagree

Most of the time I feel as if I am a failure

○ Strongly Agree ○ Agree ○ Disagree ○ Strongly Disagree

I take a positive attitude toward myself

○ Strongly Agree ○ Agree ○ Disagree ○ Strongly Disagree

Final

END OF INTERVIEW

I would like to thank you very much for helping us. We have talked about some very difficult things today. I appreciate the time you have taken. I realise that these questions may have been difficult for you to answer, but it is only by hearing about women?s lives that we can really begin to understand them. We really appreciate your openness with us. Most women have difficult times in their lives and its good to share them and remember we did not bring them on ourselves. We really appreciate your participation in this study. By sharing this personal information with us and attending the SSWP peer talks and creative spaces you are helping us with our research and that will ultimately help many other sex workers in the country.

In the past 6 months have you received either of these items (SHOW A RED UMBRELLA CONDOM AND LUBRICANT) from a PE?

○ Yes
○ No

Last year, between January 2015 and December 2015, did you come to the SSWP clinic and do an HIV test? (ONLY YES IF THEY DID AN HIV TEST. DO NOT NOTE YES IF THEY DID ANYTHING ELSE WITH US!!!!!)

○ Yes ○ No

Could we contact you for any future research studies which may either be related to or unrelated to this study?

○ No ○ Yes

End Time of Interview

____

This interview took ___minutes to complete

____
(INSERT CALC FOR DATE TIME)

Hct

HIV COUNSELLING and TESTING

Thank you, for taking this survey with me. Now that we have completed the survey, I am going to to explain what an HIV test is and what the results mean. I will then test you and while we wait for the results we can discuss ways to reduce your risk. You have already signed consent to do the test, so we will not need to sign consent again now.

Pre Counselling Received
(EXPLAIN THE HCT PROCESS Two rapid test for HIV will be done by either myself or the nurse. A few drops of blood will be collected and tested on specific HIV testing kits to check for HIV antibodies. Test results will be ready in about 10 minutes. If both tests show us a negative result then it means that there are no antibodies to HIV. (Counsellor explains again about the window period). If both tests are positive, it means that you are HIV positive and that there are antibodies to HIV. Sometimes we cannot clearly tell if the results are negative or positive, so we have have 1 test show us a positive and the other show us a negative result. We will then have the nurse draw blood which we will send through to the laboratory for an HIV test called an ELISA to confirm the results. If you are HIV positive, they will also do your CD4 count and viral load. You will be able to get your laboratory results back from us in a week.)

○ Yes ○ No

First Rapid Test

○ Reactive (Positive) ○ Non-Reactive (Negative) ○ Indeterminate

Second Rapid Test

○ Reactive (Positive) ○ Non-Reactive (Negative) ○ Indeterminate

HIV NEGATIVEno further biological tests required. Participant can now return to coupon manager

HIV POSITIVE RESULTinform participant of their status and conduct CD4 and viral load. Once completed participant can return to coupon manager

INDETERMINATE RESULTCall nurse to draw blood and send to NHLS for HIV test (& CD4, Viral load). Once blood drawn participant can move on to coupon manager

Did you negotiate a risk reduction plan
(IDENTIFY RISK REDUCTION BEHAVIOURS. IT IS VERY IMPORTANT THAT THE PARTICIPANT UNDERSTANDS THAT THE USE OF CONDOMS WITH INTIMATE PARTNERS IS AS IMPORTANT AS IT IS WITH MCIMBI IN PROTECTING THEMSELVES, THEIR PARTNER AND THEIR CLIENTS, FROM HIV AND STIS. EXPLORE RISKY SEX WORKER BEHAVIOURS MENTIONED IN THEIR QUESTIONNAIRE AND WHICH THEY ARE ABLE & MOTIVATED TO CHANGE. IDENTIFY A STEP TOWARDS CHANGING THIS BEHAVIOUR. BREAK THE BEHAVIOUR DOWN INTO CONCRETE STEPS FOR THE CLIENT TO TRY. CHECK IN WITH THE CLIENT TO ENSURE THAT THE PLAN IS ACCEPTABLE AND ACTIONABLE FOR THEM - THEY SHOULD TAKE OWNERSHIP OF THE PLAN. YOU CAN ROLE PLAY THE PLAN TO ASSIST THE CLIENT. ASK THE CLIENT TO TRY THE PLAN OUT. MAKE SURE THEY KNOW THEY CAN COME BACK TO US FOR FUTURE ADVICE OR COUNSELLING TO ADAPT THIS PLAN. RECOGNISE HOW HARD IT CAN BE TO CHANGE BEHAVIOUR AND THE DANGERS SURROUNDING SEX WORK, BUT THAT THE PROGRAMME IS HERE TO SUPPORT THEM. IF THEY ARE NOT FAMILIAR WITH SSWP, PLEASE TELL THEM ABOUT US AND INVITE THEM TO COME TO OUR CREATIVE SPACE WORKSHOPS OR TAKE THEIR DETAILS DOWN TO DO OUTREACH IN THEIR AREA (SOWETO ONLY))

○ Yes ○ No

COMPLETED BY THE NURSE

Tests sent
(ONLY SEND UNKNOWN POSITIVES TO CLS)

☐ Confirmatory Elisa assay (NHLS) ☐ CD4 (NHLS) ☐ Viral Load (CLS) ☐ Drug Resistance (NICD)

CLS Laboratory Bar Code

____

NHLS Laboratory Bar Code

____

Confirmatory Elisa Result

◯ Negative ◯ Positive

CD4 count result

____
(NUMBER)

Viral Load result

____

B5 Art Drug Resistance

Drug Resistance Tested	◯ Yes<br>◯ No
Susceptible to all	◯ Yes<br>◯ No

Zidovudine	◯ S/PLLR	◯ LLR/IR	◯ HLR
Didanosine	◯ S/PLLR	◯ LLR/IR	◯ HLR
Stavudine	◯ S/PLLR	◯ LLR/IR	◯ HLR
Lamuvidine	◯ S/PLLR	◯ LLR/IR	◯ HLR
Emtricitabine	◯ S/PLLR	◯ LLR/IR	◯ HLR
Abacavir	◯ S/PLLR	◯ LLR/IR	◯ HLR
Tenofovir	◯ S/PLLR	◯ LLR/IR	◯ HLR
Nerviripine	◯ S/PLLR	◯ LLR/IR	◯ HLR
Efavirenz	◯ S/PLLR	◯ LLR/IR	◯ HLR
Etravirine	◯ S/PLLR	◯ LLR/IR	◯ HLR
Rilpivirine	◯ S/PLLR	◯ LLR/IR	◯ HLR
Indinavir/r	◯ S/PLLR	◯ LLR/IR	◯ HLR
Saquinavir/r	◯ S/PLLR	◯ LLR/IR	◯ HLR
Nelfinavir	◯ S/PLLR	◯ LLR/IR	◯ HLR
Fosamprenavir/r	◯ S/PLLR	◯ LLR/IR	◯ HLR
Lopinavir/r	◯ S/PLLR	◯ LLR/IR	◯ HLR
Atazanavir/r	◯ S/PLLR	◯ LLR/IR	◯ HLR
Tiplrnavir/r	◯ S/PLLR	◯ LLR/IR	◯ HLR
Darunavir/r	◯ S/PLLR	◯ LLR/IR	◯ HLR

Coupons

COUPON MANAGEMENT

This must be completed by the coupon manager for EVERY coupon given to this participant.

Number of coupons provided
◯ 0
◯ 1
◯ 2
◯ 3

Coupon 1 Number

____
(CHECK COUPON NUMBER AGAINST COUPON MANAGER SPREADSHEET. MAKE SURE YOU ACCURATELY COPY NUMBER ONTO COUPON!)

Coupon 2 Number

____
(CHECK COUPON NUMBER AGAINST COUPON MANAGER SPREADSHEET. MAKE SURE YOU ACCURATELY COPY NUMBER ONTO COUPON!)

Coupon 3 Number

____
(CHECK COUPON NUMBER AGAINST COUPON MANAGER SPREADSHEET. MAKE SURE YOU ACCURATELY COPY NUMBER ONTO COUPON!)

preferred reimbursement method

◯ Airtime ◯ Cash

valid mobile phone number

____
(PLEASE CONFIRM THAT THIS IS THE NUMBER WE WILL MAKE THEIR PAYMENT TO)

First two letters of your name, first two letters of your surname, year of birth, first two letters of your mothers' name __________________________________
