# Supplementary material for: Cross-sectional study of female sex workers in Soweto, South Africa: Factors associated with HIV infection
Source: PLoS One. 2017 Oct 5;12(10):e0184775. doi: 10.1371/journal.pone.0184775 (PMC5628807; doi:10.1371/journal.pone.0184775)
Supplement: S3 Text — (PDF) [file pone.0184775.s003.pdf]

S3 Text. Questionnaire (All Languages) (Pdf)

SCREENING TOOL

Coupon Number

____

Date of Screening

____

Have you scanned their fingerprint into the system? If so, are they a new participant?
(PLEASE DO NOT CONTINUE UNTIL THIS HAS BEEN DONE TO AVOID DUPLICATION INTO THE STUDY)

◯ Yes ◯ No

SCREENING FOR INCLUSION & EXCLUSION CRITERIA

Biological Gender (what you were born)

◯ Female ◯ Male ◯ Other

Self-identifies as a SW or self-identifies as working within a SW hotspot and consensually trading in sex for money or goods as her a primary source of income

◯ Yes ◯ No

Working (as a sex worker) in Soweto

◯ Yes ◯ No

Date of Birth

____
(IF DATE OF BIRTH UNKNOWN, ENTER AGE INTO S12, BELOW)

Age (if DoB unknown)

____

HIV status known or unknown

◯ Known ◯ Unknown

Current victim of human trafficking or sexual exploitation
(ANY INDIVIDUAL WHO REPORTS BEING A CURRENT VICTIM OF HUMAN TRAFFICKING OR EXPLOITATION MUST BE REFERRED TO THE DEPARTMENT OF SOCIAL DEVELOPMENT IMMEDIATELY.)

◯ Yes ◯ No

Are you part of the study team or did you participated in the pilot study for this study?

◯ Yes ◯ No

Do you believe this person is a sex worker

◯ yes, no secondary screening required ◯ yes, only after secondary screening ◯ no, after secondary screening. Please change them to a non sex worker above and do not enroll

SUCCESSFULLY SCREENED! :)Please enroll this person

Participant is NOT ELIGIBLEPlease do not enroll this person

If you were to be enrolled into this study, what language would you prefer to do the survey in?

◯ English ◯ Zulu ◯ Sotho

How many female sex workers do you know in Soweto? By 'know' I mean, people you know by name and would greet if you saw them, and they would greet you back

____
(YOU CAN TAKE YOUR TIME, THINK OF ALL THE FEMALE SEX WORKERS WHO ARE OVER THE AGE OF 18 YEARS AND WHO WORK IN SOWETO. THEY DO NOT HAVE TO WORK HERE EVERY DAY OF THE WEEK, BUT THEY DO NEED TO DO SELL SEX IN SOWETO SOMETIMES.)

Of those female sex workers, about how many are over the age of 18 years?

____
(THE NUMBER CANNOT EXCEED THE TOTAL NUMBER OF FEMALE SEX WORKERS KNOWN)

Of those female sex workers that you know, how many have you seen over the past month?

____
(THE NUMBER CANNOT EXCEED THE TOTAL NUMBER OF FEMALE SEX WORKERS KNOWN)

If you think about the the whole of Soweto, how many female sex workers do you estimate work across the whole of Soweto?

____
(TAKE HER TIME, ASSIST HER TO THINK OF THE WHOLE OF SOWETO, ALL 40 SUBURBS)

What are the main areas that you do business (sell sex) in Soweto?

- ☐ Braamfischerville
- ☐ Tshiawelo
- ☐ Devland
- ☐ Diepkloof
- ☐ Dlamini
- ☐ Dobsonville
- ☐ Doornkop
- ☐ Dube
- ☐ Embuzini
- ☐ Emdeni
- ☐ Freedom Park
- ☐ Greenvillage
- ☐ Jabavu
- ☐ Jabulani
- ☐ Klipspruit
- ☐ Kliptown
- ☐ Lakeside
- ☐ Mapetla
- ☐ Meadowlands
- ☐ Mmesi Park
- ☐ Mofolo
- ☐ Molapo
- ☐ Moletsane
- ☐ Moroka
- ☐ Mzimhlope
- ☐ Naledi
- ☐ Noordgesig
- ☐ Nomzamo Park
- ☐ Nancefield
- ☐ Orlando
- ☐ Phefeni
- ☐ Phiri
- ☐ Pimville
- ☐ Power Park
- ☐ Protea Glen
- ☐ Protea North
- ☐ Protea South
- ☐ Senaorane
- ☐ Tladi
- ☐ Zola
- ☐ Zondi
- ☐ White City
- ☐ Other Soweto
- ☐ Other outside Soweto

Now think about the main area that you work in in Soweto, how many female sex workers do you think work in just that part of the township, in that suburb (so not just the tavern or hostel)?

____

Preliminary

PRELIMINARY INFORMATION

Consented to both questionnaire and HIV tests

◯ Refused to Participate in Questionnaire and HIV Tests ◯ Agreed to Both Survey and HIV Tests
◯ Has Already Participated in The Study

Date and time of interview

____

Have you ever been to the SSWP or met with one of our peer educators prior to today?

☐ No I have not met a PE of been to The Wellness Center
☐ I have met a Peer Educator On Outreach
☐ I have been to The Wellness Center
☐ I have come to an SSWP creative space

What sex worker programmes in and around Soweto, or anywhere else in South Africa have you heard about? (PLEASE SELECT ALL THAT APPLY)

☐ No ☐ Sswp ☐ Sweat ☐ Sisonke ☐ Esselen Street/Wrhi ☐ Red Umbrella Project
☐ Another Sw Project

First name

____

Surname

____

Primary contact phone number

____

Demographic

QUESTIONNAIRE

Age

____

What suburb do you live in? By live in I mean, where do you normally sleep, for at least 3 nights/days of the week?

◯ Braamfischerville ◯ Tshiawelo ◯ Devland ◯ Diepkloof ◯ Dlamini ◯ Dobsonville
◯ Doornkop ◯ Dube ◯ Embuzini ◯ Emdeni ◯ Freedom Park ◯ Greenvillage
◯ Jabavu ◯ Jabulani ◯ Klipspruit ◯ Kliptown ◯ Lakeside ◯ Mapetla ◯ Meadowlands
◯ Mmesi Park ◯ Mofolo ◯ Molapo ◯ Moletsane ◯ Moroka ◯ Mzimhlope ◯ Naledi
◯ Noordgesig ◯ Nomzamo Park ◯ Nancefield ◯ Orlando ◯ Phefeni ◯ Phiri ◯ Pimville
◯ Power Park ◯ Protea Glen ◯ Protea North ◯ Protea South ◯ Senaorane ◯ Tladi
◯ Zola ◯ Zondi ◯ White City ◯ Other Soweto ◯ Other outside Soweto

Are you sexually attracted to women or men or both or don't know

◯ Women ◯ Men ◯ Both men and women ◯ Don't know

What racial group do you belong to?

◯ Black African ◯ Coloured ◯ White ◯ Indian/Asian ◯ Other

Where were you born?

◯ Gauteng ◯ Kwazulu Natal ◯ Western Cape ◯ Eastern Cape ◯ Norther Cape
◯ Freestate ◯ Mpumalanga ◯ Limpopo ◯ Zimbabwe ◯ Mozambique ◯ Malawi
◯ Botswana ◯ Uganda ◯ Other

When did you arrive in South Africa?

____

When did you come to live in Soweto?

____
(IF BORN IN SOWETO, ENTER 01-01-[YEAR OF BIRTH])

What is your home language?

◯ Zulu ◯ Ndebele ◯ Xhosa ◯ Northern Sotho ◯ Sesotho ◯ Setswana ◯ Siswati
◯ Tshivenda ◯ Xitsonga ◯ English ◯ Afrikaans ◯ Other

Did you ever go to school? If so, what is the highest grade you have ever successfully achieved (passed)?

◯ No Schooling ◯ Primary School Incomplete ◯ Primary School Completed ◯ High School Incomplete
◯ High School Completed ◯ Post School Qualification ◯

Are you currently studying?

◯ No ◯ Yes

Have either of your parents died?
(IF MOTHER, ANSWER A12IF NEITHER OR DO NOT KNOW SKIP TO A13)

◯ Neither ◯ Mother ◯ Father ◯ Mother and Father ◯ I Do Not Know

How old were you when your mother died?

____

How many living children do you have?

____
(IF NO CHILDREN, PLEASE ENTER IN 0. IF STILL PREGNANT WITH FIRST CHILD, PLEASE ENTER IN 0IF NO CHILDREN, SKIP TO A16)

Are any of your children under the age of 12?

◯ None Of Them ◯ 1 Child Is ◯ 2 Children Are ◯ 3 Children Are ◯ 4 Children Are
◯ 5 Children Are ◯ All Of Them Are Under 7 Years Of Age

Do any of your children currently live with you?

◯ No ◯ Yes

How many people including yourself live in your house (the place where you stay)?

____

How many rooms are in the house that you live in?

____

Do the people in your home go without food often, sometimes, seldom or never?

◯ Often ◯ Sometimes ◯ Seldom ◯ Never

Do the people in your home eat meat often, sometimes, seldom or never?

◯ Often ◯ Sometimes ◯ Seldom ◯ Never

If a person in your home became very ill and R100 was needed for the treatment or medicine, would you say that to find the money would be very easy, easy, quite difficult or very difficult?

◯ Very Easy ◯ Easy ◯ Quite Difficult ◯ Very Difficult

In the past month, how many people have relied on your earnings/money for food, shelter or school fees?

____
(PROBE ABOUT CHILDREN, PARENTS, SIBLINGS, PARTNER, FLAT MATES OR FRIENDS OR OTHER FAMILY REQUIRING SUPPORT. HELP THEM COUNT BY SHOWING FINGERS!)

Do you have any other activities other than sex work to support your livelihood?

Selling sex is my primary source of income

◯ No ◯ yes

I am employed by a company or person

◯ No ◯ yes

I am unemployed

◯ No ◯ yes

I receive a government grant such as a pension or child support grant

◯ No ◯ yes

I sell/trade goods such as vegetables or clothes to earn money

◯ No ◯ yes

In the last week, where do you normally sleep?

◯ Flat ◯ Hostel ◯ Tavern Back Room ◯ At Home With My Family ◯ A Private House With Other Sex Workers ◯ A Private House With No Other Sex Workers Live With Me ◯ A Brothel ◯ Abandonned Building ◯ On The Streets/Nowhere in Particular, Constantly On The Move ◯ Hotel ◯ Other

What is the main reason that you became a sex worker? Select the one which was most important to you at the time of your decision to become a sex worker

☐ Chosen Work ☐ No Other Jobs Available ☐ to Pay For Drugs/Alcohol ☐ I Needed Money ☐ Forced Into Sex Work ☐ Friend/Relative Who Was A Sex Worker Showed Me ☐ To be independent ☐ Other reason ☐

How many years have you been a sex worker for?

____
(ROUND TO THE NEAREST YEAR)

How old were you when you first sold sex?

____

Under what circumstances did you first sell sex?

◯ No Income to Buy Food Or For Shelter/Kicked Out ◯ Nowhere to stay, have nothing From Being Deserted, Becoming An Orphan Or A Widow/Divorcee ◯ to Add Onto The Family Income ◯ to Pay For Medical Costs in The Family Or For Self ◯ to Earn Fast Money Or Meet Personal Expenses ◯ Tricked Into Going to A Brothel ◯ Forced By Family ◯ Forced By Boyfriend ◯ Had to Pay Back Someone Through Sexual Favours ◯ Forced By Stranger ◯ Forced By friend(s) ◯ Teenage Pregnancy ◯ Other

Where do you normally pick up your mcimbi?
(PLEASE SELECT ALL THAT APPLY)

☐ Tavern ☐ Taxi Rank ☐ Truck Stop ☐ Prison ☐ Referral ☐ Street ☐ Hostel ☐ Hostel Taverns ☐ Internet ☐ Newpaper/Magazine Advertisements ☐ Brothel ☐ Strip/Dance Club ☐ Police ☐ Security ☐ Salon(Hair) ☐ Pick-Up (Road/Street Corner) ☐ Mine ☐ Hotel ☐ Other

O dula Motsetoropong ofe? Ka ho dula re bolela moo o robalang teng ka tlwaelo, bonyane masiu/matsatsi a 3 a beke?

◯ Braamfischerville ◯ Tshiawelo ◯ Devland ◯ Diepkloof ◯ Dlamini ◯ Dobsonville
◯ Doornkop ◯ Dube ◯ Embuzini ◯ Emdeni ◯ Freedom Park ◯ Greenvillage
◯ Jabavu ◯ Jabulani ◯ Klipspruit ◯ Kliptown ◯ Lakeside ◯ Mapetla ◯ Meadowlands
◯ Mmesi Park ◯ Mofolo ◯ Molapo ◯ Moletsane ◯ Moroka ◯ Mzimhlope ◯ Naledi
◯ Noordgesig ◯ Nomzamo Park ◯ Nancefield ◯ Orlando ◯ Phefeni ◯ Phiri ◯ Pimville
◯ Power Park ◯ Protea Glen ◯ Protea North ◯ Protea South ◯ Senaoane ◯ Tladi
◯ Zola ◯ Zondi ◯ White City ◯ Other Soweto ◯ Other outside Soweto

Ana o kgahlwa ke ho etsa thobalano le basadi kapa banna kapa bobedi ba bona kapa ha o tsebe?

◯ Basadi ◯ Banna ◯ Banna le basadi ka bobedi ◯ Ha o tsebe

O wa morabe ofe?

◯ Mothomotsho ◯ Lekhalate ◯ Lekgowa ◯ Moindia/Moeshia ◯ E mong

O hlahetse hokae?

◯ Gauteng ◯ Kwazulu Natal ◯ Western Cape ◯ Eastern Cape ◯ Norther Cape
◯ Freestate ◯ Mpumalanga ◯ Limpopo ◯ Zimbabwe ◯ Mozambique ◯ Malawi
◯ Botswana ◯ Uganda ◯ Other

O fihlile neng Aforika Borwa?

____

O tlile neng ho tla dula Soweto?

____
(IF BORN IN SOWETO, ENTER 01-01-[YEAR OF BIRTH])

Puo ya hao ya lapeng ke efe?

◯ Zulu ◯ Ndebele ◯ Xhosa ◯ Northern Sotho ◯ Sesotho ◯ Setswana ◯ Siswati
◯ Tshivenda ◯ Xitsonga ◯ English ◯ Afrikaans ◯ Other

Na o kile wa ya sekolong? Ebang ho le jwalo, o phasitse mophato ofe o ka hodimodimo?

◯ Ha o a kena sekolo ◯ Ha o a qeta Sekolo sa Poraemari ◯ O qetile Sekolo sa Poraemari
◯ Ha o a qeta Sekolo se Phahameng ◯ O qetile Sekolo se Phahameng ◯ O na le Lengolo la Thuto la Ka mora Sekolo ◯

Na ha jwale o a ithuta?

◯ Tjhe ◯ E

Na o na le motswadi ya hlokahetseng?
(IF MOTHER, ANSWER A12IF NEITHER OR DO NOT KNOW SKIP TO A13)

◯ Ha a yo ◯ Mme ◯ Ntate ◯ Mme le Ntate ◯ Ha ke tsebe

O ne o le dilemo tse kae ha mme wa hao a hlokahala?

____

O na le bana ba bakae ba phelang?

____
(F33)

Na o na le ngwana ofe kapa ofe wa dilemo tse ka tlase ho tse 12?

◯ Ha ho le a mong wa bona ◯ Ngwana a le 1 ◯ Bana ba babedi 2 ◯ Bana ba babedi 3
◯ Bana ba babedi 4 ◯ Bana ba babedi 5 ◯ Kaofela ha bona ba ka tlase ho dilemo tse 7

Na ho na le ngwana ofe kapa ofe wa hao eo ha jwale a dulang le wena?

◯ Tjhe ◯ E

Batho ba dulang ka tlung ya hao, ho kenyeletsa le wena, ba bakae? (moo o dulang)

____

Ntlo eo o dulang ho yona e na le diphaposi tse kae?

____

Na o ka bolela hore batho ba dulang ka tlung ya hao ba ye ba hloke dijo ha ngata, ka dinako tse ding, ha se ha ngata kapa ha ba ke ba hloke dijo?

◯ Ha ngata ◯ Ka dinako tse ding ◯ Ha se ha ngata ◯ Ha ba ke ba hloke

Na o ka bolela hore batho ba dulang ka tlung ya hao ba ye ba je nama ha ngata, ka dinako tse ding, ha se ha ngata kapa ha ba ke ba je nama?

◯ Ha ngata ◯ Ka dinako tse ding ◯ Ha se ha ngata ◯ Ha ba ke ba hloke

Haeba ho na le motho ka tlung ya hao ya kulang mme ho hlokeha R100 ya kalafo kapa moriana, na o ka bolela hore ho ka ba bobebe haholo, bobebe, boima kapa boima haholo ho fumana tjhelete?

◯ Bobebe haholo ◯ Bobebe ◯ Boima ◯ Boima haholo

Kgweding e fetileng, batho ba tshepetseng moputsong/tjheleteng ya hao bakeng sa dijo, bodulo kapa ditefello tsa sekolo ba ba kae?

____
(PROBE ABOUT CHILDREN, PARENTS, SIBLINGS, PARTNER, FLAT MATES OR FRIENDS OR OTHER FAMILY REQUIRING SUPPORT. HELP THEM COUNT BY SHOWING FINGERS!)

Do you have any other activities other than sex work to support your livelihood?

Horekisa mmele keyona feela kgwebo enkenyetsang tjhelete

◯ Tjhe ◯ e

Ke hirilwe ke khamphani kapa motho

◯ Tjhe ◯ e

Ha ke sebetse

◯ Tjhe ◯ e

Ke amohela kerante ya mmuso e jwalo ka phenshene kapa kerante ya ho hodisa ngwana

◯ Tjhe ◯ e

Ke rekisa/hweba ka dintho tse kang meroho kapa diaparo ho fumana tjhelete

◯ Tjhe ◯ e

Bekeng e sa tswa feta, o tlwaetse ho robala kae?

◯ Hostele ◯ Thaveneng Phaposing e ka Morao ◯ Lapeng le Ba lelapa la Heso ◯ Ntlong ya Poraevete le diSex Worker tse Ding ◯ Ntlong ya Poraevete e Se Nang diSex Worker tse Ding tse Dulang le Nna ◯ Brothele ◯ Moahong o Sa Hlokomelweng ◯ Diterateng/Ha ho moo ke dulang, Ke dula ke Tsamaya ◯ Hoteleng ◯ Sebakeng se Seng

Lebaka le leholo le entseng hore o be sex worker ke lefe? Kgetha leo e neng e le la bohlokwa ho wena ka nako eo o nkang qeto ya ho ba sex worker

☐ Ke Mosebetsi oo ke o Kgethileng ☐ Ha ho na Mesebetsi e Meng e Fumanehang ☐ ho Lefella Dithethefatsi/Jwala ☐ Ke ne ke Hloka Tjhelete ☐ Ke Qobellehile ho etsa Sex Work ☐ Motswalle/E mong wa leloko eo e neng e le Sex Worker o Mpontshitse ☐ Ho re ke e emele ☐ Lebaka le Leng ☐

O na le dilemo tse kae o le sex worker?

____
(ROUND TO THE NEAREST YEAR)

O ne o le dilemo tse kae ha o qala ho rekisa thobalano?

____

O qadile ho rekisa thobalano ka mabaka afe?

◯ Ha ho na tjhelete ya ho reka Dijo kapa ya Bodulo/Lelekilwe ◯ Ha ho na Tshireletso E bonahalang/ya Nnete ya Ho lahlwa, Ho ba Kgutsana kapa Mohlolohadi/Motho ya Hladileng ◯ ho Eketsa Moputsong wa Lelapa ◯ Ho Lefella Ditjeho tsa Bongaka tsa Lelapa kapa tsa Ka ◯ Ho Fumana Tjhelete e Fumanehang ka Potlako kapa Ho Lefella Ditshenyehelo ◯ Qhekanyeditswe ho ya Brotheleng ◯ Qobelletswe ke ba Lelapa ◯ Qobelletswe ke Mohlankana ◯ Ke ne ke tlamehile ho lefa Motho e Mong ka ho etsa Thobalano ◯ Qobelletswe ke Motho eo ke sa mo tsebeng ◯ Qobelletswe ke ba bang ba Lelapa ◯ Ho ba Mmeleng ha Batjha ◯ E nngwe

Hangata mcimbi wa hao o o lata ho kae?
(PLEASE SELECT ALL THAT APPLY)

☐ Thaveneng ☐ Renkeng ya Ditekesi ☐ Setopong sa Dilori ☐ Tjhankaneng ☐ Rometswe ☐ Seterateng ☐ Hostele ☐ Dithaveneng tsa Hostele ☐ Inthaneteng ☐ Dipapatsong tsa Koranta/Mekesine ☐ Brothel ☐ Tlelapong ya Metantsho ya batho ba Hlobolang ha ba Tantsha ☐ Sepoleseng ☐ Tshireletsong ☐ Salunung (Moriri) ☐ Moo ho engwang teng (Hukung ya Mmila/Seterata) ☐ Maeneng ☐ E nngwe

Uhlala kweliphi idolobhana? Ngokuhlala, ngisho ukuthi yikuphi lapho ulala khona ngokuvamile, okungenani ubusuku/izinsuku ezi-3 ngeviki.

◯ Braamfischerville ◯ Tshiawelo ◯ Devland ◯ Diepkloof ◯ Dlamini ◯ Dobsonville
◯ Doornkop ◯ Dube ◯ Embuzini ◯ Emdeni ◯ Freedom Park ◯ Greenvillage
◯ Jabavu ◯ Jabulani ◯ Klipspruit ◯ Kliptown ◯ Lakeside ◯ Mapetla ◯ Meadowlands
◯ Mmesi Park ◯ Mofolo ◯ Molapo ◯ Moletsane ◯ Moroka ◯ Mzimhlope ◯ Naledi
◯ Noordgesig ◯ Nomzamo Park ◯ Nancefield ◯ Orlando ◯ Phefeni ◯ Phiri ◯ Pimville
◯ Power Park ◯ Protea Glen ◯ Protea North ◯ Protea South ◯ Senaoane ◯ Tladi
◯ Zola ◯ Zondi ◯ White City ◯ Other Soweto ◯ Other outside Soweto

Ingabe uthanda ukwenza ucansi nabantu besifazane noma namadoda noma kokubili noma awazi

◯ Abesifazane ◯ Amadoda ◯ Kokubili amadoda nabesifazane ◯ Angazi

Ungowaluphi uhlanga?

◯ Um-Afrika Omnyama ◯ Umkhaladi ◯ Umlungu ◯ UmNdiya/Um-Eshiya ◯ Okunye

Wazalelwaphi?

◯ Gauteng ◯ KwaZulu Natali ◯ Ntshonalanga Kapa ◯ Mpumalanga Kapa ◯ Nyakatho Kapa
◯ Freyistata ◯ Mpumalanga ◯ Limpopo ◯ Zimbabwe ◯ Mozambique ◯ Malawi
◯ Botswana ◯ Uganda ◯ Elinye

Wafika nini eNingizimu Afrika?

____

Wafika nini ukuzohlala eSoweto?

____
(IF BORN IN SOWETO, ENTER 01-01-[YEAR OF BIRTH])

Yiluphi ulimi lwakho lwasekhaya?

◯ isiZulu ◯ isiNdebele ◯ isiXhosa ◯ IsiSuthu SaseNyakatho ◯ isiSuthu ◯ isiTswana
◯ isiSwati ◯ isiVenda ◯ isiTsonga ◯ isiNgisi ◯ isiBhunu ◯ Olunye

Wake wasifunda isikole? Uma kunjalo, yiliphi ibanga eliphezulu kakhulu owaliqeda ngempumelelo (owaliphasa)?

◯ Asikho Isikole ◯ Asiqedwanga Isikole Samabanga Aphansi ◯ Siqediwe Isikole Samabanga Aphansi
◯ Asiqedwanga Isikole Samabanga Aphezulu ◯ Isiqediwe Isikole Samabanga Aphezulu
◯ Iziqu Zangemuva Kwesikole ◯

Uyafunda yini njengamanje?

◯ Cha ◯ Yebo

Ukhona yini kubazali bakho owashona?
(IF MOTHER, ANSWER A12IF NEITHER OR DO NOT KNOW SKIP TO A13)

◯ Akekho ◯ Umama ◯ Ubaba ◯ Umama noBaba ◯ Angazi

Wawuneminyaka emingaki ngesikhathi umama wakho eshona?

____

Unezingane ezingaki eziphilayo?

____
(F33)

Ikhona yini ezinganeni zakho engaphansi kweminyaka eyi-12 ubudala?

◯ Akekho Kubo ◯ ingane1 ◯ Izingane 2 ◯ Izingane 3 ◯ Izingane 4 ◯ Izingane 5
◯ Zonke Kuzo Zingaphansi Kweminyaka eyi-7 Ubudala

Ikhona yini ezinganeni zakho ehlala nawe njengamanje?

◯ Cha ◯ Yebo

Bangaki abantu kubalwa nawe abahlala endlini yakho (indawo lapho ohlala khona)?

____

Mangaki amagumbi endlini ohlala kuyo?

____

Ungasho yini ukuthi abantu abasemzini wakho kaningi, ngezinye izikhathi, akuvamile noma akaze bahlale bengadlile?

◯ Kaningi ◯ Ngezinye izikhathi ◯ Akuvamile ◯ Neze nhlobo

Ungasho yini ukuthi abantu abasemzini wakho kaningi, ngezinye izikhathi, akuvamile noma abayidli nhlobo inyama?

◯ Kaningi ◯ Ngezinye izikhathi ◯ Akuvamile ◯ Neze nhlobo

Uma umuntu osemzini wakho egula kakhulu futhi kudingeka u-R100 ukumelapha noma umuthi, ungasho yini ukuthi kuzoba lula kakhulu, kuzoba lula, kuzoba nzima noma kuzoba nzima kakhulu ukuthola imali?

◯ Kulula Kakhulu ◯ Kulula ◯ Kunzima Impela ◯ Kunzima Kakhulu

Ngenyanga edlule, bangaki abantu abebethembele emalini yakho oyiholayo/emalini yokudla, indawo yokukhosela noma izimali zesikole?

____
(PROBE ABOUT CHILDREN, PARENTS, SIBLINGS, PARTNER, FLAT MATES OR FRIENDS OR OTHER FAMILY REQUIRING SUPPORT. HELP THEM COUNT BY SHOWING FINGERS!)

Do you have any other activities other than sex work to support your livelihood?

Ukudayisa umzimba iyona ndlela engingenisela imali

◯ Cha ◯ yebo

Ngiqashwe yinkampani noma wumuntu

◯ Cha ◯ yebo

Angisebenzi

◯ Cha ◯ yebo

Ngithola isibonelelo sikahulumeni esifana nempesheni noma isibonelelo sokondla ingane

◯ Cha ◯ yebo

Ngithengisa /ngihweba ngezimpahla ezifana nemifino noma izimpahla zokugqoka ukuthi ngithole imali

◯ Cha ◯ yebo

Esontweni eledlule, ulala kuphi ngokuvamile?

◯ Ehostele ◯ Igumbi Elingemuva Ethaveni ◯ Ekhaya Nomndeni Wami ◯ Indlu Engasese Namanye Ama-Sex Worker ◯ Indlu Engasese Akukho Amanye Ama-Seex Worker Ahlala Nami ◯ Indawo Esebenzela Ama-Sex Worker ◯ Ibhilidi Elilaxaziwe ◯ Emigwaqeni/Akukho Ndawo Thizeni, Njalo Uyahamba ◯ Ihhotela ◯ Okunye

Yisiphi isizathu esikhulu esikwenze ukuthi ube yi-sex worker? Khetha lokho okwakubaluleke kunakho konke ngaleso sikhathi ngesinqumo sakho sokuba yi-sex worker

☐ Umsebenzi Engiwukhethile ☐ Awukho Omunye Umsebenzi Okhona ☐ Ukukhokhela Izidakamizwa/Utshwala ☐ I Bekudingeka Imali ☐ Uphoqelelwe Emsebenzini Wocansi ☐ Umngane/Isihlobo Esasiyi-Sex Worker Sangikhombisa ☐ Ukuthi ngizimele ☐ Esinye Isizathu ☐

Mingaki iminyaka uyi-sex worker?

____
(ROUND TO THE NEAREST YEAR)

Wawungakanani lapho uqala ukuthengisa ucansi?

____

Waluthengisa ngaphansi kwaziphi izimo ucansi okokuqala?

◯ Ayikho Imali Engenayo Yokuthenga Ukudla Noma Indawo Yokuhlala/Ukhishiwe ◯ Akukho Ukuvikeleka Komzimba Ekushiyweni, Ube Yintandane Noma Umfelokazi/Udivosile ◯ Ukwengeza Emalini Yomndeni Engenayo ◯ Yokukhokha Izindleko Zokwelashwa eMndenini noma zakho Uqobo ◯ Ukuthola Imali Esheshayo Noma Ukuhlangabezana Nezindleko Zakho Siqu ◯ Waphicwa Ukuthi Uye Endaweni Yama-Sex Worker ◯ Waphoqwa Umndeni ◯ Waphoqwa Yisoka ◯ Kwakufanele Ukhokhele Umuntu Othize Ngamafeyiva Ocansi ◯ Waphoqwa Umuntu Ongamazi ◯ Waphoqwa Ngomunye Umndeni ◯ Ukukhulelwa Kwengane Engena Ebudaleni ◯ Okunye

Uvame ukuwuthatha kuphi umcimbi wakho?
(PLEASE SELECT ALL THAT APPLY)

☐ Ithaveni ☐ Irenki Yamatekisi ☐ Indawo Yokuma Amathilagi ☐ Ijele ☐ Ukudluliselwa ☐ Umgwaqo ☐ Ehostele ☐ Amathaveni Amahostela ☐ Inthanethi ☐ Izikhangisi Zephephandaba/Zephephabhuku ☐ Indawo esetshenziswa ama-sex worker ☐ I-Strip/Dance Club ☐ Amaphoyisa ☐ i-Security ☐ iSalon(Izinwele) ☐ Pick-Up (Umgwaqo/Ikhona Lomgwaqo) ☐ Imayini ☐ Okunye

Hiv Knowledge

Have you previously tested for HIV?

◯ No ◯ Yes

Where was this test done?

◯ I Can't Remember ◯ SSWP ◯ Zazi Clinic ◯ Local Clinic ◯ Hospital ◯ Other

What was your test result?
(IF NEGATIVE, SKIP TO B6)

◯ I Do Not Know ◯ Negative ◯ Positive ◯ Indeterminate

Are you on treatment?
(IF NO, SKIP TO B6)

◯ Never taken ◯ On treatment always ◯ stopped taking treatment

In the past week how many times have you taken your treatment?

◯ All 7 Days ◯ 5-6 Days ◯ 3-4 Days ◯ 1-2 Days ◯ Not At All

What was the reason that you tested last time?

◯ Peer Educator encouraged Me ◯ I wasn't Feeling Well ◯ Regular Checkup ◯ Condom Bust ◯ Rape/Sexual Assault ◯ Concerned about my own Health ◯ Partner is or was Ill ◯ Partner is HIV Positive ◯ Mcimbi was Ill ◯ Mcimbi is HIV Positive ◯ Family Member has HIV

Please tell me for each of the following, whether you think it is safe, probably safe, unsafe or you do not know.

Dry kissing

◯ Safe ◯ Probably Safe ◯ Unsafe ◯ I Don'T Know

Wet kissing/deep kissing

◯ Safe ◯ Probably Safe ◯ Unsafe ◯ I Don'T Know

Normal sex (vagina) with a condom

◯ Safe ◯ Probably Safe ◯ Unsafe ◯ I Don'T Know

Anal sex without a condom

◯ Safe ◯ Probably Safe ◯ Unsafe ◯ I Don'T Know

blow job (oral sex) without a condom

◯ Safe ◯ Probably Safe ◯ Unsafe ◯ I Don'T Know

Please tell me whether you strongly agree, agree, disagree or strongly disagree.

Sex workers are at high risk of contacting HIV

○ Strongly Agree ○ Agree ○ Disagree ○ Strongly Disagree

Non sex workers have a higher risk of getting HIV than sex workers

○ Strongly Agree ○ Agree ○ Disagree ○ Strongly Disagree

I do not need to use a condom with my main partner

○ Strongly Agree ○ Agree ○ Disagree ○ Strongly Disagree

If I contracted HIV, it could be cured/gotten rid of with medication/treatment from a doctor, traditional healer

○ Strongly Agree ○ Agree ○ Disagree ○ Strongly Disagree

Sex workers have less risk of experiencing violence than non sex workers

○ Strongly Agree ○ Agree ○ Disagree ○ Strongly Disagree

Experiencing violence increases the risk of getting HIV

○ Strongly Agree ○ Agree ○ Disagree ○ Strongly Disagree

Ana o kile wa etsa teko ya HIV nakong e fetileng?

○ Tjhe ○ E

Teko ena e etseditswe ho kae?

○ Ha ke hopole ○ SSWP ○ Tleleniking ya Zazi ○ Tleleniking ya Lehae ○ Sepetlele
○ E nngwe

Sephetho sa teko ya hao e ne e le sefe?

○ Ha Ke Tsebe ○ Nekethifi ○ Phosethifi ○ Ha se a Fumaneha

Ana o sebedisa kalafo?

○ Never taken ○ On treatment always ○ stopped taking treatment

Bekeng e sa tswa feta, o nwele kalafo ya hao makgetlo a makae?

○ Matsatsi a 7 Kaofela ○ Matsatsi a 5-6 ○ Matsatsi a 3-4 ○ Letsatsi le le 1-2 ○ Ho hang

Lebaka la hore o etse teko lekgetlong le fetileng e ne e le lefe?

○ Peer Educator e Nkgothaleditse ○ Ke ne ke sa Ikutlwe Hantle ○ Ho Hlola ha Ka mehla
○ Khondomo e Phunyehile ○ Peto/Utlwiswa Bohloko ka Thobalano ○ Ngongorehile ka Bophelo ba Ka
○ Molekane o a kapa o ne a kula ○ Molekane o HIV Phosethifi ○ Mcimbi o ne o sa Phela Hantle
○ Mcimbi o HIV Phosethifi ○ Setho sa Lelapa se na le HIV

Please tell me for each of the following, whether you think it is safe, probably safe, unsafe or you do not know.

Ho sunana ho sa sebediseng leleme

◯ Bolokehile ◯ Ho na le Kgonahalo ya Ho bolokeha ◯ Ha ho a Bolokeha ◯ Ha Ke Tsebe

Ho sunana ha ho sebedisa leleme

◯ Bolokehile ◯ Ho na le Kgonahalo ya Ho bolokeha ◯ Ha ho a Bolokeha ◯ Ha Ke Tsebe

Ho robalana ha ho kenya setho sa bonna ka kukung ka khondomo

◯ Bolokehile ◯ Ho na le Kgonahalo ya Ho bolokeha ◯ Ha ho a Bolokeha ◯ Ha Ke Tsebe

Ho robalana ha ho kenya setho sa bonna ka maraong ntle lekhondomo

◯ Bolokehile ◯ Ho na le Kgonahalo ya Ho bolokeha ◯ Ha ho a Bolokeha ◯ Ha Ke Tsebe

Ho nyeka ditho tsa bonna le bosadi ntle le khondomo

◯ Bolokehile ◯ Ho na le Kgonahalo ya Ho bolokeha ◯ Ha ho a Bolokeha ◯ Ha Ke Tsebe

Please tell me whether you strongly agree, agree, disagree or strongly disagree.

Disex worker di na le kotsi e nyane ya ho ba le HIV ho feta bao e seng disex worker

◯ Ke Dumela Ke Tiile ◯ Ke a Dumela ◯ Ke a Hana ◯ Ke Hana ke Tiile

Motho eo eleng non sex worker ona le kotsi e kgolo ya ho ba le HIV ho feta sex worker

◯ Ke Dumela Ke Tiile ◯ Ke a Dumela ◯ Ke a Hana ◯ Ke Hana ke Tiile

Ha ke hloke ho sebedisa khondomo le regte wa ka (molekane wa bohlokwa)

◯ Ke Dumela Ke Tiile ◯ Ke a Dumela ◯ Ke a Hana ◯ Ke Hana ke Tiile

Ha ke tshwaetswa ke HIV, e ka alafuwa/fediswa ka meriana/kalafo e tswang ngakeng, ngakeng ya setho

◯ Ke Dumela Ke Tiile ◯ Ke a Dumela ◯ Ke a Hana ◯ Ke Hana ke Tiile

Disex worker di na le kotsi e nyane ya ho ba diketsong tsa dikgoka ho feta bao e seng disex worker

◯ Ke Dumela Ke Tiile ◯ Ke a Dumela ◯ Ke a Hana ◯ Ke Hana ke Tiile

Ho ba diketsong tsa dikgoka ho eketsa kotsi ya ho ba le HIV

◯ Ke Dumela Ke Tiile ◯ Ke a Dumela ◯ Ke a Hana ◯ Ke Hana ke Tiile

Uke wahlola i-HIV esikhathini esedlule?
(IF HAS NO, SKIP TO B7)

◯ Cha ◯ Yebo

Kwenziwa kuphi lokhu kuhlolwa?

◯ Angikhumbuli ◯ I-SSWP ◯ I-Zazi Clinic ◯ Umtholampilo Wasekhaya ◯ Isibhedlela
◯ Okunye

Wathini umphumela wakho?
(IF NEGATIVE, SKIP TO B6)

◯ Angazi ◯ Negethivu ◯ Phozethivu ◯ Akwaziwa

Usebenzisa umuthi wokwelashwa?
(IF NO, SKIP TO B6)

◯ Never taken ◯ On treatment always ◯ stopped taking treatment

Ngesonto eledlule uwuthathe izikhathi ezingaki umuthi wakho wokwelashwa?

◯ Zonke Izinsuku eziwu-7 ◯ 5-6 Izinsuku ◯ 3-4 Izinsuku ◯ 1-2 Izinsuku ◯ Lutho Nhlobo

Kwakuyini isizathu sokuthi uhlolwe ngesikhathi esedlule?

◯ Umfundisi Onguntanga wangikhuthaza ◯ Ngangingaphathekile Kahle ◯ Ukuhlolwa Okuvamile
◯ Ukuqhuma Kwekhondomu ◯ Ukudlwengula/Ukuhlaselwa Ngokocansi ◯ Ngikhathazekile ngeMpilo yami uqobo ◯ Umlingani uyagula noma wayegula ◯ Umlingani une-HIV ◯ UMcimbi wawugula
◯ UMcimbi une-HIV ◯ Ilungu Lomndeni Line-HIV

Please tell me for each of the following, whether you think it is safe, probably safe, unsafe or you do not know.

Ukuqabula ungafaki ulimi emlonyeni

◯ Kuphephile ◯ Mhlawumbe Kuphephile ◯ Akuphephile ◯ Angazi

Ukuqabula ufake ulimi emlonyeni

◯ Kuphephile ◯ Mhlawumbe Kuphephile ◯ Akuphephile ◯ Angazi

Ucansi esithweni somuntu wesifazane ngekhondomu

◯ Kuphephile ◯ Mhlawumbe Kuphephile ◯ Akuphephile ◯ Angazi

Ucansi endunu ngaphandle kwe khondomu

◯ Kuphephile ◯ Mhlawumbe Kuphephile ◯ Akuphephile ◯ Angazi

Ucansi ngomlomo ngaphandle kwekhondomu

◯ Kuphephile ◯ Mhlawumbe Kuphephile ◯ Akuphephile ◯ Angazi

Please tell me whether you strongly agree, agree, disagree or strongly disagree.

Ama-sex workers asengozini enkulu yokuthola i-HIV

◯ Ngiyavuma Kakhulu ◯ Ngiyavuma ◯ Angivumi ◯ Angivumi Kakhulu

Umuntu ongasiyona isex worker usengozini enkulu yokuthola iHIV kunoyi sex worker

◯ Ngiyavuma Kakhulu ◯ Ngiyavuma ◯ Angivumi ◯ Angivumi Kakhulu

Asikho isidingo sokuthi ngisebenzise ikhondomu nomaqondana wami (umlingani wangempela)

◯ Ngiyavuma Kakhulu ◯ Ngiyavuma ◯ Angivumi ◯ Angivumi Kakhulu

Uma ngithola i-HIV, ingelapheka/ingapheliswa ngomuthi/ngokwelashwa udokotela, inyanga yesintu

◯ Ngiyavuma Kakhulu ◯ Ngiyavuma ◯ Angivumi ◯ Angivumi Kakhulu

Amasex worker asengozini encane yokuhlangabezana nodlame kunalabo abangewona ama-sex worker

◯ Ngiyavuma Kakhulu ◯ Ngiyavuma ◯ Angivumi ◯ Angivumi Kakhulu

Ukuhlangabezana nodlame kwandisa ingozi yokuthola i-HIV

◯ Ngiyavuma Kakhulu ◯ Ngiyavuma ◯ Angivumi ◯ Angivumi Kakhulu

Childhood

Please think back to your childhood and answer the following statements with never, sometimes, often or very often

I did not have enough to eat

◯ Never ◯ Sometimes ◯ Often ◯ Very Often

I lived in different households at different times

◯ Never ◯ Sometimes ◯ Often ◯ Very Often

I saw or heard by mother beaten by her husband or boyfriend

◯ Never ◯ Sometimes ◯ Often ◯ Very Often

I was told I was lazy or stupid or ugly by someone in my family

◯ Never ◯ Sometimes ◯ Often ◯ Very Often

Someone touched my thighs, buttocks, breasts or genitals when I did not want him to or made me touch his private parts when I did not want to

◯ Never ◯ Sometimes ◯ Often ◯ Very Often

I was insulted or humiliated by someone in my family in front of other people

◯ Never ◯ Sometimes ◯ Often ◯ Very Often

I was beaten at home with a belt or stick or whip or something else which was hard

◯ Never ◯ Sometimes ◯ Often ◯ Very Often

I had sex with a man who was more than 5 years older than me

◯ Never ◯ Sometimes ◯ Often ◯ Very Often

One or both of my parents were too drunk to take care of me

◯ Never ◯ Sometimes ◯ Often ◯ Very Often

I was beaten so hard at home that it left a mark or bruise

◯ Never ◯ Sometimes ◯ Often ◯ Very Often

I spent time outside the home and none of the adults at home knew where I was

◯ Never ◯ Sometimes ◯ Often ◯ Very Often

I had sex with someone who was not my boyfriend because I was threatenned or frightenned or forced

◯ Never ◯ Sometimes ◯ Often ◯ Very Often

Did you attend any kind of intiation school or go to the bush or mountain?
(IF NO, SKIP TO D1)

◯ No ◯ Yes

At this ceremony, did you experience any of the following?
(PLEASE SELECT ALL THAT APPLYIF SELECTED LABIAL STRETCHING, ASK QUESTIONS C15)

☐ I drank Beer/Spirits ☐ I was given drugs ☐ Taught about Pregnancy and Birthing ☐ Taught sex education ☐ How topPlease a husband ☐ How to keep secrets and not talk about problems ☐ Given a new name ☐ Malebe (Labia) or Clitoris was stretched or cut ☐ Touched in a sexual way by someone from initiation school ☐ Hurt sexualy by somebody at the school ☐ Forced to have sex

You have selected that your labia or clitoris was stretched or cut. Please tell me if you experienced any of the following while this was done or afterwards (related to this)
(PLEASE SELECT ALL THAT APPLY.)

☐ No Pain Or Discomfort ☐ It Was Very Painful ☐ Some Bleeding From My Vagina (Not Relating to A Monthly Period) ☐ Urinating Was Painful ☐ I Tried Not to Urinate So That It Did Not Hurt ☐ My Vagina/Lips Became Infected ☐ My Vagina/Lips Were Swollen ☐ A Girl (Other Than Myself) Became Very Ill On Camp ☐ A Girl Died On The Camp

How old were you when this happenned?

◯ 0-2 years ◯ 3-7 years ◯ 7-12 years ◯ 13-18 years ◯ >19 years

---

Please think back to your childhood and answer the following statements with never, sometimes, often or very often

Ke ne ke sa ja dijo tse lekaneng

◯ Ho hang ◯ Ka dinako tse ding ◯ Ha ngata ◯ Kgafetsa

Ke dutse malapeng a fapaneng ka dinako tse fapaneng

◯ Ho hang ◯ Ka dinako tse ding ◯ Ha ngata ◯ Kgafetsa

Ke bone kapa ke utlwetse hore mme wa ka o ne a otlwa ke monna kapa mohlankana wa hae

◯ Ho hang ◯ Ka dinako tse ding ◯ Ha ngata ◯ Kgafetsa

E mong wa ba lelapa la heso o mpolelletse hore ke botswa kapa setlatla kapa ke mobe

◯ Ho hang ◯ Ka dinako tse ding ◯ Ha ngata ◯ Kgafetsa

Ho na le motho ya phollotseng dirope, marao, matswele kapa bokapele ba ka ke sa batle kapa a etsa hore ke tshware ditho tsa hae ke sa batle

◯ Ho hang ◯ Ka dinako tse ding ◯ Ha ngata ◯ Kgafetsa

Ke ile ka rohakwa kapa ka swabiswa ke e mong wa ba lelapa la heso mahlong a batho ba bang

◯ Ho hang ◯ Ka dinako tse ding ◯ Ha ngata ◯ Kgafetsa

Lapeng ke ile ka shapuwa ka lebanta kapa thupa kapa sephadi kapa ho hong ho neng ho le thata

◯ Ho hang ◯ Ka dinako tse ding ◯ Ha ngata ◯ Kgafetsa

Ke entse thobalanong le monna wa dilemo tse fetang tsa ka ka tse ka hodimo ho tse 5

◯ Ho hang ◯ Ka dinako tse ding ◯ Ha ngata ◯ Kgafetsa

Motswadi wa ka a le mong kapa bobedi ba bona ba ne ba tahwa haholo ho ka ntlhokomela

◯ Ho hang ◯ Ka dinako tse ding ◯ Ha ngata ◯ Kgafetsa

Lapeng ke ile ka shapuwa haholo hoo ho setseng mabadi kapa bokgubedu

◯ Ho hang ◯ Ka dinako tse ding ◯ Ha ngata ◯ Kgafetsa

Ke qetile nako ka ntle le lehae mme ha ho motswadi ya neng a tseba hore ke hokae

◯ Ho hang ◯ Ka dinako tse ding ◯ Ha ngata ◯ Kgafetsa

Ke entse thobalano le motho eo e neng e se mohlankana wa ka hobane ke tshoseditswe kapa ke tshositswe kapa ke qebelletswe

◯ Ho hang ◯ Ka dinako tse ding ◯ Ha ngata ◯ Kgafetsa

Na o ile mofuteng ofe kapa ofe wa lebollo kapa ho ya thabeng?

◯ Tjhe ◯ E

Moketeng ona, na o ile wa etsahallwa ke efe kapa efe ya tse latelang?
(PLEASE SELECT ALL THAT APPLYIF SELECTED LABIAL STRETCHING, ASK QUESTIONS C15)

☐ Ke nwele Biya/Dipiriti ☐ Ke fuwe dithethefatsi ☐ Rutilwe ka Boimana le Ho beleha ☐ Rutilwe thuto ya thobalano ☐ Hore monna o kgotsofatswa jwang ☐ Hore diphiri di bolokwa jwang le ho se bue ka mathata ☐ Fuwe lebitso le letjha ☐ Malebe (Labia) kapa Clitoris e sarollotswe kapa ya kgaolwa ☐ Phollotswe ke motho e mong ka tsela e sa amohelehang lebollong ☐ Utlwisitswe bohloko ka thobalano ke motho e mong lebollong ☐ Qobelletswe ho etsa thobalano

O kgethile hore labia kapa clitoris ya hao e sarollotswe kapa e kgaotswe. Ke kopa hore o mpolelle hore na o etsahalletswe ke efe kapa efe ya tse latelang ha sena se etswa kapa ka mora moo (se amanang le sena)
(PLEASE SELECT ALL THAT APPLY.)

☐ Ho ne ho se bohloko ☐ Ho ne ho le bohloko haholo ☐ Kuku ya kae ne e tswa Madi a Mangata (E seng ho amanang le Ho ya Kgweding) ☐ Ho ntsha metsi ho ne ho le Bohloko ☐ Ke Lekile Ho se Ntshe metsi Hore Ho se be Bohloko ☐ Kuku ya ka /Malebe a Tshwaeditswe ☐ Kuku ya ka /Malebe a ne a Ruruhile ☐ Ngwanana e mong (Ntle le nna) o ile a Kula Haholo Kampong ☐ Ngwanana e mong o ile a Hlokahala Kampong

One onale dilemo tsekae ha ketsahalo ena ehohlahella

◯ 0-2 years ◯ 3-7 years ◯ 7-12 years ◯ 13-18 years ◯ >19 years

---

Please think back to your childhood and answer the following statements with never, sometimes, often or very often

Bengingenakho okokudla okwanele

◯ Neze nhlobo ◯ Ngezinye Izikhathi ◯ Kaningi ◯ Kaningi Kakhulu

Ngihlala emizini ehlukene ngezikhathi ezahlukene

◯ Neze nhlobo ◯ Ngezinye Izikhathi ◯ Kaningi ◯ Kaningi Kakhulu

Ngabona noma ngezwa umama eshaywa ngumyeni wakhe noma yisoka

◯ Neze nhlobo ◯ Ngezinye Izikhathi ◯ Kaningi ◯ Kaningi Kakhulu

Ngatshelwa ngomunye umuntu womndeni wami ukuthi ngiyivila, noma ngiyisilima noma ngimubi

◯ Neze nhlobo ◯ Ngezinye Izikhathi ◯ Kaningi ◯ Kaningi Kakhulu

Omunye umuntu wangithinta amathanga, izinqe, amabele noma izitho zangasese ngingathandi ukuthi angithinte noma wangenza ukuthi ngithinte izitho zakhe zangasese ngingafuni

◯ Neze nhlobo ◯ Ngezinye Izikhathi ◯ Kaningi ◯ Kaningi Kakhulu

Ngathukwa noma ngahlanjalazwa ngomunye umuntu emndenini wami phambi kwabanye abantu

◯ Neze nhlobo ◯ Ngezinye Izikhathi ◯ Kaningi ◯ Kaningi Kakhulu

Ekhaya bangishaya ngebhande noma ngenduku noma ngesiswebhu noma ngenye into eyayiqinile

◯ Neze nhlobo ◯ Ngezinye Izikhathi ◯ Kaningi ◯ Kaningi Kakhulu

Ngaya ocansini nendoda eyayindala kunami ngeminyaka emi-5

◯ Neze nhlobo ◯ Ngezinye Izikhathi ◯ Kaningi ◯ Kaningi Kakhulu

Oyedwa noma bobabili abazali bami babedakwe kakhulu ukuthi babenganginakekela

◯ Neze nhlobo ◯ Ngezinye Izikhathi ◯ Kaningi ◯ Kaningi Kakhulu

Ngashaywa kakhulu ekhaya kwaze kwasala umvimbi noma umhuzuko

◯ Neze nhlobo ◯ Ngezinye Izikhathi ◯ Kaningi ◯ Kaningi Kakhulu

Ngachitha isikhathi ngaphandle kwasekhaya futhi akekho owaziyo ekhaya ukuthi ngangikuphi

◯ Neze nhlobo ◯ Ngezinye Izikhathi ◯ Kaningi ◯ Kaningi Kakhulu

Ngaya ocansini nomuntu owayengesilona isoka lami ngoba ngangesatshiswa noma ngangesaba noma ngangiphoqwa

◯ Neze nhlobo ◯ Ngezinye Izikhathi ◯ Kaningi ◯ Kaningi Kakhulu

Kukhona yini uhlobo lwesikole sokuthonjiswa owaya kulo noma waya ehlathini noma entabeni?

◯ Cha ◯ Yebo

Kulo mgubho, wahlangabezana yini nanoma yikuphi kokulandelayo?
(PLEASE SELECT ALL THAT APPLY)

☐ Ngaphuza Ubhiya/Ugologo ☐ Nganikwa izidakamziwa ☐ Ngafundiswa Ngokukhulelwa Nokubeletha ☐ Ngafundiswa Ngemfundo Yocansi ☐ Umthokozisa kanjani Umyeni ☐ Uzigcina kanjani izimfihlo futhi ngingakhulumi ngezinkinga ☐ Unikwe igama elisha ☐ Amalebe (Labia) noma Umsunu (Clitoris) wadonselwa ngaphandle noma wasikwa ☐ Wantintwa ngendlela ephathelene nocansi ngumuntu ovela esikoleni sokuthombisa ☐ Ngalinyazwa ngokocansi ngomunye umuntu esikoleni ☐ Ngaphoqwa ukwenza ucansi

Ukhethe ukuthi amalebe akho noma umsunu wawunwetshiwe noma wawusikiwe. Ngicela ungitshele ukuthi ngabe kukhona yini kokulandelayo owakuzwa ngesikhathi kwenziwa lokhu noma ngemuva kwakho (okuphathelene nalokhu)

(PLEASE SELECT ALL THAT APPLY.)

☐ Akukho Buhlungu noma Ukungaphatheki Kahle ☐ Kwakubuhungu Kakhulu ☐ Ukopha okuncane Okuvela Esithweni Sowesifazane Sangasesen(okungaphathelene Nesikhathi Sangenyanga) ☐ Ukuchama Kwakubuhlungu ☐ Ngazama Ukungachami Ukuze Ngingezulimala ☐ Isitho Sami Sangasese/Izindebe Zami Zatheleleka ☐ Isitho Sami Sangasese/Izindebe Zami Zazivuvukele ☐ Intombazane (Ngaphandle Kwami) Yagula kakhulu Enkanjini ☐ Intombazane Yashona Enkanjini

Ubune minyaka emingakhi ngesikhathi loku kwenziwa

◯ 0-2 years ◯ 3-7 years ◯ 7-12 years ◯ 13-18 years ◯ >19 years

Gendernorms

Are you currently dating a man or woman?
(IF DATING MALE & FEMALE, ASK NEXT QUESTIONS ABOUT MALE PARTNER)

◯ Male ◯ Female ◯ Both ◯ No One

How old is your partner?

____
(IF NO PARTNER, ASK ABOUT MOST RECENT PARTNER)

Does your partner currently do anything to earn money?

◯ No ◯ Yes

Does your partner know that you sell sex?

◯ Yes ◯ No

Does your partner drink alcohol? How often?

◯ Never ◯ Every Day/Nearly Every Day ◯ Only At Weekends ◯ A Few Times in A Month
◯ Less Than Once A Month ◯ I Don'T Know

Have you ever quarrelled or had any other conflict over his/her drinking?

◯ No ◯ Yes

Does your partner smoke dagga or take other drugs? How often?

◯ Never ◯ Every Day/Nearly Every Day ◯ Only At Weekends ◯ A Few Times in A Month
◯ Less Than Once A Month ◯ I Don'T Know

Na ha jwale o intsha le motho wa monna kapa mosadi?

◯ Motho e Motona ◯ Motshehadi ◯ Ha ho na motho

Molekane wa hao o dilemo tse kae?

____
(IF NO PARTNER, ASK ABOUT MOST RECENT PARTNER)

Na ha jwale molekane wa hao ho na le ntho eo a e etsang ho fumana tjhelete?

◯ Tjhe ◯ E

Na molekane wa hao o nwa jwala? Ha kae?

◯ Ho hang ◯ Letsatsi le leng le le leng/Nako e ka etsang Letsatsi le leng le le leng ◯ Mafelo a Beke Feela
◯ Makgetlo a Mmalwa Kgweding ◯ Ka tlase ho Lekgetlo le le leng ka Kgwedi ◯ Ha ke Tsebe

Na le kile la omanyana kapa la ba le ho se dumellane hofe kapa hofe ka ho nwa ha hae?

◯ Tjhe ◯ E

Na molekane wa hao o tsuba matekwane kapa o sebedisa dithethefatsi tse ding? Makgetlo a makae?

◯ Ho hang ◯ Letsatsi le leng le le leng/Nako e ka etsang Letsatsi le leng le le leng ◯ Mafelo a Beke Feela
◯ Makgetlo a Mmalwa Kgweding ◯ Ka tlase ho Lekgetlo le le leng ka Kgwedi ◯ Ha ke Tsebe

Njengamanje uthandana nendoda noma nomuntu wesifazane?

◯ Owesilisa ◯ Owesifazane ◯ Akukho Muntu

Mdala kangakanani umlingani wakho?

____
(IF NO PARTNER, ASK ABOUT MOST RECENT PARTNER)

Ingabe umlingani wakho kukhona akwenzayo ukuthola imali?

◯ Cha ◯ Yebo

Umlingani wakho uyabuphuza utshwala? Uvamise kangakanani?

◯ Neze Nhlobo ◯ Zonke Izinsuku/Cishe Zonke Izinsuku ◯ Ngezimpelasonto Kuphela
◯ Izikhathi Ezimbalwa Ngenyanga ◯ Ngaphansi Kokukodwa Ngenyanga ◯ Angazi

Nike naxabana yini noma naba nayiphi nje enye ingxabano ngokuphuza kwakhe?

◯ Cha ◯ Yebo

Ingabe umlingani wakho uyayibhema insangu noma athathe ezinye izidakamizwa? Uvamise kangakanani?

◯ Neze Nhlobo ◯ Zonke Izinsuku/Cishe Zonke Izinsuku ◯ Ngezimpelasonto Kuphela
◯ Izikhathi Ezimbalwa Ngenyanga ◯ Ngaphansi Kokukodwa Ngenyanga ◯ Angazi

Pregnancy

How old were you when you first had sex?

____

Which of the following statements most closely describes your experiences the first time you had sexual intercourse?

◯ I Was Willing ◯ I Was Pursuaded ◯ I Was Tricked ◯ I Was Forced ◯ I Was Raped

Are you currently doing something or using any method to delay or avoid getting pregnant?
(IF NO, SKIP TO E4)

◯ No ◯ Yes

Which method are you using?

◯ The Injection ◯ Pill ◯ Condoms ◯ The Loop (IUD) ◯ Implant ◯ Herbal or Other Traditional Medicines ◯ Condoms and the Pill or Injection ◯ Withdrawal Before Sperming (Ejactulation)
◯ Other

Have you ever been pregnant?
(IF NO, SKIP TO E9 AND CONFIRM)

◯ No ◯ Yes

How old were you when you first became pregant

____

How many children have you given birth to ?

____

Have any of your children died after birth (this could be immediately afterwards or ever)?
(IF NO, SKIP TO E9 AND CONFIRM)

◯ No ◯ Yes

How many of your children have died?

____

Are you pregnant now?
(IF NO OR UNSURE, SKIP TO E11)

◯ No ◯ Yes ◯ Unsure

How many months pregnant are you?

____

Have you ever had a termination of pregnancy/abortion?
(IF UNSURE, PLEASE CLARIFY WHAT A TOP/ABOTION IS. THEN RE-ASK QUESTION.)

◯ No ◯ Yes ◯

In your lifetime, how many abortionss have you successfully had?

____

O ne o le dilemo tse kae ha o qala ho etsa thobalano?

____

Ke seteitemente sefe ho tse latelang se hlalosang dintho tse o etsahalletseng lekgetlong la ho qala ha o etsa thobalano?

◯ Ke ne ke Batla ◯ Ke ile ka Ferehwa ◯ Ke ile ka Qhekanyetswa ◯ Ke ile ka Qobellwa ◯ Ke ile ka Betwa

Ana ha jwale ho na le ntho eo o e etsang kapa o sebedisa mokgwa ofe kapa ofe ho diehisa kapa ho qoba ho ima?
(IF NO, SKIP TO E4)

◯ Tjhe ◯ E

O sebedisa mokgwa ofe?

◯ O a hlaba ◯ Pidisi ◯ Dikhondomo ◯ Lupu (IUD) ◯ Implant ◯ Ditlama kapa Ditlhare tse ding tsa Setso ◯ Dikhondomo le Pidisi kapa Ho hlaba ◯ O ikgula Pele le Sepema (Rota) ◯ Mokgwa o mong

Na o kile wa ima?
(IF NO, SKIP TO E9 AND CONFIRM)

◯ Tjhe ◯ E

O ne o le dilemo tse kae ha o qala ho ima?

____

O belehile bana ba bakae?

____

Na ho na le ngwana ofe kapa ofe wa bana ba hao ya hlokahetseng ka mora peleho (hona ho ka ba hanghang ka mora moo kapa kgale)?
(IF NO, SKIP TO E9 AND CONFIRM)

◯ Tjhe ◯ E

Bana ba hao ba hlokahetseng ba bakae?

____

Na ha jwale o moimana?
(IF NO OR UNSURE, SKIP TO E11)

◯ Tjhe ◯ E ◯ Ha ke na bonnete

Ke dikgwedi tse kae o le mmeleng?

____

Na o kile wa ntsha mpa?
(IF UNSURE, PLEASE CLARIFY WHAT A TOP/ABOTION IS. THEN RE-ASK QUESTION.)

◯ Tjhe ◯ E

Bophelong ba hao, o bile le katleho ya ho ntsha mpa hakae?

____

Wawungakanani lapho uqala ukuya ocansini?

____

Yisiphi kuzitatimende ezilandelayo ezichaza ngokusondele kakhulu olwazini lwakho okokuqala wenza ucansi?

◯ Ngangifisa ◯ Ngancengwa ◯ Ngaphicwa ◯ Ngaphoqwa ◯ Ngadlwengulwa

Ingabe njengamanje kukhona okusebenzisa noma indlela nanoma iyiphi ukuthi wephuze noma ugweme ukukhulelwa?

◯ Cha ◯ Yebo

Usebenzisa yiphi indlela?

◯ Umjovo ◯ Iphilisi ◯ Amakhondomu ◯ Iluphu (IUD) ◯ Okufakwayo ◯ Amakhambi noma Eminye Imithi Engamakhambi ◯ Amakhondomu kanye nePhilisi noma Umjovo ◯ Ukukhipha Ngaphambi Kokukhipha Isidoda (Ukuchama) ◯ Okunye

Sewake wakhulelwa yini?

◯ Cha ◯ Yebo

Wawungakanani lapho ukhulelwa okokuqala?

____

Zingaki izingane owazibeletha?

____

Ikhona yini ezinganeni zakho eyashona ngemuva kokubelethwa (lokhu kungaba ngokushesha ngemuva noma nje)?

◯ Cha ◯ Yebo

Zingaki ezinganeni zakho ezashona?

____

Ukhulelwe yini manje?

◯ Cha ◯ Yebo ◯ Anginasiqiniseko

Unezinyanga ezingaki ukhulelwe?

____

Sewake wasikhipha isisu?
(IF UNSURE, PLEASE CLARIFY WHAT A TOP/ABOTION IS. THEN RE-ASK QUESTION.)

◯ Cha ◯ Yebo

Empilweni yakho yonke, zingaki izisu owazikhipha ngempumelelo?

____

Violence

Please answer each statement with never, once, a few or many times. These questions are about your main partner, an ex or kwapheni.

Within the past year did any partner insult you or make you feel bad about yourself?
(IF NEVER, ASK 216 AS WELL)

◯ Never ◯ Once ◯ Few ◯ Many

Within the past year did any partner make a show off of you (belittle) or humiliate you in front of other people?
(IF NEVER, ASK 216 AS WELL)

◯ Never ◯ Once ◯ Few ◯ Many

Within the past year did any partner do things to scare or intimidate you on purpose for example by the way he looked at you, by yelling or smashing things?
(IF NEVER, ASK 216 AS WELL)

◯ Never ◯ Once ◯ Few ◯ Many

Within the past year did any partner threaten to hurt you?
(IF NEVER, ASK 216 AS WELL)

◯ Never ◯ Once ◯ Few ◯ Many

More than a year ago did any boyfriend do any of these things to you?

◯ Yes ◯ No

Within the past year did any partner slap you, push you or throw something at you which could hurt you?

◯ Never ◯ Once ◯ Few ◯ Many

Within the past year did any partner hit you with a fist or with something else (such as a beer bottle, stick or belt) which could hurt you?

◯ Never ◯ Once ◯ Few ◯ Many

Within the past year did any partner kick, drag, beat, choke or burn you?

◯ Never ◯ Once ◯ Few ◯ Many

Within the past year did any partner threaten to use or actually use a gun, knife or other weapon against you?

◯ Never ◯ Once ◯ Few ◯ Many

More than a year ago did any partner physically hurt you in any of these ways?

◯ Yes ◯ No

Within the past year did any partner physically force you to have sex when you did not want to?

◯ Never ◯ Once ◯ Few ◯ Many

Within the past year did you have sex with any partner when you did not want to because you were afraid of what he might do?

◯ Never ◯ Once ◯ Few ◯ Many

How many times has this happenned to you in the past 12 months

____

More than a year ago did any partner force you to do anything sexual when you did not want to?

◯ Yes ◯ No

When was the most recent time you were made to have sex with a partner when you did not want it?

____
(IF NEVER, ENTER 01-01-0001)

More than a year ago did any partner make a show off of you (belittle) or humiliate you in front of other people?

◯ Never ◯ Once ◯ Few ◯ Many

More than a year ago did any partner threaten to hurt you?

◯ Never ◯ Once ◯ Few ◯ Many

These questions are about mcimbi

Within the past year did any Mcimbi slap you, push you or throw something at you which could hurt you?

◯ Never ◯ Once ◯ Few ◯ Many

Within the past year did any Mcimbi hit you with a fist or with something else (such as a beer bottle, stick or belt) which could hurt you?

◯ Never ◯ Once ◯ Few ◯ Many

Within the past year did any Mcimbi kick, drag, beat, choke or burn you?

◯ Never ◯ Once ◯ Few ◯ Many

Within the past year did any Mcimbi threaten to use or actually use a gun, knife or other weapon against you?

◯ Never ◯ Once ◯ Few ◯ Many

More than a year ago did any Mcimbi physically hurt you in any of these ways?

◯ Yes ◯ No

Within the past year did any Mcimbi physically force you to have sex when you did not want to?

◯ Never ◯ Once ◯ Few ◯ Many

Within the past year did you have sex with any Mcimbi when you did not want to because you were afraid of what he might do?

◯ Never ◯ Once ◯ Few ◯ Many

How many times has this happenned to you in the past 12 months

____

More than a year ago did any Mcimbi force you to do anything sexual when you did not want to?

◯ Yes ◯ No

When was the most recent time you were made to have sex with an Mcimbi when you did not want it?

____
(IF NEVER, ENTER 01-01-0001)

These questions are about Police

Within the past year did any Police slap you, push you or throw something at you which could hurt you?

◯ Never ◯ Once ◯ Few ◯ Many

Within the past year did any Police hit you with a fist or with something else (such as a beer bottle, stick or belt) which could hurt you?

◯ Never ◯ Once ◯ Few ◯ Many

Within the past year did any Police kick, drag, beat, choke or burn you?

◯ Never ◯ Once ◯ Few ◯ Many

Within the past year did any Police threaten to use or actually use a gun, knife or other weapon against you?

◯ Never ◯ Once ◯ Few ◯ Many

More than a year ago did any Police physically hurt you in any of these ways?

◯ Yes ◯ No

Within the past year did any Police physically force you to have sex when you did not want to?

◯ Never ◯ Once ◯ Few ◯ Many

Within the past year did you have sex with any Police when you did not want to because you were afraid of what he might do?

◯ Never ◯ Once ◯ Few ◯ Many

How many times has this happenned to you in the past 12 months

____

More than a year ago did any Police force you to do anything sexual when you did not want to?

◯ Yes ◯ No

When was the most recent time you were made to have sex with a Policeman when you did not want it?

____
(IF NEVER, ENTER 01-01-0001)

Have you ever experienced gang rape?

◯ Yes ◯ No

How often has this happenned

____

Please answer each statement with never, once, a few or many times.These questions are about your main partner, an ex or kwapheni.

Selemo se fetileng, na ho na le molekane ofe kapa ofe ya o rohakileng kapa a etsa hore o ikutlwe hampe?
(IF NEVER, ASK 216 AS WELL)

◯ Ho hang ◯ Ha nngwe ◯ Makgetlo a Mmalwa ◯ Ha ngata

Selemong se fetileng, na ho na le molekane ofe kapa ofe ya o nyatsitseng kapa a o phoqa ka pela batho ba bang?
(IF NEVER, ASK 216 AS WELL)

◯ Ho hang ◯ Ha nngwe ◯ Makgetlo a Mmalwa ◯ Ha ngata

Selemong se fetileng, na ho na le molekane ofe kapa ofe ya entseng dintho ho o tshosa kapa ho o tshabisa ka boomo, ho etsa mohlala, ka tsela eo a o shebileng ka yona, ka ho o omanya kapa ho thuba dintho?
(IF NEVER, ASK 216 AS WELL)

◯ Ho hang ◯ Ha nngwe ◯ Makgetlo a Mmalwa ◯ Ha ngata

Selemong se fetileng, na ho na le molekane ofe kapa ofe ya tshoseditseng ho o utlwisa bohloko?
(IF NEVER, ASK 216 AS WELL)

◯ Ho hang ◯ Ha nngwe ◯ Makgetlo a Mmalwa ◯ Ha ngata

Nakong e fetang selemo e fetileng, na ho na le mohlankana ofe kapa ofe ya o entseng ntho efe kapa efe ya tse latelang?

◯ E ◯ Tjhe

Selemong se fetileng, na ho na le molekane ofe kapa ofe ya o otlileng, ya o sutuditseng kapa a o lahlella ka ho hong ho neng ho ka o utlwisa bohloko?

◯ Ho hang ◯ Ha nngwe ◯ Makgetlo a Mmalwa ◯ Ha ngata

Selemong se fetileng, na ho na le molekane ofe kapa ofe ya o otlileng ka feisi kapa ka ntho e nngwe (e jwaloka lebotlolo la biya, thupa kapa lebanta), le neng le ka o utlwisa bohloko?

◯ Ho hang ◯ Ha nngwe ◯ Makgetlo a Mmalwa ◯ Ha ngata

Selemong se fetileng, na ho na le molekane ofe kapa ofe ya o rahileng, a o hulanya, a o otla, a o tjhouka kapa a o tjhesa?

◯ Ho hang ◯ Ha nngwe ◯ Makgetlo a Mmalwa ◯ Ha ngata

Selemong se fetileng, na ho na le molekane ofe kapa ofe ya tshoseditseng ho sebedisa kapa ya sebedisitseng sethunya, thipa kapa sebetsa se seng ho wena?

◯ Ho hang ◯ Ha nngwe ◯ Makgetlo a Mmalwa ◯ Ha ngata

Pele ho dikgwedi tse 12 tse fetileng na ho na le molekane ofe kapa ofe ya o utlwisitseng bohloko mmeleng ka tsela efe kapa efe ya tse latelang?

◯ E ◯ Tjhe

Selemo se fetileng, na molekane ofe kapa ofe o o qobelletse ho etsa thobalano ha o ne o sa batle?

◯ Ho hang ◯ Ha nngwe ◯ Makgetlo a Mmalwa ◯ Ha ngata

Selemo se fetileng, na o entse thobalano le molekane ofe kapa ofe ha o ne o sa batle hobane o ne o tshohile seo a ka se etsang?

◯ Ho hang ◯ Ha nngwe ◯ Makgetlo a Mmalwa ◯ Ha ngata

How many times has this happenned to you in the past 12 months

____

Ho feta selemo se se ngwe se fetileng, na ho na le molekane ofe kapa ofe ya o qobelletseng ho etsa ntho efe kapa efe ya thobalano ha o ne o sa batle ho etsa?

◯ E ◯ Tjhe

Lekgetlo le sa tswa feta leo o entsweng hore o etse thobalano le molekane o sa e batle le ne le le neng?

____
(IF NEVER, ENTER 01-01-0001)

Ho feta selemo se se ngwe se fetileng, na ho na le molekane ofe kapa ofe ya o kenyeditseng mahlo (nyatsitseng) kapa a o etsa setshehisa ka pela batho ba bang?

◯ Ho hang ◯ Ha nngwe ◯ Makgetlo a Mmalwa ◯ Ha ngata

Ho feta selemo se se ngwe se fetileng, na ho na le molekane ofe kapa ofe ya tshoseditseng ho o utlwisa bohloko?

◯ Ho hang ◯ Ha nngwe ◯ Makgetlo a Mmalwa ◯ Ha ngata

These questions are about mcimbi

Selemong se fetileng, na ho na le Mcimbi ofe kapa ofe o o otlileng, wa o sututsa kapa wa o lahlella ka ntho e nngwe e neng e ka o utlwisa bohloko?

◯ Ho hang ◯ Ha nngwe ◯ Makgetlo a Mmalwa ◯ Ha ngata

Selemong se fetileng, na ho na le Mcimbi ofe kapa ofe o o otlileng ka feisi kapa ka ntho e nngwe (e jwaloka lebotlolo la biya, thupa kapa lebanta), le neng ho ka o utlwisa bohloko?

◯ Ho hang ◯ Ha nngwe ◯ Makgetlo a Mmalwa ◯ Ha ngata

Selemong se fetileng, na ho na le Mcimbi ofe kapa ofe o o rahileng, wa o hulanya, wa o otla, wa o tjhouka kapa wa o tjhesa?

◯ Ho hang ◯ Ha nngwe ◯ Makgetlo a Mmalwa ◯ Ha ngata

Selemong se fetileng, na ho na le Mcimbi ofe kapa ofe o o tshoseditseng ho sebedisa kapa o sebedisitseng sethunya, thipa kapa sebetsa se seng ho wena?

◯ Ho hang ◯ Ha nngwe ◯ Makgetlo a Mmalwa ◯ Ha ngata

Ho feta selemo se se ngwe se fetileng, na ho na le Mcimbi ofe kapa ofe o o utlwisitseng bohloko mmeleng ka tsela efe kapa efe ya tse latelang?

◯ E ◯ Tjhe

Selemong se fetileng, na Mcimbi ofe kapa ofe o o qobelletse ho etsa thobalano ha o ne o sa batle?

◯ Ho hang ◯ Ha nngwe ◯ Makgetlo a Mmalwa ◯ Ha ngata

Selemong se fetileng, na o entse thobalano le Mcimbi ofe kapa ofe ha o ne o sa batle hobane o ne o tshohile seo a ka se etsang?

◯ Ho hang ◯ Ha nngwe ◯ Makgetlo a Mmalwa ◯ Ha ngata

How many times has this happenned to you in the past 12 months

____

Ho feta selemo se se ngwe se fetileng, na Mcimbi ofe kapa ofe o o qobelletse ho etsa ntho efe kapa efe ya thobalano ha o ne o sa batle ho etsa?

◯ E ◯ Tjhe

Lekgetlo le sa tswa feta leo o entsweng hore o etse thobalano le Mcimbi o sa e batle le ne le le neng?

____
(IF NEVER, ENTER 01-01-0001)

These questions are about Police

Selemong se fetileng, na ho na le Lepolesa lefe kapa lefe le o otlileng, la o sututsa kapa la o lahlella ka ntho e nngwe e neng e ka o utlwisa bohloko?

◯ Ho hang ◯ Ha nngwe ◯ Makgetlo a Mmalwa ◯ Ha ngata

Selemong se fetileng, na ho na le Lepolesa lefe kapa lefe le o otlileng ka feisi kapa ka ntho e nngwe (e jwaloka lebotlolo la biya, thupa kapa lebanta), le neng le ka o utlwisa bohloko?

◯ Ho hang ◯ Ha nngwe ◯ Makgetlo a Mmalwa ◯ Ha ngata

Selemong se fetileng, na ho na le Lepolesa lefe kapa lefe le o rahileng, la o hulanya, la o otla, la o tjhouka kapa la o tjhesa?

◯ Ho hang ◯ Ha nngwe ◯ Makgetlo a Mmalwa ◯ Ha ngata

Selemong se fetileng, na ho na le Lepolesa lefe kapa lefe le o tshoseditseng ho sebedisa kapa le sebedisitseng sethunya, thipa kapa sebetsa se seng ho wena?

◯ Ho hang ◯ Ha nngwe ◯ Makgetlo a Mmalwa ◯ Ha ngata

Ho feta selemo se se ngwe se fetileng, na Lepolesa lefe kapa lefe le o utlwisitseng bohloko mmeleng ka tsela efe kapa efe ya tse latelang?

◯ E ◯ Tjhe

Selemong se fetileng, na Lepolesa lefe kapa lefe le o qobelletse ho etsa thobalano ha o ne o sa batle?

◯ Ho hang ◯ Ha nngwe ◯ Makgetlo a Mmalwa ◯ Ha ngata

Selemong se fetileng, na o entse thobalano le Lepolesa lefe kapa lefe ha o ne o sa batle hobane o ne o tshohile seo le ka se etsang?

◯ Ho hang ◯ Ha nngwe ◯ Makgetlo a Mmalwa ◯ Ha ngata

Sena se o etsahalletse ka makgetlo a makae dikgweding tse 12 tse fetileng?

____

Ho feta selemo se se ngwe se fetileng, na Lepolesa lefe kapa lefe le o qobelletse ho etsa ntho efe kapa efe ya thobalano ha o ne o sa batle ho e etsa?

◯ E ◯ Tjhe

Lekgetlo le sa tswa feta leo o entsweng hore o etse thobalano le Lepolesa o sa e batle le ne le le neng?

____
(IF NEVER, ENTER 01-01-0001)

Na o kile wa betwa ke motho ya fetang a le mong?

◯ E ◯ Tjhe

Sena se etsahetse ka makgetlo a makae?

____

Please answer each statement with never, once, a few or many times.

Onyakeni odlule, kukhona yini umlingani owakuthuka noma wakwenza awangaphatheka kahle ngobuwena uqobo?
(IF NEVER, ASK 216 AS WELL)

◯ Neze nhlobo ◯ kanye ◯ Kambalwa ◯ Kaningi

Onyakeni odlule, kukhona yini umlingani owabukisa ngawe (wakwehlisa isithunzi) noma wakuhlaza phambi kwabanye abantu?
(IF NEVER, ASK 216 AS WELL)

◯ Neze nhlobo ◯ kanye ◯ Kambalwa ◯ Kaningi

Onyakeni odlule, kukhona yini umlingani owenza izinto ukukwesabisa noma ukukwethusa ngenhloso njengendlela akubuka ngayo, ngokuthetha noma ngokuphihliza izinto?
(IF NEVER, ASK 216 AS WELL)

◯ Neze nhlobo ◯ kanye ◯ Kambalwa ◯ Kaningi

Onyakeni odlule, ukhona yini umlingani owake wesabisa ngokukulimaza?
(IF NEVER, ASK 216 AS WELL)

◯ Neze nhlobo ◯ kanye ◯ Kambalwa ◯ Kaningi

Ngaphambi konyaka owodwa odlule, ingabe likhona isoka elenza okunye kwalezi zinto kuwe?

◯ Yebo ◯ Cha

Onyakeni odlule, ingabe ukhona yini umlingani owakushaya ngempama, owakufuqa noma owakujikijela ngento eyayingahle ikulimaze?

◯ Neze nhlobo ◯ kanye ◯ Kambalwa ◯ Kaningi

Onyakeni odlule, ingabe ukhona yini umlingani owakushaya ngesibhakela noma ngenye into (enjengebhodlela likabhiya, induku noma ibhande) eyayingahle ikulimaze?

◯ Neze nhlobo ◯ kanye ◯ Kambalwa ◯ Kaningi

Onyakeni odlule, ingabe ukhona yini umlingani owakukhahlela, owakuhudula, owakushaya, owakuklinya noma owakushisa?

◯ Neze nhlobo ◯ kanye ◯ Kambalwa ◯ Kaningi

Onyakeni odlule, ingabe ukhona yini umlingani owakwesabisa noma owasebenzisa isibhamu, ummese noma esinye isikhali kuwe ngempela?

◯ Neze nhlobo ◯ kanye ◯ Kambalwa ◯ Kaningi

Ngaphambi konyaka owodwa odlule, ingabe ukhona yini umlingani owakulimaza ngokomzimba nganoma iyiphi yalezi zindlela?

◯ Yebo ◯ Cha

Onyakeni odlule, ingabe ukhona yini umlingani owakuphoqa ngokomzimba ukuthi uye ocansini wena ungafuni?

◯ Neze nhlobo ◯ kanye ◯ Kambalwa ◯ Kaningi

Onyakeni odlule, ukhona yini umlingani owaya naye ocansini ungafuni ngenxa yokuthi wawesaba ayengahle akwenze?

◯ Neze nhlobo ◯ kanye ◯ Kambalwa ◯ Kaningi

How many times has this happenned to you in the past 12 months

____

Ngaphambi konyaka owodwa odlule, ukhona yini umlingani owakuphoqa ukuthi wenze nanoma yini ephathelene nocansi ungafuni?

◯ Yebo ◯ Cha

Yisiphi isikhathi esisandukudlula kakhulu owenziwe ukuthi uye ocansini nomlingani ungalufuni

____
(IF NEVER, ENTER 01-01-0001)

Ngaphambi konyaka owodwa odlule, ukhona yini umlingani owabukisa ngawe (wakwehlisa isithunzi) noma wakuhlaza phambi kwabanye abantu?

◯ Neze nhlobo ◯ kanye ◯ Kambalwa ◯ Kaningi

Ngaphambi konyaka owodwa odlule, ukhona yini umlingani owesabisa ngokukulimaza?

◯ Neze nhlobo ◯ kanye ◯ Kambalwa ◯ Kaningi

These questions are about mcimbi

Onyakeni odlule, kukhona yini uMcimbi okushaye ngempama, owakufuqa noma wakujikijela ngento eyayingahle ikulimaze?

◯ Neze nhlobo ◯ kanye ◯ Kambalwa ◯ Kaningi

Onyakeni odlule, ukhona yini uMcimbi owakushaya ngesibhakela noma ngenye into (efana nebhodlela likabhiya, induku noma ibhande) eyayingahle ikulimaze?

◯ Neze nhlobo ◯ kanye ◯ Kambalwa ◯ Kaningi

Onyakeni odlule, ingabe ukhona yini uMcimbi owakukhahlela, owakuhudula, owakushaya, owakuklinya noma owakushisa?

◯ Neze nhlobo ◯ kanye ◯ Kambalwa ◯ Kaningi

Onyakeni odlule, ukhona yini uMcimbi owesabisa ngokusebenzisa noma owasebenzisa isibhamu, ummese noma esinye isikhali ngempela kuwe?

◯ Neze nhlobo ◯ kanye ◯ Kambalwa ◯ Kaningi

Ngaphambi konyaka owodwa odlule, ukhona yini uMcimbi owakulimaza ngokomzimba ngananoma iyiphi kulezi zindlela?

◯ Yebo ◯ Cha

Onyakeni odlule, ukhona yini uMcimbi owakuphoqa ngokomzimba ukuthi uye ocansini ungafuni?

◯ Neze nhlobo ◯ kanye ◯ Kambalwa ◯ Kaningi

Onyakeni odlule, ukhona yini uMcimbi owaya naye ocansini ungafuni ngenxa yokuthi wawesaba owawungahle ukwenze?

◯ Neze nhlobo ◯ kanye ◯ Kambalwa ◯ Kaningi

How many times has this happenned to you in the past 12 months

____

Ngaphambi konyaka owodwa odlule, ukhona yini uMcimbi owakuphoqa ukuthi wenze nanoma yini ephathelene nocansi wena ungafuni?

◯ Yebo ◯ Cha

Yisiphi isikhathi esisandakudlula kakhulu lapho owenziwa khona ukuthi uye ocansini noMcimbi wena ungalufuni?

____
(IF NEVER, ENTER 01-01-0001)

These questions are about Police

Onyakeni odlule, likhona yini iPhoyisa elakushaya ngempama, lakufuqa noma lakujikijela ngento eyayingahle ikulimaze?

◯ Neze nhlobo ◯ kanye ◯ Kambalwa ◯ Kaningi

Onyakeni odlule, likhona yini iPhoyisa elikushaye ngesibhakela noma ngenye into (efana nebhodlela, induku noma ibhande) okungahle kukulimaze?

◯ Neze nhlobo ◯ kanye ◯ Kambalwa ◯ Kaningi

Onyakeni odlule, likhona yini iPhoyisa elakukhahlela, elakuhudula, elakushaya, elakuklinya noma elakushisa?

◯ Neze nhlobo ◯ kanye ◯ Kambalwa ◯ Kaningi

Ezinyangeni eziyi-12 ezedlule, likhona yini iPhoyisa elasabisa ngokusebenzisa noma elasebenzisa isibhamu, ummese noma esinye isikhali ngempela kuwe?

◯ Neze nhlobo ◯ kanye ◯ Kambalwa ◯ Kaningi

Ngaphambi konyaka owodwa odlule, likhona yini iPhoyisa elakulimaza ngokomzimba ngananoma iyiphi kulezi zindlela?

◯ Yebo ◯ Cha

Onyakeni odlule, likhona yini iPhoyisa elikuphoqe ngokomzimba ukuthi uye nalo ocansini wena ungafuni?

◯ Neze nhlobo ◯ kanye ◯ Kambalwa ◯ Kaningi

Onyakeni odlule, kukhona yini iPhoyisa nanoma yiliphi oye nalo ocansini ungafuni ngenxa yokwesaba elingahle likwenze?

◯ Neze nhlobo ◯ kanye ◯ Kambalwa ◯ Kaningi

Onyakeni odlule, kukhona yini iPhoyisa elikuphoqe ukuthi wenze ucansi ngomlomo nalo wena ungafuni?

◯ Neze nhlobo ◯ kanye ◯ Kambalwa ◯ Kaningi

Onyakeni odlule, kukhona yini iPhoyisa elikuphoqe ukuthi wenze ucansi lwasendunu nalo wena ungafuni?

◯ Neze nhlobo ◯ kanye ◯ Kambalwa ◯ Kaningi

Yisiphi isikhathi esisandakudlula kakhulu lapho owenziwa khona ukuthi uye ocansini nePhoyisa wena ungalufuni?

____
(IF NEVER, ENTER 01-01-0001)

Kwenzeke izikhathi eziningi kangakanani lokhu ezinyangeni ezi-12 ezedlule?

____

Ngaphambi konyaka owodwa odlule, likhona yini iPhoyisa elakuphoqa ukuthi wenze nanoma yini ephathelene nocansi wena ungafuni?

◯ Yebo ◯ Cha

Uke wahlangabezana nokudlwengulwa abantu abame umugqa?

◯ Yebo ◯ Cha

Kwenzeke kaningi kangakanani lokhu

____

Sex

When was the last time you had sex (normal (vagina)/anal/oral)?

____

The last time you had sex did you use a condom?
(IF NO, SKIP TO 278)

◯ Yes ◯ No

Did you use a condom for every round (a round is until he cums/ejaculates/sperms)?

◯ Yes ◯ No

Who brought the condom, was it you or your partner?

◯ Self ◯ Partner ◯ Both

Did you experience any of the following with the condom?

☐ Condom Breaking/Busting ☐ Condom Slipping Off ☐ Condom Only Put On Half Way
☐ Condom Was Removed During Sex ☐ No Problems,Condom Worked Perfectly

The last time you had sex, who was it with (a main partner, kwhapheni, mcimbi or nonpaying one night stand or ex-partner)?

◯ Regter/Main Partner/Vat 'N Sit ◯ Makhwapheni ◯ Mcimbi ◯ Ex Partner ◯ One Night Stand

Have you used condoms with your MAIN PARTNER in the past month? Would you say you used them always, often or sometimes?

◯ Not Used ◯ Always ◯ Often ◯ Sometimes

How many MAIN PARTNERS have you had sex with in the last year?

____

How many KHWAPHENI have had sex with during the past year?

____

How many one night stands have you had sex with during the past year?

____

Over the last year how often have you experienced the condom breaking or slipping off or only put it on half way through or have you taken it off and continued love making?

◯ Every Time Used ◯ Often ◯ Sometimes ◯ Never ◯ No Use

In the past 6 months have you experienced any of the folowing symptoms?
(IF NO SYMPTOMS EXPERIENCED, SKIP TO G14)

☐ Painful or Burning Vagina when Urinate ☐ Sores or Boils Around Vagina ☐ Itching Vagina
☐ Abnormal Vaginal Discharge ☐ Pain in The Bottom Of Your Stomach (Not Related To Your Menstrual Period Or Using The Loop) ☐ None Of These Symptoms

Did you receive treatment for the symptoms you just mentioned?

◯ Yes ◯ No

Have you ever been diagnosed with a sexually transmitted disease such as gonorrhea, syphilis, chlamydia, genital warts, or genital herpes

◯ Yes ◯ No ◯ Unsure ◯ Refused To Answer

Please select all of the different services that you offer

☐ Talking Or Companionship in Person ☐ Massage ☐ Dinner ☐ Breast Flashing ☐ Over The Phone Sex (Talking) ☐ Whatsapp/Sms Sex ☐ Internet/Email/Online Sex ☐ Normal Sex (vagina)
☐ Anal Sex ☐ blow job (oral sex) ☐ Hand Job ☐ Stripping Or Dancing ☐ Group Sex
☐ Other ☐

Please select the one services that is your most popular (that most mcimbi ask for)

◯ Talking Or Companionship in Person ◯ Massage ◯ Dinner ◯ Breast Flashing ◯ Over The Phone Sex (Talking) ◯ Whatsapp/Sms Sex ◯ Internet/Email/Online Sex ◯ Normal Sex (vagina)
◯ Anal Sex ◯ blow job (oral sex) ◯ Hand Job ◯ Stripping Or Dancing ◯ Group Sex
◯ Other ◯

In the past week, how many days did you have oral,anal or Normal sex (vagina) with mcimbi

____

In the past month, how many days did you work have oral, anal or Normal sex (vagina) with mcimbi

____
(MUST BE MORE THAN PREVIOUS)

How many one-time mcimbi did you have sex with in the last working DAY?

____

How many one-time mcimbi did you have sex with in the past WEEK?

____
(MUST BE MORE THAN PREVIOUS)

The last time you had sex with one-time mcimbi did you go with a condom?

◯ Without Condom ◯ Male Condom ◯ Female Condom ◯ Male and Female Condom

Do you always go with a condom with your one-time mcimbi?

◯ Every Time Used ◯ Often ◯ Sometimes ◯ Never ◯ No Use

How many of your regulars did you have sex with in the last working day?

____

How many of your regulars did you have sex with in the past week?

____
(MUST BE MORE THAN PREVIOUS)

The last time you had sex with regular mcimbi did you go with a condom?

◯ Without (Condom) ◯ Male Condom ◯ Female Condom ◯ Male and Female Condom

Has there been any occasion in the past month when either a regular or a one-time Mcimbi went without a condom for any reason when you were having sex?

◯ Yes ◯ No ◯ Refused Answer

Do you always work in the same place?

◯ Yes ◯ No

How many different places did you practise sex work in the past 6 months? (different taverns and different street corners, etc)

____

Have you ever worked as a sex worker in another province in South Africa

◯ Yes ◯ No

Have you ever worked as a sex worker in another country, not South Africa?

◯ Yes ◯ No

How much were you paid the last time you had Normal sex (vagina)?

____
(number)

How much did you earn the last day that you sold sex?

____
(number)

What is the maximum amount that you earned in 1 day this last month end (month end)

____
(number)

What is the minimum amount that you earned in 1 day this last month (mid month)

____
(number)

In the last month have you used anything to dry, clean or tighten your vagina before having sex? Have you done this every time, some times, just once or not at all?
(IF NEVER, SKIP TO G36)

◯ Everytime ◯ Sometimes ◯ Once ◯ Never

What have you used?
(PLEASE MARK ALL THAT APPLY)

☐ Soap ☐ Herbs/Snuff ☐ Ice/Water ☐ Zamlandela/Tiger Balm ☐ Alone Stone ☐ Brown Paper
☐ Ming Balls ☐ Other

In the last month have you used anything to lubricate your vagina before having sex? Have you done this every time, some times, just once or not at all?
(IF NEVER, SKIP TO G38)

◯ Everytime ◯ Sometimes ◯ Once ◯ Never

What have you used?
(PLEASE MARK ALL THAT APPLY)

☐ Lubricant ☐ Soap ☐ Spit ☐ Vaseline ☐ Baby Oil ☐ Other

In the last month have you used anything to hide that you were bleeding/mensruating, while having sex? Have you done this every time you bled, some times when you bled, just once or not at all?
(IF NEVER, SKIP TO G40)

◯ Everytime ◯ Sometimes ◯ Once ◯ Never

What have you used?
(PLEASE MARK ALL THAT APPLY)

☐ Tampon ☐ Kitchen Sponge ☐ Cloth ☐ Cotton Wool ☐ Toilet Paper ☐ Nacosa Condom
☐ Brown paper ☐ Other

Please answer each of the following statements with never, once, a few times or often.

Within the past year have you been verbally insulted, harassed or threatenned because you are a sex worker?

◯ Never ◯ Once ◯ A Few Times ◯ Often

Within the past year have you been beaten or threatenned because you are a sex worker?

◯ Never ◯ Once ◯ A Few Times ◯ Often

Within the past year have you experienced sexual abuse because you are a sex worker?

◯ Never ◯ Once ◯ A Few Times ◯ Often

Within the past year have any of your family (partner, children, siblings etc) experienced discrimination as a result of your being a sex worker?

◯ Never ◯ Once ◯ A Few Times ◯ Often

Within the past year how often have you been forced to change where you live because you are a sex worker?

◯ Never ◯ Once ◯ A Few Times ◯ Often

Within the past year have you been denied health services because you are a sex worker?

◯ Never ◯ Once ◯ A Few Times ◯ Often

Within the past year have you been denied police assistance/services because you are a sex worker?

◯ Never ◯ Once ◯ A Few Times ◯ Often

Please answer each of the following statements with strongly agree, agree, disagree or strongly disagree

Within the past year I have felt ashamed because I am a sex worker?

○ Strongly Agree ○ Agree ○ Disagree ○ Strongly Disagree

Within the past year I have felt guilty because I am a sex worker?

○ Strongly Agree ○ Agree ○ Disagree ○ Strongly Disagree

Within the past year I have blamed myself because I am a sex worker?

○ Strongly Agree ○ Agree ○ Disagree ○ Strongly Disagree

Within the past year I have blamed others because I am a sex worker?

○ Strongly Agree ○ Agree ○ Disagree ○ Strongly Disagree

Within the past year I have felt that I am not worth anything because I am a sex worker?

○ Strongly Agree ○ Agree ○ Disagree ○ Strongly Disagree

Within the past year I have felt punished because I am a sex worker?

○ Strongly Agree ○ Agree ○ Disagree ○ Strongly Disagree

Within the past year I have felt suicidal because I am a sex worker?

○ Strongly Agree ○ Agree ○ Disagree ○ Strongly Disagree

Within the past year I have chosen not to attend a party/bashes because of being a sex worker?

○ Strongly Agree ○ Agree ○ Disagree ○ Strongly Disagree

Within the past year I have isolated myself from friends and family because of being a sex worker?

○ Strongly Agree ○ Agree ○ Disagree ○ Strongly Disagree

Within the past year I have decided to not have (any more) children because of being a sex worker?

○ Strongly Agree ○ Agree ○ Disagree ○ Strongly Disagree

Within the past year I have avoided going to the clinic/hospital because of being a sex worker?

○ Strongly Agree ○ Agree ○ Disagree ○ Strongly Disagree

Within the past year I have avoided going to the police because of being a sex worker?

○ Strongly Agree ○ Agree ○ Disagree ○ Strongly Disagree

O qetetse neng ho etsa thobalano (ka kukung/ka maraong/ka molomo)?

____

Lekgetlong la ho qetela ha o etsa thobalano na o ile wa sebedisa khondomo?
(IF NO, SKIP TO 278)

○ E ○ Tjhe

Na o sebedisitse khondomo lekgetlong le leng le le leng (round ke ho fihlela a rota)?

◯ E ◯ Tjhe

Ke mang ya tlileng le khondomo, na ke wena kapa molekane wa hao?

◯ Ke itletse le yona ◯ Molekane ◯ Bobedi ba rona

Na o bile le ketsahalo efe kapa efe ya tse latelang ka khondomo?

☐ Khondomo e phunyehang/phatlohang ☐ Khondomo e Tswang ☐ Khondomo e Kentsweng Halofo
☐ Khondomo e Tlositswe nakong ya Thobalano ☐ Ha ho a ba le Mathata,Khondomo e sebeditse Hantle

Lekgetlong la ho qetela ha o etsa thobalano, o ne o etsa le mang (regte, makhwapheni, mcimbi kapa motho ya sa lefeng eo o etsang le yena bosiu bo le bong feela kapa eo e neng e le molekane)

◯ Regte/Molekane wa Bohlokwa/Vat 'N Sit ◯ Makhwapheni ◯ Mcimbi ◯ Eo e neng e le Molekane
◯ Wa bosiu bo le bong

Na o sebedisitse dikhondomo le MOLEKANE wa hao wa NNETE kgweding e fetileng? Na o ka re le di sebedisitse ka mehla, kgafetsa kapa ka dinako tse ding?

◯ Ha re a di Sebedisa ◯ Ka mehla ◯ Kgafetsa ◯ Ka dinako tse ding

O entse thobalano le boregte ba bakae selemong se sa tswa feta?

____

O entse thobalano le bomakhwapheni ba bakae selemong se fetileng?

____

O entse thobalano le balekane ba bakae ba bosiu bo le bong feela selemong se fetileng?

____

Dilemong tse ka pele ho tse sa tswa feta, ke makgetlo a makae o bileng le ketsahalo ya ho phunyeha ha khondomo kapa ho tswa ha yona kapa ho e kenya halofo feela kapa ho e ntsha le ho tswela pele ho etsa thobalano?

◯ Re e Sebedisitse Nako e nngwe le e nngwe ◯ Kgafetsa ◯ Ka dinako tse ding ◯ Ho hang
◯ Ha e a sebetsa

Dikgweding tse 6 tse fetileng na o etsahalletswe ke efe kapa efe ya tse latelang?
(IF NO SYMPTOMS EXPERIENCED, SKIP TO G14)

☐ Setho sa Bosadi se Bohloko kapa se Tjhesang ha o Rota ☐ Diso tse Potapotileng Kuku
☐ Kuku e Hlohlonang ☐ Lero le Sa Tlwaelehang le tswang Kukung ☐ Lehlaba ka Tlasa Mpa (E seng le Amanang le Ho ya Kgweding kapa Ho sebedisa Lupu) ☐ Ha ho a ba le Matshwao ana

Na o ile wa fumana kalafo ya matshwao ao o sa tswa a bolela?

◯ E ◯ Tjhe

Na o kile wa hlahlojwa o na le lefu le fetiswang ka thobalano le jwalo ka gonorrhea, syphilis, chlamydia, genital warts kapa genital herpes?

◯ E ◯ Tjhe ◯ Ha ke na bonnete ◯ Hanne ho Araba

Ka kopo kgetha ditshebeletso kaofela tseo o fanang ka tsona

☐ Ho bua kapa Botswalle ☐ Masaje ☐ Dijo tsa Mantsiboya ☐ Breast Flashing ☐ Thobalano ya Mohaleng (Ho bua) ☐ Thobalano ya Whatsapp/Sms ☐ Thobalano ya Inthaneteng/Imeile/Online ☐ Thobalano ka Kukung ☐ Thobalano ka Maraong ☐ Thobalano ka Molomo ☐ Hand Job ☐ Ho hlobola kapa Ho tantsha ☐ Thobalano ya Sehlopha ☐ E nngwe ☐

Ka kopo kgetha tshebeletso eo o tummeng ka yona (eo boholo ba mcimbi bo e kopang)

○ Ho bua kapa Bo Tshwale le Motho ○ Masaje ○ Dijo tsa Mantsiboya ○ Breast Flashing ○ Thobalano ya Mohaleng (Ho bua) ○ Thobalano ya Whatsapp/Sms ○ Thobalano ya Inthaneteng/Imeile/Online ○ Thobalano ka Kukung ○ Thobalano ka Maraong ○ Thobalano ka Molomo ○ Hand Job ○ Ho hlobola kapa Ho tantsha ○ Thobalano ya Sehlopha ○ E nngwe ○

Bekeng e fetileng, o entse thobalano ya molomo, ya ka maraong kapa ka kukung le mcimbi ka matsatsi a makae?

____

Kgweding e fetileng, mosebetsi wa hao o bile le thobalano ya molomo, ya ka maraong kapa ka kukung le mcimbi ka matsatsi a makae?

____
(MUST BE MORE THAN PREVIOUS)

LETSATSING le qetelang la mosebetsi o entse thobalano le bomcimbi ba bakae ba tlang ha nngwe feela?

____

BEKENG e fetileng o entse thobalano le bomcimbi ba bakae ba tlang ha nngwe feela?

____
(MUST BE MORE THAN PREVIOUS)

Lekgetlong le fetileng o etsa thobalano le mcimbi ya tlang ha nngwe feela le entse ka khondomo?

○ Ntle le Khondomo ○ Khondomo ya Banna ○ Khondomo ya Basadi ○ Khondomo ya Banna le ya Basadi

Na ka mehla le etsa ntle le khondomo le mcimbi ya tlang ha nngwe feela?

○ Re e Sebedisitse Nako e nngwe le e nngwe ○ Kgafetsa ○ Ka dinako tse ding ○ Ho hang ○ Ha e a sebetsa

O entse thobalano le batho ba hao ba bakae ba tlwaelehileng letsatsing le qetelang la ho sebetsa?

____

O entse thobalano le batho ba hao ba bakae ba tlwaelehileng bekeng e fetileng?

____
(MUST BE MORE THAN PREVIOUS)

Lekgetlong le sa tswa feta ha o ne o etsa thobalano le mcimbi wa hao o tlwaelehileng, na le entse ka khondomo?

○ Ntle le Khondomo ○ Khondomo ya Banna ○ Khondomo ya Basadi ○ Khondomo ya Banna le ya Basadi

Na ho bile le ketsahalo efe kapa efe kgweding e fetileng, ha motho eo o tlwaetseng ho robala le yena kapa Mcimbi ya tlang ha nngwe feela a kena ntle le khondomo ka lebaka lefe kapa lefe ha le etsa thobalano?

◯ E ◯ Tjhe ◯ Hanne ho araba

Na o sebeletsa sebakeng se le seng kamehla?

◯ E ◯ Tjhe

Dibaka tse fapaneng tseo o etseditseng thobalano ho tsona dikgweding tse 6 tse fetileng di kae? (dithavene tse fapaneng le dihuku tsa diterata tse fapaneng)

____

Na o kile wa sebetsa jwalo ka sex worker porovenseng e nngwe Aforika Borwa?

◯ E ◯ Tjhe

Na o kile wa sebetsa jwalo ka sex worker naheng e nngwe eo e seng Aforika Borwa?

◯ E ◯ Tjhe

O lefilwe bokae lekgetlong la ho qetela ha o ne o etsa thobalano e Tlwaelehileng (ya ka kukung)?

____
(number)

O entse bokae letsatsing la ho qetela leo o rekisitseng thobalano ka lona?

____
(number)

Okare boholo batjhelete eo oethotseng kgwedi efetileng kebokae

____
(number)

Okare bonyane batjhelete eo oethotseng kgwedi efetileng kebokae

____
(number)

Kgweding e sa tswa feta na o sebedisitse ntho efe kapa efe ya ho omisa, ho hlwekisa kapa ho tiisa kuku ya hao pele o etsa thobalano? Na o entse sena lekgetlo le leng le le leng, ka dinako tse ding, ha nngwe feela kapa ho hang?
(IF NEVER, SKIP TO G36)

◯ Lekgetlo le leng le le leng ◯ Ka dinako tse ding ◯ Ha nngwe ◯ Ho hang

O sebedisitse eng?
(PLEASE MARK ALL THAT APPLY)

☐ Sesepa ☐ Ditlama/Seneifi ☐ Aese/Metsi ☐ Zamlandela/Tiger Balm ☐ Alone Stone
☐ Pampiri e Sootho ☐ Ming Balls ☐ E nngwe

Kgweding e sa tswa feta na o sebedisitse ntho efe kapa efe ya ho loetsa kuku ya hao pele o etsa thobalano? Na o entse sena lekgetlo le leng le le leng, ka dinako tse ding, ha nngwe feela kapa ho hang?
(IF NEVER, SKIP TO G38)

◯ Lekgetlo le leng le le leng ◯ Ka dinako tse ding ◯ Ha nngwe ◯ Ho hang

O sebedisitse eng?
(PLEASE MARK ALL THAT APPLY)

☐ Seloetsi ☐ Sesepa ☐ Mathe ☐ Vaseline ☐ Oli ya Maseya ☐ E nngwe

Kgweding e sa tswa feta na o sebedisitse ntho efe kapa efe ho pata hore o ne o le kgweding ha o etsa thobalano? Na o entse sena lekgetlo le leng le le leng ha o tswa madi, ha nngwe feela kapa ho hang?
(IF NEVER, SKIP TO G40)

◯ Lekgetlo le leng le le leng ◯ Ka dinako tse ding ◯ Ha nngwe ◯ Ho hang

O sebedisitse eng?
(PLEASE MARK ALL THAT APPLY)

☐ Themphone ☐ Sepontjhe sa ka Kitjhini ☐ Lesela ☐ Khothene Wulu ☐ Pampiri ya Ntlwaneng
☐ Khondomo ya Nacosa ☐ E nngwe ☐ Brown paper

Please answer each of the following statements with never, once, a few times or often.

Selemong se fetileng, na o rohakilwe, wa hlekefetswa le ho tshosetswa ka molomo ka lebaka la ho ba sex worker?

◯ Ho hang ◯ Ha nngwe ◯ Makgetlo a Mmalwa ◯ Kgafetsa

Selemong se fetileng na o ile wa otlwa kapa wa tshosetswa ka lebaka la ho ba sex worker?

◯ Ho hang ◯ Ha nngwe ◯ Makgetlo a Mmalwa ◯ Kgafetsa

Selemong se fetileng, na o ile wa ba le ketsahalo ya ho hlekefetswa ka thobalano ka lebaka la ho ba sex worker?

◯ Ho hang ◯ Ha nngwe ◯ Makgetlo a Mmalwa ◯ Kgafetsa

Selemong se fetileng, na ho na le motho ofe kapa ofe wa lelapa la heno (molekane, bana, bao o tswalanang le bona, jj) ya bileng le ketsahalo ya ho kgethollwa ka lebaka la hobane wena o le sex worker?

◯ Ho hang ◯ Ha nngwe ◯ Makgetlo a Mmalwa ◯ Kgafetsa

Selemong se fetileng, ke makgetlo a makae o qobellehileng ho fetola sebaka sa hao sa bodulo ka lebaka la ho ba sex worker?

◯ Ho hang ◯ Ha nngwe ◯ Makgetlo a Mmalwa ◯ Kgafetsa

Selemong se fetileng na o ile wa hanelwa ditshebeletso tsa bophelo hobane o le sex worker?

◯ Ho hang ◯ Ha nngwe ◯ Makgetlo a Mmalwa ◯ Kgafetsa

Selemong se fetileng, na o ile wa hanelwa thuso/ditshebeletso tsa sepolesa hobane o le sex worker?

◯ Ho hang ◯ Ha nngwe ◯ Makgetlo a Mmalwa ◯ Kgafetsa

Please answer each of the following statements with strongly agree, agree, disagree or strongly disagree

Selemong se fetileng, ke ikutlwile ke itshwabela hobane ke sex worker?

◯ Ke Dumela Ke Tiile ◯ Ke a Dumela ◯ Ke a Hana ◯ Ke Hana ke Tiile

Selemong se fetileng, ke ipone molato hobane ke sex worker?

◯ Ke Dumela Ke Tiile ◯ Ke a Dumela ◯ Ke a Hana ◯ Ke Hana ke Tiile

Selemong se fetileng, ke itshwaya phoso hobane ke sex worker?

◯ Ke Dumela Ke Tiile ◯ Ke a Dumela ◯ Ke a Hana ◯ Ke Hana ke Tiile

Selemong se fetileng, ke tshwaile ba bang diphoso hobane ke sex worker?

◯ Ke Dumela Ke Tiile ◯ Ke a Dumela ◯ Ke a Hana ◯ Ke Hana ke Tiile

Selemong se fetileng, ke ikutlwile eka ha ke bohlokwa ka ntho efe kapa efe hobane ke sex worker?

◯ Ke Dumela Ke Tiile ◯ Ke a Dumela ◯ Ke a Hana ◯ Ke Hana ke Tiile

Selemong se fetileng, ke ipone ke fuwa kotlo hobane ke sex worker?

◯ Ke Dumela Ke Tiile ◯ Ke a Dumela ◯ Ke a Hana ◯ Ke Hana ke Tiile

Selemong se fetileng ke utlwile eka nka ipolaya hobane ke sex worker?

◯ Ke Dumela Ke Tiile ◯ Ke a Dumela ◯ Ke a Hana ◯ Ke Hana ke Tiile

Selemong se fetileng, ke kgethile ho se ye phathing/dibesheng ka lebaka la ho ba sex worker?

◯ Ke Dumela Ke Tiile ◯ Ke a Dumela ◯ Ke a Hana ◯ Ke Hana ke Tiile

Selemong se fetileng ke ikgutse metswalleng le ho ba leloko ka lebaka la ho ba sex worker?

◯ Ke Dumela Ke Tiile ◯ Ke a Dumela ◯ Ke a Hana ◯ Ke Hana ke Tiile

Selemong se fetileng, ke entse qeto ya ho se (hlole ke) be le bana ka lebaka la ho ba sex worker?

◯ Ke Dumela Ke Tiile ◯ Ke a Dumela ◯ Ke a Hana ◯ Ke Hana ke Tiile

Selemong se fetileng, ke qobile ho ya tleleniking/sepetlele ka lebaka la ho ba sex worker?

◯ Ke Dumela Ke Tiile ◯ Ke a Dumela ◯ Ke a Hana ◯ Ke Hana ke Tiile

Selemong se fetileng, ke qobile ho ya sepoleseng ka lebaka la ho ba sex worker?

◯ Ke Dumela Ke Tiile ◯ Ke a Dumela ◯ Ke a Hana ◯ Ke Hana ke Tiile

Wagcina nini ukuya ocansini (olwesitho sangasese somuntu wesifazane/olwasendunu/olomlomo)?

____

Ngesikhathi sokugcina uya ocansini wayisebenzisa yini ikhondomu?
(IF NO, SKIP TO 278)

◯ Yebo ◯ Cha

Wasebenzisa ikhondomu kumjikelezo ngamunye (umjikelezo uze ufike lapho echama khona/ekhipha khona isidoda)?

◯ Yebo ◯ Cha

Ubani oweza nekhondomu, kwakunguwe noma umlingani wakho?

◯ Mina uqobo ◯ Umlingani ◯ Kokubili

Kukhona kukokulandelayo owakuzwa ngekhondomu?

☐ Ukudabuka/Ukuqhuma Kwekhondomu ☐ Ukumonyuka Kwekhondomu ☐ Ikhondomu Lifakwe Uhhafu
☐ Ikhondomu Lakhishwa Ngesikhathi Socansi ☐ Akunazinkinga, Ikhondomu Lisebenza Kahle Kakhulu

Ngesikhathi sokugcina uya ocansini, wawulwenza nobani (umaqondana, umakhwapheni, umcimbi noma owobusuku obubodwa ongakhokhi noma umlingani wesikhathi esedlule)?

◯ Umaqondana/Umlingani Wangempela/Umkipito ◯ UMakhwapheni ◯ UMcimbi ◯ Umlingani Wesikhathi Esedlule ◯ Okulalwe Naye Ubusuku Obubodwa

Uke wawasebenzisa amakhondomu noMAQONDANA enyangeni eyedlule? Ungasho yini ukuthi niwasebenzise njalo, kaningi noma ngezinye izikhathi?

◯ Awasetshenziswanga ◯ Njalo ◯ Kaningi ◯ Ngezinye izikhathi

Bangaki omaqondana oye nabo ocansini ngonyaka odlule?

____

Bangaki omakhwapheni oye nabo ocansini ngonyaka odlule?

____

Bangaki oye nabo ocansini ubusuku obubodwa ngonyaka odlule?

____

Onyakeni odlule kwenzeke kaningi kangakanani ukuthi udatshukelwe yikhondomu noma imonyuke iphume noma uyifaka uhhafu kuphela noma uyikhiphe bese uqhubeka nocansi?

◯ Izikhathi Zonke Lisetshenziswa ◯ Kaningi ◯ Ngezinye Izikhathi ◯ Akaze Nhlobo
◯ Akusetshenziswa

Ezinyangeni eziyi-6 ezedlule uke waba nazo nanoma yiziphi kulezi zimpawu ezilandelayo?
(IF NO SYMPTOMS EXPERIENCED, SKIP TO G14)

☐ Ubuhlungu noma Ukushisa Komchamo lapho Ngichama ☐ Izilonda noma Amathumba Azungeze Isitho Sangasese ☐ Isitho Sangasese Esilumayo ☐ Okuphuma Esithweni Sangasese Okungajwayelekile
☐ Ubuhlungu Ezansi Nesisu Sakho (Akuphathelene Nesikhathi Senyanga Noma Nokusebenzisa Iluphu)
☐ Akukho kulezi Zimpawu

Wakuthola ukwelashelwa izimpawu osandukuzisho?

◯ Yebo ◯ Cha

Wake waxilongwa watholakala unesifo esidluliswa ngocansi esifana negonoriya, i-syphilis, i-chlamydia, izinsumpa esithweni sokuzala, noma i-herpes ezithweni zokuzala?

◯ Yebo ◯ Cha ◯ Anginasiqiniseko ◯ Walile Ukuphendula

Sicela ukhethe yonke eyemisebenzi yokusiza ehlukene eniyinikezayo

☐ Ukukhuluma noma Ubungane Nomuntu ☐ Ukumasaja ☐ Idina ☐ Ukukhipha Ubisi Ebeleni
☐ Ucansi Olwenziwa Ngocingo (Ukukhuluma) ☐ Ucansi nge-Whatsapp/nge-Sms ☐ Inthanethi/Emeyili/Ucans i nge-Inthanethi ☐ Ucansi Lwesitho sangasese Sowesifazane ☐ Ucansi Lwasendunu ☐ Ucansi Lomlomo
☐ Ukwenza Ngesandla ☐ Ukukhumula noma Ukudansa ☐ Ucansi Lweqembu ☐ Okunye
☐

Sicela ukhethe owodwa wemisebenzi yokusiza edume kakhulu kini (umcimbi omningi kakhulu oyicelayo)

◯ Ukukhuluma noma Ubungane Nomuntu ◯ Ukumasaja ◯ Idina ◯ Ukukhipha Ubisi Ebeleni
◯ Ucansi Olwenziwa Ngocingo (Ukukhuluma) ◯ Ucansi nge-Whatsapp/nge-Sms ◯ Inthanethi/Emeyili/Ucans i nge-Inthanethi ◯ Ucansi Lwesitho sangasese Sowesifazane ◯ Ucansi Lwasendunu ◯ Ucansi Lomlomo
◯ Ukwenza Ngesandla ◯ Ukukhumula noma Ukudansa ◯ Ucansi Lweqembu ◯ Okunye

Esontweni eledlule, zingaki izinsuku owenze ngazo nomcimbi ucansi lomlomo, lwasendunu noma olwesitho sangasese somuntu wesifazane?

____

Enyangeni edlule, zingaki izinsuku osebenze ngazo wenza nomcimbi ucansi lomlomo, lwasendunu noma olwesitho sangasese somuntu wesifazane?

____
(MUST BE MORE THAN PREVIOUS)

Mingaki imicimbi yesikhathi esisodwa owenze nayo ucansi ngoSUKU lokugcina lokusebenza?

____

Mingaki imicimbi yesikhathi esisodwa owenze nayo ucansi ngeSONTO eledlule?

____
(MUST BE MORE THAN PREVIOUS)

Ngesikhathi sokugcina uya ocansini nomcimbi wesikhathi esisodwa wahamba nekhondomu yini?

◯ Ngaphandle Kwekhondomu ◯ Ikhondomu Yowesilisa ◯ Ikhondomu Yowesifazane
◯ Ikhondomu Yowesilisa kanye neYowesifazane

Njalo uhamba nekhondomu nomcimbi wakho wesikhathi esisodwa?

◯ Izikhathi Zonke Lisetshenziswa ◯ Kaningi ◯ Ngezinye Izikhathi ◯ Akaze Nhlobo
◯ Akusetshenziswa

Bangaki abakho abavamile owenze nabo ucansi ngosuku lokugcina lokusebenza?

____

Bangaki abakho abavamile owenze nabo ucansi ngesonto eledlule?

____
(MUST BE MORE THAN PREVIOUS)

Ngesikhathi sokugcina uya ocansini nomcimbi ovamile wahamba nekhondomu yini?

◯ Ngaphandle (Kwekhondomu) ◯ Ikhondomu Yowesilisa ◯ Ikhondomu Yowesifazane
◯ Ikhondomu Yowesilisa kanye neYowesifazane

Ingabe kukhona yini isigameko lapho esontweni eledlule lapho kungaba uMcimbi ovamile noma owesikhathi esisodwa uhambe ngaphandle kwekhondomu ngenxa yananoma yisiphi isizathu lapho nanenza ucansi?

◯ Yebo ◯ Cha ◯ Walile Ukuphendula

Njalo usebenza endaweni efanayo?

◯ Yebo ◯ Cha

Zingaki izindawo ezahlukene owenze kuzo umsebenzi wocansi ezinyangeni eziyi-6 ezedlule? (amathaveni ahlukene kanye namakhona ezitaladi ahlukene, njll.)

____

Wake wasebenza njenge-sex worker kwesinye isifundazwe eNingizimu Afrika

◯ Yebo ◯ Cha

Wake wasebenza njenge-sex worker kwelinye izwe, hhayi eNingizimu Afrika?

◯ Yebo ◯ Cha

Wakhokhelwa malini ngesikhathi sokugcina lapho wenza khona ucansi oluVamile (ngesitho sangasese sowesifazane)?

____
(number)

Wathola malini ngosuku lokugcina owathengisa ngalo ucansi?

____
(number)

Kwinyanga eyedlule ingaba buyimalini ubukhulu bemali oyenzile

____
(number)

Enyangeni edlule makungekhobhinzi uthola malini

____
(number)

Enyangeni eyedlule kukhona oyisebenzisile ukuthi womise, uhlanze noma uqinise isitho sakho sangasese ngaphambi kokuthi uye ocansini? Lokhu ukwenze ngaso sonke isikhathi, ngezinye izikhathi, kanye nje noma awukaze ukwenze nhlobo?
(IF NEVER, SKIP TO G36)

◯ Ngezikhathi Zonke ◯ Ngezinye izikhathi ◯ Kanye ◯ Neze nhlobo

Usebenziseni?
(PLEASE MARK ALL THAT APPLY)

☐ Insipho ☐ Amakhambi/Isinemfu ☐ I-ayisi/Amanzi ☐ Zamlandela/I-Tiger Balm ☐ Wedwa Itshe
☐ Iphepha Elinsundu ☐ Amabhola Asezimayini ☐ Okunye

Enyangeni eyedlule kukhona into oyisebenzisile ukuthambisa isitho sakho sangasese ngaphambi kokuthi uye ocansini? Lokhu ukwenze ngaso sonke isikhathi, ngezinye izikhathi, kanye nje noma awukaze ukwenze nhlobo?
(IF NEVER, SKIP TO G38)

◯ Ngezikhathi Zonke ◯ Ngezinye izikhathi ◯ Kanye ◯ Neze nhlobo

Usebenziseni?
(PLEASE MARK ALL THAT APPLY)

☐ Isithambisi ☐ Insipho ☐ Amathe ☐ uVaseline ☐ i-Baby Oil ☐ Okunye

Enyangeni eyedlule kukhona into oyisebenzisile ukuthi ufihle ukuthi bewopha/bewusesikhathini, ngesikhathi wenza ucansi? Lokhu ukwenze ngaso sonke isikhathi wopha, ngezinye izikhathi uma wopha, kanye nje noma awukaze ukwenze nhlobo?
(IF NEVER, SKIP TO G40)

◯ Ngezikhathi Zonke ◯ Ngezinye izikhathi ◯ Kanye ◯ Neze nhlobo

Usebenziseni?
(PLEASE MARK ALL THAT APPLY)

☐ iTampon ☐ Isipontshi Sasekhishini ☐ Indwangu ☐ i-Cotton Wool ☐ Iphepha Lasethoyilethi
☐ I-Nacosa Khondomu ☐ Brown paper ☐ Okunye

Please answer each of the following statements with never, once, a few times or often.

Onyakeni odlule, uke wethukwa ngomlomo, wahlushwa noma wesatshiswa ngenxa yokuba yi-sex worker?

◯ Neze nhlobo ◯ Kanye ◯ Izikhathi Ezimbalwa ◯ Kaningi

Onyakeni odlule, uke washaywa noma wesatshiswa ngenxa yokuba yi-sex worker?

◯ Neze nhlobo ◯ Kanye ◯ Izikhathi Ezimbalwa ◯ Kaningi

Onyakeni odlule, uke wahlukunyezwa ngokocansi ngenxa yokuba yi-sex worker?

◯ Neze nhlobo ◯ Kanye ◯ Izikhathi Ezimbalwa ◯ Kaningi

Onyakeni odlule, ukhona yini owomndeni wakho (umlingani, izingane, ozalwa nabo, njll) oke wabandlululwa ngenxa yokuba kwakho yi-sex worker?

◯ Neze nhlobo ◯ Kanye ◯ Izikhathi Ezimbalwa ◯ Kaningi

Onyakeni odlule, kukaningi kangakanani uphoqwa ukuba ushintshe indawo yakho ohlala kuyo ngenxa yokuba yi-sex worker?

◯ Neze nhlobo ◯ Kanye ◯ Izikhathi Ezimbalwa ◯ Kaningi

Onyakeni odlule, uke wancishwa kangaki izinsizakalo zezempilo ngoba uyi-sex worker?

◯ Neze nhlobo ◯ Kanye ◯ Izikhathi Ezimbalwa ◯ Kaningi

Onyakeni odlule, uke walelwa yini usizo izinsizakalo zamaphoyisa ngoba uyi-sex worker?

◯ Neze nhlobo ◯ Kanye ◯ Izikhathi Ezimbalwa ◯ Kaningi

Please answer each of the following statements with strongly agree, agree, disagree or strongly disagree

Onyakeni odlule, ngizizwe nginamahlobni ngoba ngiyi-sex worker?

◯ Ngiyavuma Kakhulu ◯ Ngiyavuma ◯ Angivumi ◯ Angivumi Kakhulu

Onyakeni odlule, ngizizwe nginecala ngoba ngiyi-sex worker?

◯ Ngiyavuma Kakhulu ◯ Ngiyavuma ◯ Angivumi ◯ Angivumi Kakhulu

Onyakeni odlule, ngizisolile ngoba ngiyi-sex worker?

◯ Ngiyavuma Kakhulu ◯ Ngiyavuma ◯ Angivumi ◯ Angivumi Kakhulu

Onyakeni odlule, ngisole abanye ngoba ngiyi-sex worker?

◯ Ngiyavuma Kakhulu ◯ Ngiyavuma ◯ Angivumi ◯ Angivumi Kakhulu

Onyakeni odlule, ngizizwe ukuthi angifanelwe yilutho ngoba ngiyi-sex worker?

◯ Ngiyavuma Kakhulu ◯ Ngiyavuma ◯ Angivumi ◯ Angivumi Kakhulu

Onyakeni odlule, ngizizwe ngijeziswa ngoba ngiyi-sex worker?

◯ Ngiyavuma Kakhulu ◯ Ngiyavuma ◯ Angivumi ◯ Angivumi Kakhulu

Onyakeni odlule, ngizizwe ngifuna ukuzibulala ngoba ngiyi-sex worker?

◯ Ngiyavuma Kakhulu ◯ Ngiyavuma ◯ Angivumi ◯ Angivumi Kakhulu

Onyakeni odlule, uke ngikhethe ukungayi eMihlanganweni Yomphakathi ngenxa yokuba yi-sex worker?

◯ Ngiyavuma Kakhulu ◯ Ngiyavuma ◯ Angivumi ◯ Angivumi Kakhulu

Onyakeni odlule, ngizahlukanisile kubangane nomndeni ngenxa yokuba yi-sex worker?

◯ Ngiyavuma Kakhulu ◯ Ngiyavuma ◯ Angivumi ◯ Angivumi Kakhulu

Onyakeni odlule, nginqume ukuthi ngingabi (nezihlobo) ngenxa yokuba yi-sex worker?

◯ Ngiyavuma Kakhulu
◯ Ngiyavuma
◯ Angivumi
◯ Angivumi Kakhulu

Onyakeni odlule, ngikugwemile ukuya emtholampilo/esibhedlela ngenxa yokuba yi-sex worker?

◯ Ngiyavuma Kakhulu
◯ Ngiyavuma
◯ Angivumi
◯ Angivumi Kakhulu

Onyakeni odlule, ngikugwemile ukuya emaphoyiseni ngenxa yokuba yi-sex worker?

◯ Ngiyavuma Kakhulu
◯ Ngiyavuma
◯ Angivumi
◯ Angivumi Kakhulu

Mental Health

I would like to ask you some questions about how you have been feeling in the past week. I am going to read out some statements and ask you to say how many days you have had particular feelings or ideas or whether you have not had them at all.

During the past week I was worried by things that usually don't worry me

○ Rarely Or None Of The Time ○ Some Of A Little Of The Time (1-2Days) ○ A Moderate Amount Of Time (2-4 Days) ○ Most Of The Time (5-7Days)

During the past week I did not feel like eating, my appetite was poor

○ Rarely Or None Of The Time ○ Some Of A Little Of The Time (1-2Days) ○ A Moderate Amount Of Time (2-4 Days) ○ Most Of The Time (5-7Days)

During the past week I felt I could not make myself feel happy even with the help of family and friends

○ Rarely Or None Of The Time ○ Some Of A Little Of The Time (1-2Days) ○ A Moderate Amount Of Time (2-4 Days) ○ Most Of The Time (5-7Days)

During the past week I felt I was just as good as other people

○ Rarely Or None Of The Time ○ Some Of A Little Of The Time (1-2Days) ○ A Moderate Amount Of Time (2-4 Days) ○ Most Of The Time (5-7Days)

During the past week I had trouble keeping my mind on what I was doing

○ Rarely Or None Of The Time ○ Some Of A Little Of The Time (1-2Days) ○ A Moderate Amount Of Time (2-4 Days) ○ Most Of The Time (5-7Days)

During the past week I felt depressed (very sad all of the time)

○ Rarely Or None Of The Time ○ Some Of A Little Of The Time (1-2Days) ○ A Moderate Amount Of Time (2-4 Days) ○ Most Of The Time (5-7Days)

During the past week I felt that everything I did was an effort

○ Rarely Or None Of The Time ○ Some Of A Little Of The Time (1-2Days) ○ A Moderate Amount Of Time (2-4 Days) ○ Most Of The Time (5-7Days)

During the past week I felt hopeful about the future

○ Rarely Or None Of The Time ○ Some Of A Little Of The Time (1-2Days) ○ A Moderate Amount Of Time (2-4 Days) ○ Most Of The Time (5-7Days)

During the past week I thought my life had been a failure

○ Rarely Or None Of The Time ○ Some Of A Little Of The Time (1-2Days) ○ A Moderate Amount Of Time (2-4 Days) ○ Most Of The Time (5-7Days)

During the past week I felt fearful

○ Rarely Or None Of The Time ○ Some Of A Little Of The Time (1-2Days) ○ A Moderate Amount Of Time (2-4 Days) ○ Most Of The Time (5-7Days)

During the past week my sleep was restless (woke up in the night/did not sleep well/had trouble falling asleep)

◯ Rarely Or None Of The Time ◯ Some Of A Little Of The Time (1-2Days) ◯ A Moderate Amount Of Time (2-4 Days) ◯ Most Of The Time (5-7Days)

During the past week I was happy

◯ Rarely Or None Of The Time ◯ Some Of A Little Of The Time (1-2Days) ◯ A Moderate Amount Of Time (2-4 Days) ◯ Most Of The Time (5-7Days)

During the past week I talked less than usual

◯ Rarely Or None Of The Time ◯ Some Of A Little Of The Time (1-2Days) ◯ A Moderate Amount Of Time (2-4 Days) ◯ Most Of The Time (5-7Days)

During the past week I felt lonely

◯ Rarely Or None Of The Time ◯ Some Of A Little Of The Time (1-2Days) ◯ A Moderate Amount Of Time (2-4 Days) ◯ Most Of The Time (5-7Days)

During the past week people were unfriendly

◯ Rarely Or None Of The Time ◯ Some Of A Little Of The Time (1-2Days) ◯ A Moderate Amount Of Time (2-4 Days) ◯ Most Of The Time (5-7Days)

During the past week I enjoyed life

◯ Rarely Or None Of The Time ◯ Some Of A Little Of The Time (1-2Days) ◯ A Moderate Amount Of Time (2-4 Days) ◯ Most Of The Time (5-7Days)

During the past week I cried a lot

◯ Rarely Or None Of The Time ◯ Some Of A Little Of The Time (1-2Days) ◯ A Moderate Amount Of Time (2-4 Days) ◯ Most Of The Time (5-7Days)

During the past week I felt sick

◯ Rarely Or None Of The Time ◯ Some Of A Little Of The Time (1-2Days) ◯ A Moderate Amount Of Time (2-4 Days) ◯ Most Of The Time (5-7Days)

During the past week I felt that people dislike me

◯ Rarely Or None Of The Time ◯ Some Of A Little Of The Time (1-2Days) ◯ A Moderate Amount Of Time (2-4 Days) ◯ Most Of The Time (5-7Days)

During the past week I could not get 'going'

◯ Rarely Or None Of The Time ◯ Some Of A Little Of The Time (1-2Days) ◯ A Moderate Amount Of Time (2-4 Days) ◯ Most Of The Time (5-7Days)

During the past week I have been thinking too much

◯ Rarely Or None Of The Time ◯ Some Of A Little Of The Time (1-2Days) ◯ A Moderate Amount Of Time (2-4 Days) ◯ Most Of The Time (5-7Days)

During the past week my heart has been painful

◯ Rarely Or None Of The Time ◯ Some Of A Little Of The Time (1-2Days) ◯ A Moderate Amount Of Time (2-4 Days) ◯ Most Of The Time (5-7Days)

During the past week my spirit has been low

◯ Rarely Or None Of The Time ◯ Some Of A Little Of The Time (1-2Days) ◯ A Moderate Amount Of Time (2-4 Days) ◯ Most Of The Time (5-7Days)

Now I want to ask you a question about the past month (four weeks), Has the thought of ending your life been in your mind?

◯ No ◯ Yes

The following are symptoms that people sometimes experience after having a terrible event happen in their lives. I'll read each one, please state how much the symptom has bothered you in the past week

Recurrent thoughts or memories of the event

◯ Not At All ◯ Rarely ◯ Sometimes ◯ Most Of The Time

Feelings as though the event is happening again

◯ Not At All ◯ Rarely ◯ Sometimes ◯ Most Of The Time

Recurrent nightmares about the event

◯ Not At All ◯ Rarely ◯ Sometimes ◯ Most Of The Time

Sudden emotional or physical reactions when reminded of the event

◯ Not At All ◯ Rarely ◯ Sometimes ◯ Most Of The Time

Avoiding activities that remind you of the event

◯ Not At All ◯ Rarely ◯ Sometimes ◯ Most Of The Time

Avoiding thoughts or feelings associated with the event, so I try not to feel or think about anything that reminds me of it

◯ Not At All ◯ Rarely ◯ Sometimes ◯ Most Of The Time

Feeling jumpy, get a fright easily

◯ Not At All ◯ Rarely ◯ Sometimes ◯ Most Of The Time

Feeling on guard, ready to fight to protect yourself

◯ Not At All ◯ Rarely ◯ Sometimes ◯ Most Of The Time

In the past year, have you cut, burned or hurt yourself on purpose

◯ Yes ◯ No

In the past year, have you attempted suicide?

◯ Yes ◯ No

I would like to ask you some questions about how you have been feeling in the past week. I am going to read out some statements and ask you to say how many days you have had particular feelings or ideas or whether you have not had them at all.

Bekeng e fetileng ke ne ke tshwenngwa ke dintho tse sa ntshwenyeng ka mehla

○ Ha se ha ngata Kapa Ha ho aetsahala ○ Nakonyana e itseng (Letsatsi le le 1-2) ○ Nako e Lekaneng (Matsatsi a 2-4) ○ Boholo ba Nako (Matsatsi a 5-7)

Bekeng e fetileng ke ne ke sa ikutlwe ho ja, ke se na takatso ya dijo

○ Ha se ha ngata Kapa Ha ho aetsahala ○ Nakonyana e itseng (Letsatsi le le 1-2) ○ Nako e Lekaneng (Matsatsi a 2-4) ○ Boholo ba Nako (Matsatsi a 5-7)

Bekeng e fetileng ke ikutlwile ke sa kgone ho thaba le ka thuso ya ba lelapa le metswalle

○ Ha se ha ngata Kapa Ha ho aetsahala ○ Nakonyana e itseng (Letsatsi le le 1-2) ○ Nako e Lekaneng (Matsatsi a 2-4) ○ Boholo ba Nako (Matsatsi a 5-7)

Bekeng e fetileng ke ikutlwile ke lokile jwalo ka batho ba bang

○ Ha se ha ngata Kapa Ha ho aetsahala ○ Nakonyana e itseng (Letsatsi le le 1-2) ○ Nako e Lekaneng (Matsatsi a 2-4) ○ Boholo ba Nako (Matsatsi a 5-7)

Bekeng e fetileng ke bile le bothata ba ho tsepamisa monahano wa ka ho seo ke neng ke se etsa

○ Ha se ha ngata Kapa Ha ho aetsahala ○ Nakonyana e itseng (Letsatsi le le 1-2) ○ Nako e Lekaneng (Matsatsi a 2-4) ○ Boholo ba Nako (Matsatsi a 5-7)

Bekeng e fetileng ke ikutlwile ke sithabetse maikutlo (ke hloname nako kaofela)

○ Ha se ha ngata Kapa Ha ho aetsahala ○ Nakonyana e itseng (Letsatsi le le 1-2) ○ Nako e Lekaneng (Matsatsi a 2-4) ○ Boholo ba Nako (Matsatsi a 5-7)

Bekeng e fetileng ke utlwile eka ntho e nngwe le e nngwe eo ke e etsang e ne e le ho leka

○ Ha se ha ngata Kapa Ha ho aetsahala ○ Nakonyana e itseng (Letsatsi le le 1-2) ○ Nako e Lekaneng (Matsatsi a 2-4) ○ Boholo ba Nako (Matsatsi a 5-7)

Bekeng e fetileng ke ikutlwile ke na le tshepo bokamosong

○ Ha se ha ngata Kapa Ha ho aetsahala ○ Nakonyana e itseng (Letsatsi le le 1-2) ○ Nako e Lekaneng (Matsatsi a 2-4) ○ Boholo ba Nako (Matsatsi a 5-7)

Bekeng e fetileng ke ne ke nahana hore bophelo ba ka ha bo a atleha

○ Ha se ha ngata Kapa Ha ho aetsahala ○ Nakonyana e itseng (Letsatsi le le 1-2) ○ Nako e Lekaneng (Matsatsi a 2-4) ○ Boholo ba Nako (Matsatsi a 5-7)

Bekeng e fetileng ke ikutlwile ke na le letswalo

○ Ha se ha ngata Kapa Ha ho aetsahala ○ Nakonyana e itseng (Letsatsi le le 1-2) ○ Nako e Lekaneng (Matsatsi a 2-4) ○ Boholo ba Nako (Matsatsi a 5-7)

Bekeng e fetileng ke ne ke hlobaela (ke tsoha bosiu/ke sa robale hantle/ ke na le bothata ba ho tlelwa ke boroko

○ Ha se ha ngata Kapa Ha ho aetsahala ○ Nakonyana e itseng (Letsatsi le le 1-2) ○ Nako e Lekaneng (Matsatsi a 2-4) ○ Boholo ba Nako (Matsatsi a 5-7)

Bekeng e fetileng ke ne ke thabile

◯ Ha se ha ngata Kapa Ha ho aetsahala ◯ Nakonyana e itseng (Letsatsi le le 1-2) ◯ Nako e Lekaneng (Matsatsi a 2-4) ◯ Boholo ba Nako (Matsatsi a 5-7)

Bekeng e fetileng ke buile ha nyane ho feta ka tlwaelo

◯ Ha se ha ngata Kapa Ha ho aetsahala ◯ Nakonyana e itseng (Letsatsi le le 1-2) ◯ Nako e Lekaneng (Matsatsi a 2-4) ◯ Boholo ba Nako (Matsatsi a 5-7)

Bekeng e fetileng ke ikutlwile eka ke mong

◯ Ha se ha ngata Kapa Ha ho aetsahala ◯ Nakonyana e itseng (Letsatsi le le 1-2) ◯ Nako e Lekaneng (Matsatsi a 2-4) ◯ Boholo ba Nako (Matsatsi a 5-7)

Bekeng e fetileng batho ba ne ba se mosa

◯ Ha se ha ngata Kapa Ha ho aetsahala ◯ Nakonyana e itseng (Letsatsi le le 1-2) ◯ Nako e Lekaneng (Matsatsi a 2-4) ◯ Boholo ba Nako (Matsatsi a 5-7)

Bekeng e fetileng ke natefetswe ke bophelo

◯ Ha se ha ngata Kapa Ha ho aetsahala ◯ Nakonyana e itseng (Letsatsi le le 1-2) ◯ Nako e Lekaneng (Matsatsi a 2-4) ◯ Boholo ba Nako (Matsatsi a 5-7)

Bekeng e fetileng ke llile haholo

◯ Ha se ha ngata Kapa Ha ho aetsahala ◯ Nakonyana e itseng (Letsatsi le le 1-2) ◯ Nako e Lekaneng (Matsatsi a 2-4) ◯ Boholo ba Nako (Matsatsi a 5-7)

Bekeng e fetileng ke ikutlwile ke sa phela hantle

◯ Ha se ha ngata Kapa Ha ho aetsahala ◯ Nakonyana e itseng (Letsatsi le le 1-2) ◯ Nako e Lekaneng (Matsatsi a 2-4) ◯ Boholo ba Nako (Matsatsi a 5-7)

Bekeng e fetileng ke ikutlwile eka batho ha ba nthate

◯ Ha se ha ngata Kapa Ha ho aetsahala ◯ Nakonyana e itseng (Letsatsi le le 1-2) ◯ Nako e Lekaneng (Matsatsi a 2-4) ◯ Boholo ba Nako (Matsatsi a 5-7)

Bekeng e fetileng ke ne ke sa kgone ho etsa letho

◯ Ha se ha ngata Kapa Ha ho aetsahala ◯ Nakonyana e itseng (Letsatsi le le 1-2) ◯ Nako e Lekaneng (Matsatsi a 2-4) ◯ Boholo ba Nako (Matsatsi a 5-7)

Bekeng e fetileng ke ne ke nahana haholo

◯ Ha se ha ngata Kapa Ha ho aetsahala ◯ Nakonyana e itseng (Letsatsi le le 1-2) ◯ Nako e Lekaneng (Matsatsi a 2-4) ◯ Boholo ba Nako (Matsatsi a 5-7)

Bekeng e fetileng pelo ya ka e ne e le bohloko

◯ Ha se ha ngata Kapa Ha ho aetsahala ◯ Nakonyana e itseng (Letsatsi le le 1-2) ◯ Nako e Lekaneng (Matsatsi a 2-4) ◯ Boholo ba Nako (Matsatsi a 5-7)

Bekeng e fetileng moya wa ka o ne o le tlase

◯ Ha se ha ngata Kapa Ha ho aetsahala ◯ Nakonyana e itseng (Letsatsi le le 1-2) ◯ Nako e Lekaneng (Matsatsi a 2-4) ◯ Boholo ba Nako (Matsatsi a 5-7)

Jwale ke batla ho o botsa potso ka kgwedi e fetileng (dibeke tse nne), Na o bile le monahano wa ho ipolaya?

◯ Tjhe ◯ E

The following are symptoms that people sometimes experience after having a terrible event happen in their lives. I'll read each one, please state how much the symptom has bothered you in the past week

Menahano e ipoeletsang kapa sehopotso sa ketsahalo

◯ Ho hang ◯ Ha se ha ngata ◯ Ka dinako tse ding ◯ Boholo ba Nako

Ho ikutlwa eka ketsahalo e etsahala hape

◯ Ho hang ◯ Ha se ha ngata ◯ Ka dinako tse ding ◯ Boholo ba Nako

Ditoro tse hlobaetsang ka ketsahalo

◯ Ho hang ◯ Ha se ha ngata ◯ Ka dinako tse ding ◯ Boholo ba Nako

Diketso tse seng di bonahala tsa maikutlo kapa mmeleng ha o hopotswa ka ketsahalo

◯ Ho hang ◯ Ha se ha ngata ◯ Ka dinako tse ding ◯ Boholo ba Nako

Qoba diketso tse o hopotsang ka ketsahalo

◯ Ho hang ◯ Ha se ha ngata ◯ Ka dinako tse ding ◯ Boholo ba Nako

Ho qoba menahano kapa maikutlo a amanang le ketsahalo, ka hoo ke leka ho se utlwe kapa ho se nahane ka ntho efe kapa efe e nkgopotsang ka yona

◯ Ho hang ◯ Ha se ha ngata ◯ Ka dinako tse ding ◯ Boholo ba Nako

Ho se dudisehe, ho tshoha habonolo

◯ Ho hang ◯ Ha se ha ngata ◯ Ka dinako tse ding ◯ Boholo ba Nako

Ho ikutlwa o dula o lalletse, o loketse ho lwana ho itshireletsa

◯ Ho hang ◯ Ha se ha ngata ◯ Ka dinako tse ding ◯ Boholo ba Nako

Selemong se fetileng, na o ile wa itsheha, wa itjhesa kapa wa ikutlwisa bohloko ka boomo?

◯ E ◯ Tjhe

Selemong se fetileng, na o ile wa leka ho ipolaya?

◯ E ◯ Tjhe

I would like to ask you some questions about how you have been feeling in the past week. I am going to read out some statements and ask you to say how many days you have had particular feelings or ideas or whether you have not had them at all.

Ngesonto eledlule bengikhathazwa yizinto ezingangikhathazi ngokuvamile

◯ Akujwayele Noma Asikho Isikhathi ◯ Esinye Sesikhathi Esincane (1-2 Izinsuku) ◯ Inani Lesikhathi Elikahle (2-4 Izinsuku) ◯ Isikhathi Esiningi Kakhulu (5-7 Izinsuku)

Ngesonto eledlule bengingathandi ukudla, uthando lwami lokudla belulubi

◯ Akujwayele Noma Asikho Isikhathi ◯ Esinye Sesikhathi Esincane (1-2 Izinsuku) ◯ Inani Lesikhathi Elikahle (2-4 Izinsuku) ◯ Isikhathi Esiningi Kakhulu (5-7 Izinsuku)

Ngesonto eledlule ngizizwe ngingakwazi ukuzijabulisa ngisho nangosizo lomndeni nabangane

◯ Akujwayele Noma Asikho Isikhathi ◯ Esinye Sesikhathi Esincane (1-2 Izinsuku) ◯ Inani Lesikhathi Elikahle (2-4 Izinsuku) ◯ Isikhathi Esiningi Kakhulu (5-7 Izinsuku)

Ngesonto eledlule ngizizwe sengathi bengilungile njengabanye abantu

◯ Akujwayele Noma Asikho Isikhathi
◯ Esinye Sesikhathi Esincane (1-2 Izinsuku)
◯ Inani Lesikhathi Elikahle (2-4 Izinsuku)
◯ Isikhathi Esiningi Kakhulu (5-7 Izinsuku)

Ngesonto eledlule ngibe nenkinga yokugcina umqondo wami kulokhu engangikwenza

◯ Akujwayele Noma Asikho Isikhathi
◯ Esinye Sesikhathi Esincane (1-2 Izinsuku)
◯ Inani Lesikhathi Elikahle (2-4 Izinsuku)
◯ Isikhathi Esiningi Kakhulu (5-7 Izinsuku)

Ngesonto eledule ngazizwa ngidangele (ngidumele kakhulu ngaso sonke isikhathi)

◯ Akujwayele Noma Asikho Isikhathi
◯ Esinye Sesikhathi Esincane (1-2 Izinsuku)
◯ Inani Lesikhathi Elikahle (2-4 Izinsuku)
◯ Isikhathi Esiningi Kakhulu (5-7 Izinsuku)

Ngesonto eledlule ngazizwa sengathi yonke into engangiyenza kwakuwukuzama okukhulu

◯ Akujwayele Noma Asikho Isikhathi ◯ Esinye Sesikhathi Esincane (1-2 Izinsuku) ◯ Inani Lesikhathi Elikahle (2-4 Izinsuku) ◯ Isikhathi Esiningi Kakhulu (5-7 Izinsuku)

Ngesonto eledlule ngazizwa nginethemba ngekusasa

◯ Akujwayele Noma Asikho Isikhathi ◯ Esinye Sesikhathi Esincane (1-2 Izinsuku) ◯ Inani Lesikhathi Elikahle (2-4 Izinsuku) ◯ Isikhathi Esiningi Kakhulu (5-7 Izinsuku)

Ngesonto eledlule ngacabanga ukuthi ngangehlulekile empilweni yami

◯ Akujwayele Noma Asikho Isikhathi ◯ Esinye Sesikhathi Esincane (1-2 Izinsuku) ◯ Inani Lesikhathi Elikahle (2-4 Izinsuku) ◯ Isikhathi Esiningi Kakhulu (5-7 Izinsuku)

Ngesonto eledlule ngazizwa ngesaba

◯ Akujwayele Noma Asikho Isikhathi
◯ Esinye Sesikhathi Esincane (1-2 Izinsuku)
◯ Inani Lesikhathi Elikahle (2-4 Izinsuku)
◯ Isikhathi Esiningi Kakhulu (5-7 Izinsuku)

Ngesonto eledlule ngangingalele kahle (ngavuka ebusuku/angilalanga kahle/ngaba nenkinga yokuzumeka)

◯ Akujwayele Noma Asikho Isikhathi
◯ Esinye Sesikhathi Esincane (1-2 Izinsuku)
◯ Inani Lesikhathi Elikahle (2-4 Izinsuku)
◯ Isikhathi Esiningi Kakhulu (5-7 Izinsuku)

Ngesonto eledlule ngangijabule

◯ Akujwayele Noma Asikho Isikhathi
◯ Esinye Sesikhathi Esincane (1-2 Izinsuku)
◯ Inani Lesikhathi Elikahle (2-4 Izinsuku)
◯ Isikhathi Esiningi Kakhulu (5-7 Izinsuku)

Ngesonto eledlule ngakhuluma kancane kunokwejwayelekile

◯ Akujwayele Noma Asikho Isikhathi ◯ Esinye Sesikhathi Esincane (1-2 Izinsuku) ◯ Inani Lesikhathi Elikahle (2-4 Izinsuku) ◯ Isikhathi Esiningi Kakhulu (5-7 Izinsuku)

Ngesonto eledlule ngazizwa ngingedwa

◯ Akujwayele Noma Asikho Isikhathi ◯ Esinye Sesikhathi Esincane (1-2 Izinsuku) ◯ Inani Lesikhathi Elikahle (2-4 Izinsuku) ◯ Isikhathi Esiningi Kakhulu (5-7 Izinsuku)

Ngesonto eledlule abantu babengenabo ubungane

◯ Akujwayele Noma Asikho Isikhathi ◯ Esinye Sesikhathi Esincane (1-2 Izinsuku) ◯ Inani Lesikhathi Elikahle (2-4 Izinsuku) ◯ Isikhathi Esiningi Kakhulu (5-7 Izinsuku)

Ngesonto eledlule ngayithokozela impilo

◯ Akujwayele Noma Asikho Isikhathi ◯ Esinye Sesikhathi Esincane (1-2 Izinsuku) ◯ Inani Lesikhathi Elikahle (2-4 Izinsuku) ◯ Isikhathi Esiningi Kakhulu (5-7 Izinsuku)

Ngesonto eledlule ngikhale kakhulu

◯ Akujwayele Noma Asikho Isikhathi ◯ Esinye Sesikhathi Esincane (1-2 Izinsuku) ◯ Inani Lesikhathi Elikahle (2-4 Izinsuku) ◯ Isikhathi Esiningi Kakhulu (5-7 Izinsuku)

Ngesonto eledlule ngazizwa ngigula

◯ Akujwayele Noma Asikho Isikhathi ◯ Esinye Sesikhathi Esincane (1-2 Izinsuku) ◯ Inani Lesikhathi Elikahle (2-4 Izinsuku) ◯ Isikhathi Esiningi Kakhulu (5-7 Izinsuku)

Ngesonto eledlule ngezwa ukuthi abantu abangithandi

◯ Akujwayele Noma Asikho Isikhathi ◯ Esinye Sesikhathi Esincane (1-2 Izinsuku) ◯ Inani Lesikhathi Elikahle (2-4 Izinsuku) ◯ Isikhathi Esiningi Kakhulu (5-7 Izinsuku)

Ngesonto eledlule angikwazanga 'ukuqhubeka'

◯ Akujwayele Noma Asikho Isikhathi ◯ Esinye Sesikhathi Esincane (1-2 Izinsuku) ◯ Inani Lesikhathi Elikahle (2-4 Izinsuku) ◯ Isikhathi Esiningi Kakhulu (5-7 Izinsuku)

Ngesonto eledlule bengicabanga kakhulu

◯ Akujwayele Noma Asikho Isikhathi ◯ Esinye Sesikhathi Esincane (1-2 Izinsuku) ◯ Inani Lesikhathi Elikahle (2-4 Izinsuku) ◯ Isikhathi Esiningi Kakhulu (5-7 Izinsuku)

Ngesonto eledlule inhliziyo yami beyibuhlungu

◯ Akujwayele Noma Asikho Isikhathi ◯ Esinye Sesikhathi Esincane (1-2 Izinsuku) ◯ Inani Lesikhathi Elikahle (2-4 Izinsuku) ◯ Isikhathi Esiningi Kakhulu (5-7 Izinsuku)

Ngesonto eledlule umoya wami bewuphansi

◯ Akujwayele Noma Asikho Isikhathi ◯ Esinye Sesikhathi Esincane (1-2 Izinsuku) ◯ Inani Lesikhathi Elikahle (2-4 Izinsuku) ◯ Isikhathi Esiningi Kakhulu (5-7 Izinsuku)

Manje ngifuna ukukubuza umbuzo mayelana nenyanga edlule (amasonto amane), Ingabe umcabango wokuzibulala bewusengqondweni yakho?

○ Cha ○ Yebo

The following are symptoms that people sometimes experience after having a terrible event happen in their lives. I'll read each one, please state how much the symptom has bothered you in the past week

Ukubuyelela kwemicabango noma izinkumbulo zesigameko

○ Lutho nhlobo ○ Akujwayele ○ Ngezinye izikhathi ○ Izikhathi Eziningi Kakhulu

Kuzwakala sengathi isehlakalo siyenzeka futhi

○ Lutho nhlobo ○ Akujwayele ○ Ngezinye izikhathi ○ Izikhathi Eziningi Kakhulu

Amaphupho amabi abuyelelayo maqondana nesehlakalo

○ Lutho nhlobo ○ Akujwayele ○ Ngezinye izikhathi ○ Izikhathi Eziningi Kakhulu

Ukuzwela kwemizwa noma okomzimba okusheshayo uma ngikhunjuzwa ngesigameko

○ Lutho nhlobo ○ Akujwayele ○ Ngezinye izikhathi ○ Izikhathi Eziningi Kakhulu

Ukugwema imisebenzi ekukhumbuza ngesigameko

○ Lutho nhlobo ○ Akujwayele ○ Ngezinye izikhathi ○ Izikhathi Eziningi Kakhulu

Ukugwema imicabango noma imizwa ehambisana nesigameko, ngizama ukuthi ngingezwa noma ngingacabangi nganoma iyiphi into engikhumbuza sona

○ Lutho nhlobo ○ Akujwayele ○ Ngezinye izikhathi ○ Izikhathi Eziningi Kakhulu

Ukuzizwa wethuka, wesaba kalula

○ Lutho nhlobo ○ Akujwayele ○ Ngezinye izikhathi ○ Izikhathi Eziningi Kakhulu

Ukuzizwa uhlala uqaphile, ukulungele ukulwa ukuze uzivikele wena uqobo

○ Lutho nhlobo ○ Akujwayele ○ Ngezinye izikhathi ○ Izikhathi Eziningi Kakhulu

Esikhathini esedlule, uke wazisika, wazishisa noma wazilimaza wena uqobo ngenhloso?

○ Yebo ○ Cha

Ngonyaka owedlule, uke wazama ukuzibulala?

○ Yebo ○ Cha

Audit

How often do you have a drink containing alcohol?

◯ Never ◯ Monthly Or Less ◯ 2-4Times Per Month ◯ 2-3 Times Per Week ◯ 4+ Times Per Week

How many drinks containing alcohol do you have on a typical day when you are drinking?

◯ 0 ◯ 1 Or 2 ◯ 3 Or 4 ◯ 5 Or 6 ◯ 7 Or 9 ◯ 10 Or More

How much is a typical drink (one drink, a beer or a glass of wine etc)

◯ no drink ◯ 250 mls or less (Small Beer or 1 glass of wine) ◯ 440mls ◯ 500mls
◯ 750 mls (Bumpie) ◯ 1 Litre ◯ 2 Litres ◯ 5 Litres (Big Box Wine)

How often do you have six or more drinks on one occasion?

◯ Never ◯ Less Than Monthly ◯ Monthly ◯ Weekly ◯ Daily Or Almost Daily

Within the past year how often have you taken dagga
(PLEASE SELECT ALL THAT APPLY)

◯ never ◯ once ◯ sometimes ◯ often

Within the past year how often have you taken Ndanda or mandrax

◯ never ◯ once ◯ sometimes ◯ often

Within the past year how often have you taken Nyaopi or whoonga

◯ never ◯ once ◯ sometimes ◯ often

Within the past year how often have you taken cough mixture when not sick

◯ never ◯ once ◯ sometimes ◯ often

Within the past year how often have you taken pain killers when not ill

◯ never ◯ once ◯ sometimes ◯ often

Within the past year how often have you taken Mg (Ecstacy)

◯ never ◯ once ◯ sometimes ◯ often

Within the past year how often have you taken Njumpi (Crystal Meth/Tik)|

◯ never ◯ once ◯ sometimes ◯ often

Within the past year how often have you taken Glass/Rock

◯ never ◯ once ◯ sometimes ◯ often

O nwa seno se nang le jwala ha kae?

◯ Ho hang ◯ Kgwedi le Kgwedi kapa Ka tlase ho moo ◯ Makgetlo a 2-4 ka Kgwedi ◯ Makgetlo a 2-3 ka Beke ◯ Makgetlo a 4+ ka Beke

Ke dino tse kae tse nang le jwala tseo o di nwang ka letsatsi?

◯ 0 ◯ 1 kapa 2 ◯ 3 kapa 4 ◯ 5 kapa 6 ◯ 7 kapa 9 ◯ 10 kapa ho feta

Seno se tlwaelehileng ke se se kae (seno se le seng, biya kapa kgalase ya waene, jj)

◯ no drink ◯ 250 mls kapa ka tlase (Biya e Nyane kapa kgalase e le 1 ya waene) ◯ 440mls
◯ 500mls ◯ 750 mls (Bumpie) ◯ Litara e le 1 ◯ Dilitara tse 2 ◯ Dilitara tse 5 (Waene ya Lebokose le Leholo)

Ke ka makgetlo a makae o nwang dino tse tsheletseng kapa ho feta ketsahalong e le nngwe?

◯ Ho hang ◯ Ka tlase ho Kgwedi le Kgwedi ◯ Kgwedi le Kgwedi ◯ Beke le Beke ◯ Letsatsi le Letsatsi kapa Nako e ka bang Letsatsi le Letsatsi

Selemong se fetileng, o tsubile matekwane ha kae?
(PLEASE SELECT ALL THAT APPLY)

◯ ho hang ◯ ha nngwe ◯ ka dinako tse ding ◯ kgafetsa

Selemong se fetileng, o tsubile Ndanda kapa mendrekse ha kae?

◯ ho hang ◯ ha nngwe ◯ ka dinako tse ding ◯ kgafetsa

Selemong se fetileng o tsubile Nyaope kapa whoonga ha kae?

◯ ho hang ◯ ha nngwe ◯ ka dinako tse ding ◯ kgafetsa

Selemong se fetileng, o nwele moriana wa ho kgohlela ha kae o sa kule?

◯ ho hang ◯ ha nngwe ◯ ka dinako tse ding ◯ kgafetsa

Selemong se fetileng, o nwele difodisamahlaba ha kae o sa kule?

◯ ho hang ◯ ha nngwe ◯ ka dinako tse ding ◯ kgafetsa

Selemong se fetileng, o tsubile Mg (Ecstacy) ha kae?

◯ ho hang ◯ ha nngwe ◯ ka dinako tse ding ◯ kgafetsa

Selemong se fetileng, o tsubile Njumpi (Crystal Meth/Tiki) ha kae?

◯ ho hang ◯ ha nngwe ◯ ka dinako tse ding ◯ kgafetsa

Selemong se fetileng, o tsubile Glass/Rock ha kae?

◯ ho hang ◯ ha nngwe ◯ ka dinako tse ding ◯ kgafetsa

Usiphuza kaningi kangakanani isiphuzo esinotshwala?

◯ Neze nhlobo ◯ Ngenyanga Noma Ngaphansi ◯ 2-4Izikhathi Ngenyanga ◯ 2-3 Izikhathi Ngesonto
◯ 4+ Izikhathi Ngesonto

Uphuza iziphuzo ezingaki ezinotshwala ngelanga elejwayelekile uma uphuza?

◯ 0 ◯ 1 Noma 2 ◯ 3 Noma 4 ◯ 5 Noma 6 ◯ 7 Noma 9 ◯ 10 Noma Ngaphezulu

Isiphuzo esijwayelekile singakanani (isiphuzo esisodwa, ubhiya noma ingilazi yewayini, njll.)?

◯ no drink ◯ 250 mls noma ngaphansi (Ubhiya Omncane noma 1 ingilazi yewayini) ◯ 440mls
◯ 500mls ◯ 750 mls (Bumpie) ◯ 1 Litha ◯ 2 amaLitha ◯ 5 amaLitha (Ibhokisi Elikhulu Lewayini)

Kukaningi kangakanani lapho ophuza khona iziphuzo eziyisithupha noma ngaphezulu ngesikhathi esisodwa?

◯ Neze nhlobo ◯ Ngaphansi KwangeNyanga ◯ Ngenyanga ◯ Ngesonto ◯ Nsuku zonke Noma Cishe Nsuku Zonke

Onyakeni odlule, uyibheme kaningi kangakanani insangu?
(PLEASE SELECT ALL THAT APPLY)

◯ Neze nhlobo ◯ kanye ◯ ngezinye izikhathi ◯ kaningi

Onyakeni odlule, uyibheme kaningi kangakanani iNdanda noma ama-mandrax?

◯ Neze nhlobo ◯ kanye ◯ ngezinye izikhathi ◯ kaningi

Onyakeni odlule, uyibheme kaningi kangakanani iNyaopi noma i-whoonga?

◯ Neze nhlobo ◯ kanye ◯ ngezinye izikhathi ◯ kaningi

Onyakeni odlule, uwuphuze kaningi kangakanani umuthi wokukhwehlela ungaguli?

◯ Neze nhlobo ◯ kanye ◯ ngezinye izikhathi ◯ kaningi

Onyakeni odlule, uwaphuze kaningi kangakanani amaphilisi ezinhlungu ungaguli?

◯ Neze nhlobo ◯ kanye ◯ ngezinye izikhathi ◯ kaningi

Onyakeni odlule, uyibheme kaningi kangakanani i-Mg (Ecstacy)?

◯ Neze nhlobo ◯ kanye ◯ ngezinye izikhathi ◯ kaningi

Onyakeni odlule, uyibheme kaningi kangakanani i-Njumpi (Crystal Meth/Tik)?

◯ Neze nhlobo ◯ kanye ◯ ngezinye izikhathi ◯ kaningi

Onyakeni odlule, uyibheme kaningi kangakanani i-Glass/Rock?

◯ Neze nhlobo ◯ kanye ◯ ngezinye izikhathi ◯ kaningi

Self Esteem

For each statement please select either strongly agree, agree, disagree or strongly disagree.

Most of the time I am satisfied with myself

○ Strongly Agree ○ Agree ○ Disagree ○ Strongly Disagree

At times, I think I am no good at all

○ Strongly Agree ○ Agree ○ Disagree ○ Strongly Disagree

I feel that I have a number of good qualities

○ Strongly Agree ○ Agree ○ Disagree ○ Strongly Disagree

I am able to do things as well as most other people

○ Strongly Agree ○ Agree ○ Disagree ○ Strongly Disagree

I feel I do not have much to be proud of

○ Strongly Agree ○ Agree ○ Disagree ○ Strongly Disagree

I certainly feel useless at times

○ Strongly Agree ○ Agree ○ Disagree ○ Strongly Disagree

I feel that I'm a person of worth, at least equal or as good as other people

○ Strongly Agree ○ Agree ○ Disagree ○ Strongly Disagree

I wish I could have more respect for myself

○ Strongly Agree ○ Agree ○ Disagree ○ Strongly Disagree

Most of the time I feel as if I am a failure

○ Strongly Agree ○ Agree ○ Disagree ○ Strongly Disagree

I take a positive attitude toward myself

○ Strongly Agree ○ Agree ○ Disagree ○ Strongly Disagree

For each statement please select either strongly agree, agree, disagree or strongly disagree.

Boholo ba nako ke kgotsofetse ka seo ke leng sona.

○ Ke Dumela Ke Tiile ○ Ke a Dumela ○ Ke a Hana ○ Ke Hana ke Tiile

Ka dinako tse ding ke nahana hore ha ke na molemo ho hang

◯ Ke Dumela Ke Tiile ◯ Ke a Dumela ◯ Ke a Hana ◯ Ke Hana ke Tiile

Ke ikutlwa eka ke na le dintho tse mmalwa tse ntle ka nna

◯ Ke Dumela Ke Tiile ◯ Ke a Dumela ◯ Ke a Hana ◯ Ke Hana ke Tiile

Ke kgona ho etsa dintho mmoho le boholo ba batho

◯ Ke Dumela Ke Tiile ◯ Ke a Dumela ◯ Ke a Hana ◯ Ke Hana ke Tiile

Ke ikutlwa eka ha ke na dintho tse ngata tse nketsang motlotlo

◯ Ke Dumela Ke Tiile ◯ Ke a Dumela ◯ Ke a Hana ◯ Ke Hana ke Tiile

Bonneteng ke ikutlwa ke se na molemo ka dinako tse ding

◯ Ke Dumela Ke Tiile ◯ Ke a Dumela ◯ Ke a Hana ◯ Ke Hana ke Tiile

Ke ikutlwa ke le motho wa bohlokwa, bonyane ho lekana kapa ho ba bohlokwa jwalo ka batho ba bang

◯ Ke Dumela Ke Tiile ◯ Ke a Dumela ◯ Ke a Hana ◯ Ke Hana ke Tiile

Ke lakatsa eka nka itlhompha ho feta

◯ Ke Dumela Ke Tiile ◯ Ke a Dumela ◯ Ke a Hana ◯ Ke Hana ke Tiile

Boholo ba nako ke ikutlwa eka ke hloleha ho atleha

◯ Ke Dumela Ke Tiile ◯ Ke a Dumela ◯ Ke a Hana ◯ Ke Hana ke Tiile

Ke ba le mekgwa e phosethifi ya ho nahana ka nna

◯ Ke Dumela Ke Tiile ◯ Ke a Dumela ◯ Ke a Hana ◯ Ke Hana ke Tiile

---

For each statement please select either strongly agree, agree, disagree or strongly disagree.

Kukho konke, nganelisekile ngalokho engiyikho.

◯ Ngiyavuma Kakhulu ◯ Ngiyavuma ◯ Angivumi ◯ Angivumi Kakhulu

Ngezinye izikhathi, ngicabanga ukuthi angikho kahle nhlobo

◯ Ngiyavuma Kakhulu ◯ Ngiyavuma ◯ Angivumi ◯ Angivumi Kakhulu

Ngicabanga ukuthi nginezinto eziseqophelweni eliphezulu ezinhle eziningana

◯ Ngiyavuma Kakhulu ◯ Ngiyavuma ◯ Angivumi ◯ Angivumi Kakhulu

Ngiyakwazi ukwenza izinto njengabantu abanye abaningi

◯ Ngiyavuma Kakhulu ◯ Ngiyavuma ◯ Angivumi ◯ Angivumi Kakhulu

Ngicabanga ukuthi anginakho okuningi engingaziqhenya ngakho

◯ Ngiyavuma Kakhulu ◯ Ngiyavuma ◯ Angivumi ◯ Angivumi Kakhulu

Ngokuseqinisweni ngizizwa ngingenamsebenzi walutho ngezinye izikhathi

◯ Ngiyavuma Kakhulu
◯ Ngiyavuma
◯ Angivumi
◯ Angivumi Kakhulu

Ngicabanga ukuthi ngingumuntu obalulekile, okungenani olinganayo noma omuhle njengabanye abantu

◯ Ngiyavuma Kakhulu
◯ Ngiyavuma
◯ Angivumi
◯ Angivumi Kakhulu

Ngifisa sengathi ngingazihlonipha kakhulu

◯ Ngiyavuma Kakhulu
◯ Ngiyavuma
◯ Angivumi
◯ Angivumi Kakhulu

Esikhathini esiningi ngizizwa sengathi ngiyisehluleki

◯ Ngiyavuma Kakhulu ◯ Ngiyavuma ◯ Angivumi ◯ Angivumi Kakhulu

Indlela yokucabanga enhle ngiyibhekisa kimi

◯ Ngiyavuma Kakhulu ◯ Ngiyavuma ◯ Angivumi ◯ Angivumi Kakhulu

Final

END OF INTERVIEW

I would like to thank you very much for helping us. We have talked about some very difficult things today. I appreciate the time you have taken. I realise that these questions may have been difficult for you to answer, but it is only by hearing about women?s lives that we can really begin to understand them. We really appreciate your openness with us. Most women have difficult times in their lives and its good to share them and remember we did not bring them on ourselves. We really appreciate your participation in this study. By sharing this personal information with us and attending the SSWP peer talks and creative spaces you are helping us with our research and that will ultimately help many other sex workers in the country.

In the past 6 months have you received either of these items (SHOW A RED UMBRELLA CONDOM AND LUBRICANT) from a PE?

◯ Yes
◯ No

Last year, between January 2015 and December 2015, did you come to the SSWP clinic and do an HIV test? (ONLY YES IF THEY DID AN HIV TEST. DO NOT NOTE YES IF THEY DID ANYTHING ELSE WITH US!!!!!)

◯ Yes ◯ No

Could we contact you for any future research studies which may either be related to or unrelated to this study?

◯ No ◯ Yes

END OF INTERVIEW

Ke rata ho o leboha haholo ka ho re thusa. Re buile ka dintho tse itseng tse boima haholo kajeno. Ke thabela nako eo o bileng le yona. Ke elellwa hore dipotso tsena di ka be di bile boima hore o di arabe, empa ke ka ho utlwa ka maphelo a basadi re ka qalang ho a utlwisisa. Re thabela ho phuthulloha ha hao ho rona e le ka nnete. Basadi ba bangata ba imetswe maphelong a bona mme ke ntho e ntle ho bua ka oona le ho hopola hore ha re a ipitsetsa oona. Re thabela ho nka karolo ha hao phuputsong ena e le ka nnete. Ka ho bua le rona ka ditaba tsena tsa bophelo ba hao le ho kenela dipuisano tsa bomphato tsa SSWP le dicreative space, o re thusa ka phuputso ya rona mme hoo ho tla qetella ho thusa disex worker tse ding tse ngata naheng.

Na re ka iteanya le wena bakeng sa phuputso efe kapa efe ya nako e tlang e ka beng e amana kapa e sa amane le phuputso ena?

◯ Tjhe ◯ E

END OF INTERVIEW

Ngiyabonga kakhulu ngokuthi usisize. Sikhulume ngezinto ezinzima kakhulu namhlanje. Ngiyathokoza ngesikhathi osithathile. Ngiyazi ukuthi le mibuzo kungenzeka ukuthi beyinzima ukuthi uyiphendule, kodwa wukuzwa ngezimpilo zabantu besifazane kuphela okungasenza ukuthi siqale ukubaqonda. Siyakuthokozela kakhulu ukuvuleleka kwakho kithi. Abesifazane abaningi kakhulu banezikhathi ezinzima ezimpilweni zabo futhi kuhle ukushiyelana nabo futhi khumbula ukuthi asibalethanga kithi. Siyakuthokozela kakhulu ukuhlanganyela kwakho kulolu cwaningo. Ngokushiyelana ngalolu lwazi lomuntu siqu nokuza ezingxoxweni zontanga ze-SSWP nasezindaweni zokusungula osisiza ngocwaningo lwethu nokuthi ekugcineni luyosiza amanye ama-sex worker amaningi ezweni.

Singaxhumana nawe ngananoma yiziphi izingcwaningo zohlolo lwesikhathi esizayo okungenzeka luhambisane noma lungahambisani nalolu cwaningo?

◯ Cha ◯ Yebo

End Time of Interview

____

This interview took ___minutes to complete

____
(INSERT CALC FOR DATE TIME)

Hct

HIV COUNSELLING and TESTING

Thank you, for taking this survey with me. Now that we have completed the survey, I am going to to explain what an HIV test is and what the results mean. I will then test you and while we wait for the results we can discuss ways to reduce your risk. You have already signed consent to do the test, so we will not need to sign consent again now.

Pre Counselling Received
(EXPLAIN THE HCT PROCESS Two rapid test for HIV will be done by either myself or the nurse. A few drops of blood will be collected and tested on specific HIV testing kits to check for HIV antibodies. Test results will be ready in about 10 minutes. If both tests show us a negative result then it means that there are no antibodies to HIV. (Counsellor explains again about the window period). If both tests are positive, it means that you are HIV positive and that there are antibodies to HIV. Sometimes we cannot clearly tell if the results are negative or positive, so we have have 1 test show us a positive and the other show us a negative result. We will then have the nurse draw blood which we will send through to the laboratory for an HIV test called an ELISA to confirm the results. If you are HIV positive, they will also do your CD4 count and viral load. You will be able to get your laboratory results back from us in a week.)

○ Yes ○ No

First Rapid Test

○ Reactive (Positive) ○ Non-Reactive (Negative) ○ Indeterminate

Second Rapid Test

○ Reactive (Positive) ○ Non-Reactive (Negative) ○ Indeterminate

HIV NEGATIVEno further biological tests required. Participant can now return to coupon manager

HIV POSITIVE RESULTinform participant of their status and conduct CD4 and viral load. Once completed participant can return to coupon manager

INDETERMINATE RESULTCall nurse to draw blood and send to NHLS for HIV test (& CD4, Viral load). Once blood drawn participant can move on to coupon manager

Did you negotiate a risk reduction plan
(IDENTIFY RISK REDUCTION BEHAVIOURS. IT IS VERY IMPORTANT THAT THE PARTICIPANT UNDERSTANDS THAT THE USE OF CONDOMS WITH INTIMATE PARTNERS IS AS IMPORTANT AS IT IS WITH MCIMBI IN PROTECTING THEMSELVES, THEIR PARTNER AND THEIR CLIENTS, FROM HIV AND STIS. EXPLORE RISKY SEX WORKER BEHAVIOURS MENTIONED IN THEIR QUESTIONNAIRE AND WHICH THEY ARE ABLE & MOTIVATED TO CHANGE. IDENTIFY A STEP TOWARDS CHANGING THIS BEHAVIOUR. BREAK THE BEHAVIOUR DOWN INTO CONCRETE STEPS FOR THE CLIENT TO TRY. CHECK IN WITH THE CLIENT TO ENSURE THAT THE PLAN IS ACCEPTABLE AND ACTIONABLE FOR THEM - THEY SHOULD TAKE OWNERSHIP OF THE PLAN. YOU CAN ROLE PLAY THE PLAN TO ASSIST THE CLIENT. ASK THE CLIENT TO TRY THE PLAN OUT. MAKE SURE THEY KNOW THEY CAN COME BACK TO US FOR FUTURE ADVICE OR COUNSELLING TO ADAPT THIS PLAN. RECOGNISE HOW HARD IT CAN BE TO CHANGE BEHAVIOUR AND THE DANGERS SURROUNDING SEX WORK, BUT THAT THE PROGRAMME IS HERE TO SUPPORT THEM. IF THEY ARE NOT FAMILIAR WITH SSWP, PLEASE TELL THEM ABOUT US AND INVITE THEM TO COME TO OUR CREATIVE SPACE WORKSHOPS OR TAKE THEIR DETAILS DOWN TO DO OUTREACH IN THEIR AREA (SOWETO ONLY))

○ Yes ○ No

COMPLETED BY THE NURSE

Tests sent
(ONLY SEND UNKNOWN POSITIVES TO CLS)

☐ Confirmatory Elisa assay (NHLS) ☐ CD4 (NHLS) ☐ Viral Load (CLS) ☐ Drug Resistance (NICD)

CLS Laboratory Bar Code

____

NHLS Laboratory Bar Code

____

Confirmatory Elisa Result

◯ Negative ◯ Positive

CD4 count result

____
(NUMBER)

Viral Load result

____

Coupons

COUPON MANAGEMENT

This must be completed by the coupon manager for EVERY coupon given to this participant.

Number of coupons provided

◯ 0
◯ 1
◯ 2
◯ 3

Coupon 1 Number

____
(CHECK COUPON NUMBER AGAINST COUPON MANAGER SPREADSHEET. MAKE SURE YOU ACCURATELY COPY NUMBER ONTO COUPON!)

Coupon 2 Number

____
(CHECK COUPON NUMBER AGAINST COUPON MANAGER SPREADSHEET. MAKE SURE YOU ACCURATELY COPY NUMBER ONTO COUPON!)

Coupon 3 Number

____
(CHECK COUPON NUMBER AGAINST COUPON MANAGER SPREADSHEET. MAKE SURE YOU ACCURATELY COPY NUMBER ONTO COUPON!)

preferred reimbursement method

◯ Airtime ◯ Cash

valid mobile phone number

____
(PLEASE CONFIRM THAT THIS IS THE NUMBER WE WILL MAKE THEIR PAYMENT TO)

First two letters of your name, first two letters of your surname, year of birth, first two letters of your mothers' name ______________________________

B5 Art Drug Resistance

Drug Resistance Tested	◯ Yes<br>◯ No
Susceptible to all	◯ Yes<br>◯ No
Zidovudine	◯ S/PLLR ◯ LLR/IR ◯ HLR
Didanosine	◯ S/PLLR ◯ LLR/IR ◯ HLR
Stavudine	◯ S/PLLR ◯ LLR/IR ◯ HLR
Lamuvidine	◯ S/PLLR ◯ LLR/IR ◯ HLR
Emtricitabine	◯ S/PLLR ◯ LLR/IR ◯ HLR
Abacavir	◯ S/PLLR ◯ LLR/IR ◯ HLR
Tenofovir	◯ S/PLLR ◯ LLR/IR ◯ HLR
Nerviripine	◯ S/PLLR ◯ LLR/IR ◯ HLR
Efavirenz	◯ S/PLLR ◯ LLR/IR ◯ HLR
Etravirine	◯ S/PLLR ◯ LLR/IR ◯ HLR
Rilpivirine	◯ S/PLLR ◯ LLR/IR ◯ HLR
Indinavir/r	◯ S/PLLR ◯ LLR/IR ◯ HLR
Saquinavir/r	◯ S/PLLR ◯ LLR/IR ◯ HLR
Nelfinavir	◯ S/PLLR ◯ LLR/IR ◯ HLR
Fosamprenavir/r	◯ S/PLLR ◯ LLR/IR ◯ HLR
Lopinavir/r	◯ S/PLLR ◯ LLR/IR ◯ HLR
Atazanavir/r	◯ S/PLLR ◯ LLR/IR ◯ HLR
Tiplrnavir/r	◯ S/PLLR ◯ LLR/IR ◯ HLR
Darunavir/r	◯ S/PLLR ◯ LLR/IR ◯ HLR
